# Reduced Synaptophysin-like 2 (MG29/SYPL2) Levels Mimic Age-Related Alterations in Skeletal Muscle Calcium Homeostasis and Lipid Signaling

**DOI:** 10.3390/biom16070988

**Published:** 2026-07-04

**Authors:** Kamal Awad, Jian Huang, Marian N. Aziz, Zhiying Wang, Leticia Brotto, Kyung Eun Lee, Jongsoo Kim, Rajendiran Karthikraj, Liubov V. Gushchina, Noah Weisleder, Marco Brotto

**Affiliations:** 1 Bone-Muscle Research Center, College of Nursing and Health Innovation, The University of Texas at Arlington, Arlington, TX 76019, USA; kamal.awad@uta.edu (K.A.);; 2 Molecular Imaging Program, Department of Radiology, School of Medicine, Stanford University, Stanford, CA 94305, USA; 3 Bristol Myers Squibb, Princeton, NJ 08540, USA; 4 Department of Surgery, School of Medicine, University of Virginia, Charlottesville, VA 22903, USA; 5 Jerry R. Mendell Center for Gene Therapy, Abigail Wexner Research Institute at Nationwide Children’s Hospital, Columbus, OH 43205, USA; 6 Department of Pediatrics, The Ohio State University, Columbus, OH 43210, USA; 7 Department of Molecular and Cellular Biochemistry, College of Medicine, University of Kentucky, Lexington, KY 40536, USA

**Keywords:** mitsugumin 29, MG29, sarcopenia, SOCE, calcium homeostasis, skeletal muscle, lipid signaling

## Abstract

Sarcopenia is characterized by progressive loss of skeletal muscle mass and function and is a major contributor to frailty, disability, and mortality in older adults. Store-operated calcium entry (SOCE) is a crucial regulator of skeletal muscle calcium homeostasis, and impaired SOCE has been linked to age-related muscle weakness. Here, we identify the synaptophysin family member synaptophysin-like protein 2, also known as mitsugumin 29 (MG29; encoded by the human gene *SYPL2* and the mouse ortholog *Mg29*), as a key organizer of triad membrane cholesterol and lipid signaling required for normal SOCE during aging. Using *Mg29*^−/−^ mice as a model of accelerated sarcopenia, together with RNA interference against *Mg29* in adult muscle and primary myotubes, we quantified changes in muscle morphology, contractile function, SOCE activity, and targeted lipidomic profiles. Reduced MG29 expression led to decreased muscle fiber cross-sectional area, reduced specific force, blunted SOCE, and marked alterations in membrane cholesterol content and fatty acid-derived lipid mediators. Cholesterol depletion by methyl-β-cyclodextrin in wild-type myotubes produced SOCE defects similar to those observed in aged wild-type and young *Mg29*^−/−^ muscles, indicating that MG29-dependent maintenance of membrane cholesterol is required for normal SOCE. Acute *Mg29* knockdown also altered myogenic differentiation, the expression of calcium-handling and stress-response genes, and the release and consumption of specific polyunsaturated fatty acid-derived lipid mediators. Together, these findings identify MG29 as a critical regulator of SOCE and lipid signaling in skeletal muscle and suggest that its age-related decline contributes to sarcopenia by disrupting triad membrane organization and excitation–contraction coupling.

## 1. Introduction

The average global population age is rapidly increasing, and the proportion of people aged 60 years and older is projected to almost double between 2006 and 2050 according to Word Health Organization (WHO). Aging is a complex biological process that affects multiple organ systems and is characterized by genomic instability, telomere attrition, epigenetic alterations, loss of proteostasis, and deregulated nutrient sensing [1]. In skeletal muscle, normal aging leads to sarcopenia, defined as a progressive loss of muscle mass and strength or power that is only partially mitigated by increased physical activity or improved diet [1,2].

Sarcopenia affects more than 30% of adults over 60 years of age and is associated with frailty, functional impairment, physical disability, and increased mortality, contributing substantially to healthcare costs [3,4,5]. In addition to reduced muscle size and fiber number, sarcopenia is characterized by selective loss of type II fibers, reduced satellite cell content, and a decline in specific force, indicating that mechanisms beyond atrophy contribute to muscle weakness [6,7,8,9,10]. Interventions that primarily target functional restoration rather than muscle mass alone are more effective in older adults, underscoring the need to understand cellular mechanisms that impair muscle quality [1,11,12,13]. Multiple processes have been implicated in the atrophy-independent loss of muscle function with aging, including reduced neural drive, increased non-contractile tissue, altered cross-bridge function and Ca^2+^ sensitivity, and disrupted excitation–contraction (E-C) coupling [14,15,16,17,18,19,20]. Our group and others have identified store-operated Ca^2+^ entry (SOCE) as a physiologically relevant Ca^2+^ influx pathway in skeletal muscle, activated by the depletion of sarcoplasmic reticulum Ca^2+^ stores through coordinated actions of STIM1 and Orai channels. SOCE is essential for refilling sarcoplasmic reticulum Ca^2+^ during repetitive activity, and its dysregulation has been linked to impaired force production and myopathic phenotypes [14,16,21,22,23].

We previously demonstrated that SOCE is significantly blunted in muscles from aged mice and that this reduction contributes to the age-related decline in muscle-specific force. We also identified mitsugumin 29 (MG29, now designated as synaptophysin-like 2, SYPL2) as a triad junction protein whose levels decline in aged skeletal muscle and whose loss recapitulates several features of aging, including reduced SOCE and impaired contractile performance [24,25]. MG29 belongs to the synaptophysin/MARVEL family, contains four transmembrane domains, and localizes to both transverse tubule and sarcoplasmic reticulum membranes, where it participates in triad organization and lipid homeostasis [24,25,26,27].

Understanding the specific mechanisms that contribute to sarcopenia is essential for effective interventions during aging [20,28,29]. Several studies establish potential mechanisms for this discrepancy between atrophy-dependent vs. atrophy-independent loss of muscle function in aging, including reduced neural drive, increased non-contractile tissue, decreased myosin force and/or actin-myosin cross-bridge stability or sensitivity to Ca^2+^, and altered excitation–contraction (E-C) coupling [14,16,21,22,30]. SOCE is an extracellular Ca^2+^ entry pathway present in most cells, where the depletion of the intracellular store of Ca^2+^ in the endoplasmic or sarcoplasmic reticulum (ER/SR) triggers Ca^2+^ influx from the extracellular space via the coordinated recruitment of STIM1 and Orai1, 2, or 3. The role of SOCE in skeletal muscle was not initially appreciated. Our previous work and that of others indicate that SOCE is an essential regulator of muscle physiology and is linked to excess extracellular Ca^2+^ ([Ca^2+^] entry) in muscle pathology [15,31,32]. Recent studies from our labs and others have focused on understanding the age-related decreases in muscle strength resulting from a combination of loss of muscle mass (atrophy) and reduced muscle-specific force (i.e., muscle force per unit of cross-sectional area) [6,7]. We found that SOCE is significantly blunted in muscles from aged mice, and this reduction in SOCE appears to play an important role in the age-related decline of muscle force production.

Our previous work also determined that the MG29 protein is linked to decreased SOCE and force production in aging muscle [31]. While this protein was initially called MG29, it later received the designation of synaptophysin-like 2 (SYPL2), as it shares characteristic structural features with members of the synaptophysin and MARVEL families of proteins [25,33,34]. MG29/SYPL2 contains four transmembrane domains that allow the protein to localize at both the transverse tubule (TT) membrane and the SR membranes of the triad junction in skeletal muscle [17,35,36]. MG29/SYPL2 levels decrease in aging mouse muscle, and normal levels of MG29/SYPL2 protein are essential for the proper formation of the TT system in skeletal muscle, maintenance of lipid content of the sarcolemmal membrane, and efficient signaling between the ryanodine receptor type 1 (RyR1) and the SOCE machinery for the refilling of Ca^2+^ stores in SR. Interestingly, we also found that the knockout of sarcalumenin, an SR-resident Ca^2+^-binding protein, increased MG29 expression, enhanced SOCE, and improved muscle performance [36], suggesting that MG29 is directly involved in SOCE regulation, which in turn is essential for normal muscle function.

In the present study, we investigated how reduced MG29 expression contributes to age-related changes in skeletal muscle structure, function, and lipid signaling. Using *Mg29*^−/−^ mice, in vivo electroporation with *Mg29* siRNA, and *Mg29* knockdown in mouse primary myotubes, combined with targeted lipidomics and functional Ca^2+^ imaging, we tested the hypothesis that MG29-dependent maintenance of membrane cholesterol and lipid mediators is required for normal SOCE and muscle performance during aging. By integrating structural, functional, and lipidomic data, this study aimed to define the mechanisms by which MG29 loss mimics key aspects of sarcopenia and to identify potential targets for preserving muscle quality in older individuals.

## 2. Materials and Methods

### 2.1. Animals and Ethical Approval

*Mg29*-null (*Mg29*^−/−^) mice were generated as previously described [33]. All animal procedures were conducted in accordance with institutional guidelines and approved by the Ohio State University Institutional Animal Care and Use Committee (IACUC protocol#2012A00000120-R3, approved on 12 October 2015, with most recent renewal date on 19 October 2024) and the University of Texas at Arlington Institutional Animal Care and Use Committee (IACUC protocol # A2015.0009 and IACUC protocol # A16.004 approved by the UTA IACUC committee on 15 December 2015 and 2 December 2018, and renewed on 5 December 2024 and 3 January 2025, respectively). Male C57BL/6J wild-type (WT) and *Mg29*^−/−^ mice were maintained under standard housing conditions with *ad libitum* access to food and water. Mice were euthanized at the indicated ages by CO_2_ inhalation followed by cervical dislocation.

For the systemic metabolic impact of MG29 overexpression, Male C57BL/6J mice were fed a high-fat diet (HFD; 60% kcal from fat, Research Diets Inc., New Brunswick, NJ, USA) starting at 5 weeks of age, and they were maintained on this diet for 18 weeks to establish a diet-induced metabolic condition. After 18 weeks of HFD feeding, mice received a single intravenous injection of MyoAAV2A-MHCK7-hMG29 (3 × 10^13^ vg/kg). Mice remained on the HFD throughout the 5-week post-injection period until serum collection. Serum samples were collected at 5 weeks post-injection for biochemical analysis of total cholesterol and triglyceride levels. All animal procedures were performed in accordance with institutional guidelines and were approved by the University of Virginia Institutional Animal Care and Use Committee (IACUC; protocol # 4423-02-26, approved on 15 February 2023, renewed on 6 May 2026).

### 2.2. Reagents and Antibodies

Sixteen isotope-labeled lipid mediator internal standards were purchased from Cayman Chemical (Ann Arbor, MI, USA), including AA-d_8_, 6-keto-PGF_1_α-d_4_, PGF_2_α-d_4_, PGE_2_-d_4_, PGD_2_-d_4_, TXB_2_-d_4_, LTB_4_-d_4_, LTC_4_-d_5_, 5-HETE-d_8_, 15-HETE-d_8_, 12-HETE-d_8_, PAF C-16-d_4_, tetranor-PGEM-d_6_, OEA-d_4_, DHA-d_5_, and EPA-d_5_. Formic acid (reagent grade, ≥95%) was obtained from Sigma-Aldrich (St. Louis, MO, USA). HPLC-MS grade acetonitrile, water, methanol, and ethanol were purchased from J.T. Baker (Phillipsburg, NJ, USA).

Cell-culture reagents were obtained as follows: penicillin–streptomycin (P/S; 10,000 U/mL each), DMEM high-glucose media, α-MEM media, and trypsin-EDTA (1×) from Mediatech Inc. (Manassas, VA, USA); Ham’s F-10 from Corning (Corning, NY, USA); fetal bovine serum (FBS), horse serum (HS), and caffeine from Thermo Fisher Scientific Inc. (Waltham, MA, USA); bovine serum albumin (BSA) and 4′,6-diamidino-2-phenylindole (DAPI) from Sigma-Aldrich; rat-tail collagen type I from BD Biosciences (San Jose, CA, USA); 16% paraformaldehyde from Alfa Aesar (Ward Hill, MA, USA); Entactin-Collagen IV-Laminin (ECL) gel from the indicated supplier; Lipofectamine RNAiMAX Transfection Reagent from ThermoFisher Scientific; and basic recombinant human fibroblast growth factor (bFGF) from Promega (Madison, WI, USA). Pronase was from EMD Millipore. Fura-2/AM was obtained from Life Technologies (Grand Island, NY, USA).

Custom *Mg29* siRNA and negative control siRNA were synthesized by Integrated DNA Technologies (Coralville, IA, USA). Tri Reagent was obtained from Molecular Research Center Inc. (Cincinnati, OH, USA). The High-Capacity cDNA Reverse Transcription Kit was from Applied Biosystems (Foster City, CA, USA). RT^2^ Real-Time SYBR Green/ROX PCR Master Mix was from SABiosciences (Valencia, CA, USA).

Carboxyfluorescein (CFS)-conjugated mouse monoclonal anti-human myosin heavy chain (MHC) antibody was purchased from R&D Systems Inc. (Minneapolis, MN, USA) and has been previously validated [37]. C2C12 mouse myoblasts were obtained from the American Type Culture Collection (ATCC; Manassas, VA, USA).

### 2.3. C2C12 Myoblast Culture and Differentiation

C2C12 myoblasts were cultured as previously described [15,38]. Briefly, cells were maintained at 37 °C in a humidified 5% CO_2_ atmosphere in growth medium (GM) consisting of DMEM/high glucose supplemented with 10% FBS and P/S (100 U/mL each) and were maintained at 40–70% confluence. For experiments, cells were plated at 1 × 10^5^ cells per well in 6-well plates, and medium was changed every 48 h. To induce differentiation into myotubes, cells at approximately 75% confluence were switched to differentiation medium (DM) consisting of DMEM/high glucose supplemented with 2% HS and P/S. Fully differentiated, functional myotubes formed within 5–7 days, and the medium was changed every 48 h during differentiation.

### 2.4. Primary Mouse Myoblast Isolation and Culture

Primary myoblasts were isolated from the hindlimb muscles of 5-month-old C57BL/6J mice. Harvested muscles were minced and digested with 0.1% pronase. Isolated cells (fibroblasts and myoblasts) were maintained and expanded in collagen I-coated T-75 flasks in growth medium consisting of Ham’s F-10 supplemented with 20% FBS, P/S (100 U/mL each), and 5 ng/mL bFGF for 3–4 weeks for purification. Myoblasts with ≥99% purity, as confirmed by immunostaining for MyoD, were used for experiments. Primary myoblasts were cultured following established protocols [39]. Cells were grown at 37 °C in a 5% CO_2_ atmosphere in skeletal muscle cell growth medium and maintained at 50–70% confluence. For experiments, cells were plated at 2 × 10^5^ cells per well in 6-well plates coated with ECL, and the medium was changed every 48 h. To induce differentiation, cells at 80% confluence were switched to DM (DMEM/high glucose + 2% HS + P/S). Fully differentiated, functional myotubes formed within 2–3 days, and the medium was changed every 48 h.

### 2.5. siRNA Transfection

Primary myoblasts were plated at 2 × 10^5^ cells per well in 6-well plates coated with ECL, allowed to attach for 2 h in growth medium, then switched to DM and cultured overnight. Cells were transfected using Lipofectamine RNAiMAX according to the manufacturer’s instructions. Experimental cells received 10 nM *MG29* siRNA plus 7.5 µL Lipofectamine RNAiMAX reagent, while control cells received 10 nM negative control siRNA plus 7.5 µL reagent. After transfection, cells were cultured in DM and allowed to differentiate for 3 days.

### 2.6. Immunostaining and Cell Morphometry

Cells were fixed with 10% neutral buffered formalin and permeabilized with 0.1% Triton X-100 in PBS as previously described [31,32,37,38]. Myosin heavy chain (MHC) was detected using CFS-conjugated anti-MHC antibody (1:50) for 30 min at room temperature, and nuclei were counterstained with DAPI. Fluorescent images were acquired using a Leica DMi8 fluorescence microscope (10× or 20× objective), an inverted fluorescence microscope (Leica Microsystems CMS GmbH, Ernst-Leitz-Str., Wetzlar, Germany), equipped with a Hamamatsu digital camera (Model C11440-22CU, HAMAMATSU Photonics K. K., Hamamatsu, Japan). Images were acquired and analyzed using LAS X software (Version 3.9.0.28093, Leica Microsystems, Wetzlar, Germany). To quantify myogenic differentiation, the fusion index (FI) was calculated as follows: (nuclei within MHC-expressing myotubes/total number of myogenic nuclei) × 100 [33]. Three independent experiments were performed, with three randomly selected areas per well. Approximately 2000 nuclei per area were analyzed.

### 2.7. RNA Isolation and Real-Time Quantitative PCR (RT-qPCR)

Total RNA was isolated using Tri Reagent according to the manufacturer’s protocol, and cDNA was synthesized using the High-Capacity cDNA Reverse Transcription Kit. RT-qPCR was performed using RT^2^ Real-Time SYBR Green/ROX PCR Master Mix. Primers used in this study are summarized in Table 1. RT-qPCR reactions (25 µL) were run in 96-well plates using a StepOnePlus instrument (Applied Biosystems). Data were analyzed using RT^2^ Profiler PCR Array Data Analysis software (SABiosciences/QIAGEN, version 3.5). CT values were normalized to *Gapdh* (glyceraldehyde-3-phosphate dehydrogenase) as the reference gene. Gene expression was calculated as fold-change relative to controls. The reactions were performed at least two times (duplicates), and all experiments were repeated at least three times.

A custom muscle-specific RT-qPCR array from SABiosciences was used to simultaneously detect gene expression changes after *MG29* siRNA transfection. cDNA was synthesized using the RT^2^ First Strand Kit (which includes genomic DNA elimination), and the PCR array was run according to the manufacturer’s protocol with a threshold of 0.25. Single peaks in melting curves validated each gene tested. Data were analyzed using RT^2^ Profiler PCR Array Data Analysis Software, with Ct values normalized to six built-in reference housekeeping genes, genomic DNA control, reverse transcription control, and positive PCR control. Statistical significance was set at a 2-fold difference in gene expression.

### 2.8. Cholesterol Depletion with Methyl-β-Cyclodextrin (MβCD)

C2C12 myotubes were treated with 5 mM MβCD for 30 min to partially deplete membrane cholesterol [40], while primary myotubes from 5-month-old WT mice were treated with 2 mM MβCD for 30 min to minimize sarcolemma disruption. After treatment, SOCE measurements were performed by Fura-2/AM Ca^2+^ imaging as described below.

### 2.9. MG29 Mutant Constructs

*Mg29* loss-of-function mutants were generated by site-directed mutagenesis. The *Mg29*-4FA mutant contained alanine (A) substitutions at four conserved phenylalanine (F) residues (one in each transmembrane span of the MARVEL domain). The *Mg29*-5CG mutant contained glycine (G) substitutions at all five cysteine (C) residues in the MARVEL domain. Wild-type (WT) and mutant *Mg29* constructs were transfected into C2C12 myoblasts and primary myotubes for functional analysis.

### 2.10. In Vivo Electroporation and MG29 Silencing in Intact Muscle

In vivo electroporation of flexor digitorum brevis (FDB) muscles was performed as previously described for siRNA/DNA delivery to this muscle [41]. Briefly, scrambled control siRNA or Mg29-targeting siRNA was injected into FDB muscles, followed by delivery of electrical pulses using the previously established parameters. Muscles were harvested 28 days post-electroporation for western blot analysis and SOCE functional assays.

### 2.11. Store-Operated Ca^2+^ Entry (SOCE) Measurements

SOCE was measured with Fura-2/AM ratiometric Ca^2+^ imaging on the Photon Technology International (PTI) system, following the manufacturer’s protocol as previously reported [42]. Briefly, cells or intact muscle fibers were loaded with 5 µM Fura-2/AM for 30 min at room temperature, then washed in physiological saline solution. SOCE was triggered by sarcoplasmic reticulum Ca^2+^ depletion using the protocol described for each experiment, followed by re-addition of 2 mM extracellular Ca^2+^. Fluorescence was measured at dual excitation wavelengths (340 and 380 nm) with emission at 510 nm using the indicated microscope and imaging system. The 340/380 ratio was calculated using the HORIBA PTI EasyRatioPro Software (Version 2.5.127.86) to reflect intracellular Ca^2+^ concentration. We presented the raw Fura-2/AM 340/380 ratio traces to illustrate the ΔSOCE response in C2C12 and primary muscle cells. We calculated intracellular Ca^2+^ concentrations derived from these ratios using the standard Grynkiewicz equation [43]. The standard Grynkiewicz equation is used to convert ratiometric fluorescence signals (e.g., from Fura-2/AM) into intracellular calcium concentration. SOCE was quantified as a ΔSOCE value, defined as the difference between the Ca^2+^ signal immediately before and at the peak after re-addition of extracellular Ca^2+^. In other words, ΔSOCE = (peak Ca^2+^ signal after Ca^2+^ re-addition) − (baseline Ca^2+^ signal immediately before Ca^2+^ re-addition).

For Mn^2+^ quenching assays in intact FDB fibers, SOCE was assessed by measuring the rate of Mn^2+^-induced quenching of Fura-2/AM fluorescence at 360 nm excitation following sarcoplasmic reticulum depletion.

### 2.12. Lipidomic Profiling of Gastrocnemius Muscle

Young WT and *Mg29*^−/−^ mice used for lipidomic profiling were 13–16 weeks old, and mid-aged WT and *Mg29*^−/−^ mice were 50–55 weeks old; these animals are therefore referred to as “young” and “mid-aged” throughout the text and figures. Gastrocnemius (GAS) muscles were snap-frozen in liquid nitrogen immediately after dissection and stored at −80 °C. For lipid extraction, 50–100 mg of muscle tissue was minced and homogenized in 1.0 mL ice-cold 80% methanol in water (*v*/*v*) with one stainless steel bead (5 mm; Qiagen, Germantown, MD, USA) using a TissueLyser II homogenizer (Qiagen) at 30 Hz for six 30 s bursts with 20 s intervals on ice. Homogenates were spiked with 5 µL of isotope-labeled lipid mediator internal standard (IS) stock solution (5 µg/mL for AA-d_8_; 2 µg/mL for DHA-d_5_ and EPA-d_5_; 0.5 µg/mL for all other IS), then agitated on ice in the dark for 1 h. Samples were centrifuged at 16,000× *g* for 10 min at 4 °C to remove tissue debris and precipitated proteins.

Supernatants were cleaned and concentrated by solid-phase extraction (SPE) as previously described [44]. Briefly, lipid mediators were fully protonated by adding ice-cold 0.1% (*v*/*v*) formic acid in water, then loaded onto preconditioned SPE cartridges (Strata-X 33 µm polymeric reversed phase; Phenomenex, Torrance, CA, USA). Cartridges were washed with 0.1% formic acid in water and 15% (*v*/*v*) ethanol in water to remove salts, then lipid mediators were eluted with methanol. Solvents were removed using an Eppendorf 5301 concentrator centrifugal evaporator (Eppendorf, Hauppauge, NY, USA), and dried extracts were stored at −80 °C until LC-MS/MS analysis.

### 2.13. LC-MS/MS Analysis of Lipid Mediators

All LC-MS/MS system components were from Shimadzu Scientific Instruments Inc. (Columbia, MD, USA). The LC system included four pumps (Pumps A/B: LC-30AD; Pumps C/D: LC-20AD XR), a SIL-30AC autosampler, and a CTO-30A column oven with a two-channel six-port switching valve. LC separation was performed on a RESTEK Ultra C8 column (150 × 2.1 mm, 3 µm; RESTEK Corporation, Bellefonte, PA, USA) with a Halo guard column (Optimize Technologies, Oregon City, OR, USA). MS/MS analysis was performed on a Shimadzu LCMS-8050 triple quadrupole mass spectrometer operated under both positive and negative electrospray ionization in multiple reaction monitoring (MRM) mode. MS/MS conditions and LC settings were optimized following our previously published methods [44,45]. Data acquisition and processing were performed using Shimadzu LabSolutions software (Version 5.135, copyright © 2008–2025 Shimadzu Corporation).

Before LC-MS/MS analysis, dried extracts were reconstituted in 50 µL methanol, and 10 µL was injected using the autosampler. The relative peak area of each lipid mediator was normalized to the corresponding internal standard and sample weight.

### 2.14. Lipidomic Analysis of Conditioned Medium

After siRNA treatment of primary myoblasts, conditioned medium (CM; ≥1 mL) was collected on differentiation day 3 and centrifuged at 350× *g* for 5 min at room temperature to remove cell debris. Supernatant (≥1 mL) was transferred to clean 1.5 mL low-retention microcentrifuge tubes and stored at −80 °C for lipidomic analysis using the same LC-MS/MS protocol described above.

### 2.15. Cholesterol and Triglyceride Measurement

In mice, blood samples were collected from mice 5 weeks after intravenous injection of MyoAAV-MHCK7-hMG29. Serum was isolated by centrifugation and stored at −80 °C until analysis. Serum total cholesterol levels were measured using a commercial assay kit (Cat. # MA-TC-1, RayBiotech, Peachtree Corners, GA, USA), and serum triglyceride levels were measured using a triglyceride assay kit (Cat. # MA-TG-1, RayBiotech, Peachtree Corners, GA, USA) according to the manufacturer’s instructions.

### 2.16. Ex Vivo Measurement of Maximal Contractile Force in EDL Muscle

Maximal isometric force of the mouse extensor digitorum longus (EDL) muscle was measured ex vivo using an intact muscle–tendon preparation mounted in a jacketed organ bath, as previously described in detail by the authors [46,47]. Briefly, mice were sacrificed by cervical dislocation, and the EDL muscle was removed for contractility analysis. Following mounting of muscles to the contractility system (Radinotti, Sarasota, FL, USA), contractile analysis was performed in physiological buffer (144 mM NaCl; 5 mM KCl; 1 mM MgCl_2_; 25 mM NaHCO_3_; 2.5 mM CaCl_2_; 10 mM glucose; pH 7.45) maintained at 37 °C and aerated with 95%/5% O_2_/CO_2_. EDL muscles were first allowed a 30 min equilibration period during which time they were contracted with maximal and submaximal-frequency stimulations with a three-minute rest interval (160/40 Hz for soleus; 200/100 Hz for EDL). Following equilibration, EDL muscles were stimulated to contract with frequencies ranging from 1 to 220 Hz to generate the force–frequency relationship. Next, the physiological buffer was replaced with a calcium-depleted physiological buffer (144 mM NaCl; 5 mM KCl; 1 mM MgCl_2_; 25 mM NaHCO_3_; 0 mM CaCl_2_; 0.1 mM EGTA; 10 mM glucose; pH 7.45), and EDL muscles were contracted with alternating maximal and submaximal-frequency stimulations with a three-minute rest interval for 30 min. The buffer was then replaced with a physiological buffer with normal calcium concentration, and muscles were allowed to recover under continued maximal and submaximal contractions for 30 min. To induce fatigue, the muscles were next contracted with alternating maximal and submaximal stimulations for 5 min, with a 2 s rest interval. Immediately following the fatiguing protocol, muscles were allowed a 30 min recovery period while contracting at maximal and submaximal force, with a 3 min rest period, followed by the addition of 5 mM caffeine to the muscle contractility chambers to evaluate calcium availability during the recovery period. At the end of the experiment, EDL muscle optimal length and muscle weight were measured, and muscles were snap frozen in liquid nitrogen and stored at −80 °C. A PowerLab/LabChart Software system (LabChart Pro version 6.1, ADInstruments, Colorado Springs, CO, USA) was used to store and analyze force data. In this study, muscle force is reported as absolute force (mN) because of its overall importance for activities of daily living (ADL) and likely more direct correlation to human loss of force in sarcopenia. The Expected Force due to Atrophy is simply the predicted maximal force if the only change were loss of muscle mass, assuming unchanged specific force. This was calculated based on the formula *F*_(expected, atrophy)_ = *F*_young_ × (Mass_group_/Mass_young_), where F_young_ is the mean maximal tetanic force (mN) of young WT EDL muscle, mass_young_ is the mean muscle mass of young WT EDL muscle, and Mass_aged or *Mg29*−/−_ muscle mass of the comparison group (aged WT or young *Mg29*^−/−^).

### 2.17. Statistical Analysis

All statistical analyses were performed using OriginPro software (version 10.2.0.196; OriginLab Corporation, Northampton, MA, USA). Data are presented as box plots or dot-scatter plots, with mean values indicated. For comparisons involving three or more groups, statistical significance was assessed using one-way analysis of variance (ANOVA), followed by Tukey’s honestly significant difference (HSD) post hoc test for pairwise comparisons. For comparisons between two groups, a two-tailed Student’s *t*-test was applied. Exact *p*-values were calculated within OriginPro and are reported in the figures or corresponding legends where applicable. Statistical significance is denoted as follows: *, *p* < 0.05, **, *p* < 0.01, and ***, *p* < 0.001. Prior to applying parametric tests (ANOVA and Student’s *t*-test), data normality was evaluated using the Shapiro–Wilk test. Homogeneity of variances was assessed using Levene’s test. In cases where assumptions were not violated, parametric tests were used as described. Sample sizes, animal numbers, and cell-culture replicates were performed with at least *n* = 3, while the exact sample size for each experiment is reported in the corresponding figure caption.

## 3. Results

### 3.1. Reduced Mg29/SYPL2 Expression Leads to Compromised Muscle Structure and Function

**Figure 1:**
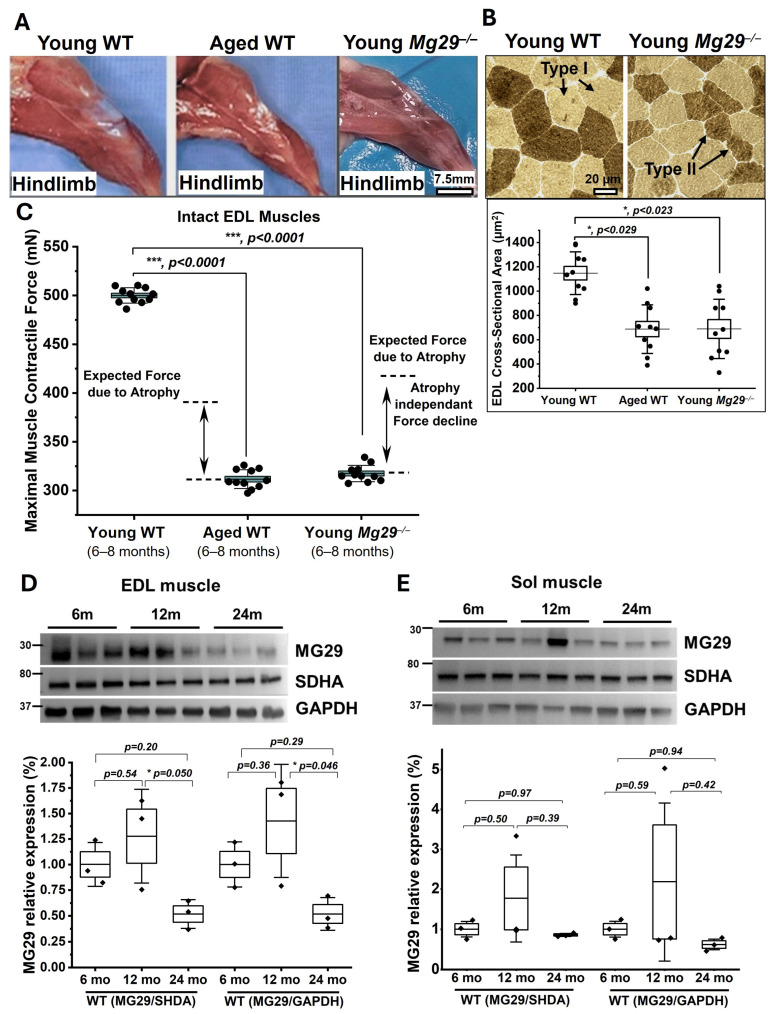
Experimental evidence of sarcopenia in skeletal muscles from aged WT and young *Mg29*^−/−^ mice and decreased expression of MG29 in aged skeletal muscle. (**A**) Pictures of the hind limb muscles from young WT mice (4-month-old) compared to aged WT (24-month-old) and young *Mg29*^−/−^ mice demonstrate that atrophy in aging is mirrored in young *Mg29*^−/−^ mice. (**B**) Cross-sections of EDL muscles from young WT and young *Mg29*^−/−^ mice show preferential atrophy of type II muscle fibers, recapitulating observations in humans. Arrows denote different fiber types as distinguished by enzyme histochemistry. (**C**) Normalized EDL muscle-specific force indicates that atrophy can only partially explain the decrease in force production. Data presented in boxplots where the interquartile range appears in the box with overlaid individual data points (circles.) (**D**,**E**) Western blots show MG29 protein levels in extracts of extensor digitorum longus (EDL) and soleus muscles from WT (C57Bl/6J) mice of various ages (indicated in months). GAPDH and succinate dehydrogenase (SDHA) levels were used to control protein content in each sample. MG29 protein levels were normalized to the corresponding SDHA and GAPDH levels to control for protein quantity in each sample. Data presented in boxplots where the interquartile range appears in the box with overlaid individual data points (circles or diamonds) and statistical significance levels were calculated by one-way ANOVA with Tukey post hoc analysis: *, *p* < 0.05; ***, *p* < 0.001. Sample size: *n* = 10–11 mice per age group were used for muscle force and fibro-type experiments; *n* = 3 mice per age group were used for western blot experiments.

We first tested whether loss of *Mg29* could serve as a model of accelerated sarcopenia by comparing muscle morphology and function in young *Mg29*^−/−^ mice, young wild-type (WT) mice, and old WT mice. Figure 1 presents gross anatomical morphology of all three experimental cases, which clearly shows that atrophy in aging is mirrored in young *Mg29*^−/−^ mice (Figure 1A). Cross-sections of EDL muscle from young WT and young *Mg29*^−/−^ mice show preferential atrophy of type II muscle fibers, recapitulating observations in humans [48] (Figure 1B). We conducted extensive quantification and determined that cross-sectional area (CSA) of EDL muscle type II fibers was significantly reduced in young *Mg29*^−/−^ mice compared with age-matched WT controls (1154 ± 180 µm^2^ in WT vs. 705 ± 250 µm^2^ in *Mg29*^−/−^*, p* ≤ 0.023, ANOVA/Tukey) and was similar to values observed in aged WT mice (685 ± 280 µm^2^), as shown in the box plot in Figure 1B. These data indicate that MG29 deficiency reproduces an atrophic phenotype characteristic of aged muscle despite a young chronological age. Consistent with reduced fiber size, *Mg29*^−/−^ mice showed impaired muscle contractile performance as shown in Figure 1C. Normalized EDL muscle-specific force indicates that atrophy can only partially explain the decrease in force production, indicating the disruption of EC coupling-related processes. To identify a direct mechanism, we measured the content of MG29 protein in skeletal muscles in WT mice at 6, 12, and 24 months, as shown in Figure 1D,E. As shown in the boxplots in Figure 1D, E, the quantification of MG29 protein contents indicates a biphasic profile with age. At 12 months, MG29 levels increased, followed by a drastic decrease at 24 months (by 40–50%). These increased levels at 12 months, perhaps, reflect enhanced muscle performance at this age, which aligns with previous observations by Hill et al. [49], confirming higher contractile force and power in isolated mouse muscles at 52 weeks. The drastic decrease in MG29 levels at 24 months coincides with the decrease in contractile function reported here. These data suggest a direct link between skeletal muscle-generated force capacity and MG29 levels.

### 3.2. MG29 Domain Structure

**Figure 2:**
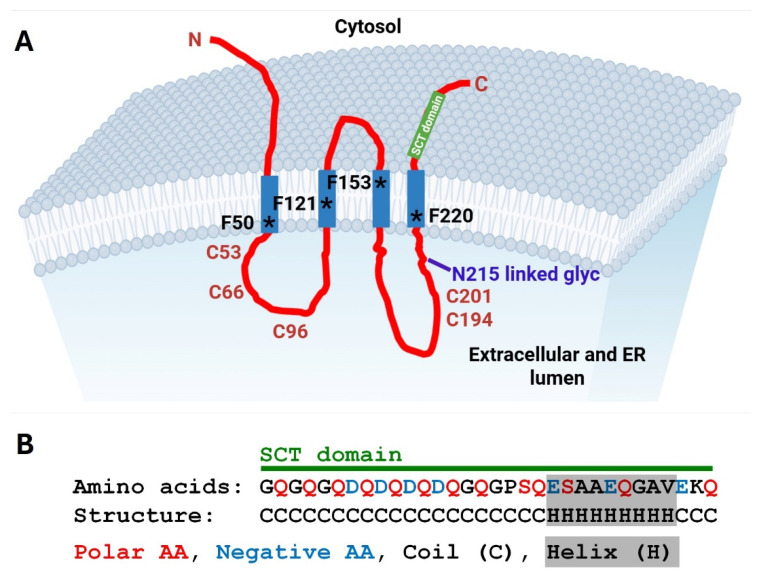
Domain structure of the MG29 protein. (**A**) MG29 protein has N- and C-terminal cytosolic domains on either side of the 4 transmembrane pass (blue) MARVEL domain, asterisks (*) indicate phenylalanine residues within transmembrane segments. Target-conserved phenylalanine (F) or cystidine (C) residues are shown with numbers to indicate position. The C-terminal SCT domain is shown in green. (**B**) Amino acid sequence of the SCT domain highlighting amino acids that are polar (red) or have negative charge (blue). Predicted secondary structure appears beneath the amino acids with a coil (C) and an α-helical region (H, gray shading).

To mechanistically study the function of MG29, we first analyzed its domain structure, as shown in Figure 2. The MG29 protein has N- and C-terminal cystolic domains on either side of the 4 transmembrane pass (blue) MARVEL domain (Figure 2A). Target-conserved phenylalanine (F) or cystidine (C) residues are shown with numbers to indicate position. The C-terminal SCT domain is shown in green. The amino-acid sequence of the SCT domain highlights amino acids that are polar (red) or have a negative charge (blue) as shown in Figure 2B. The predicted secondary structure appears beneath the amino acids with a coil (C) and an α-helical region (H, gray shading) as shown in Figure 2B. Notably, the C-terminal SCT domain contains a short, positively charged, helix-forming segment that is well suited to interact with negatively charged phospholipids and contribute to triad membrane organization, providing a structural basis for MG29-dependent regulation of SOCE and lipid composition.

### 3.3. Lipidomic Analysis Reveals That Cholesterol and Overall Fatty Acid Content Are Decreased in Mg29^−/−^ Muscle

**Figure 3:**
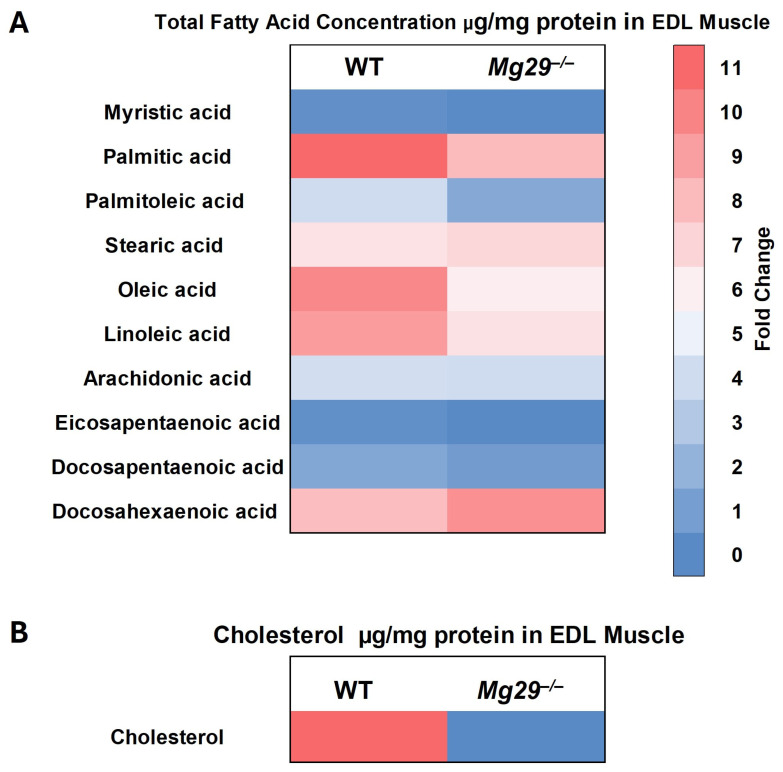
Lipidomics analysis of the muscles. Lipidomics analysis indicated decreased free cholesterol and many fatty acids in the *Mg29*^−/−^ EDL muscle. Total lipid extracts from young WT and *Mg29*^−/−^ were measured for levels of fatty acids using mass spectroscopy. (**A**) Several species of saturated fatty acids and some unsaturated species were greatly reduced in the *Mg29*^−/−^ muscle. (**B**) Total cholesterol was also drastically decreased in the *Mg29*^−/−^ muscle compared to the WT control. Data are presented as a heatmap with a color bar indicating the fold-change level. Sample size of *n* = 3 mice per group, with a total of 6 EDL muscles per group, was analyzed.

To explore mechanisms underlying the sarcopenia-like phenotype in *Mg29*^−/−^ mice, we examined the lipid composition of skeletal muscle. Targeted lipidomic analysis of EDL muscle revealed that total cholesterol and overall total fatty acids content were significantly reduced in *Mg29*^−/−^ muscle compared with age-matched WT controls, as shown in Figure 3. Several species of saturated fatty acids (including myristic, palmitic, and stearic acids) and some unsaturated species (such as oleic and linoleic acids) were greatly reduced in the *Mg29*^−/−^ muscle (Figure 3A), indicating a global depletion of key lipid species and broader changes in membrane lipid composition. Consistent with these changes, total free cholesterol levels were also substantially decreased in *Mg29*^−/−^ EDL muscle relative to WT, suggesting that loss of MG29 profoundly alters the lipid composition of skeletal muscle membranes (Figure 3B). Cholesterol is normally enriched in T-Tubules (TT), where it is required for the formation and maintenance of triad membrane structures and contractile function. Given that MG29/SYPL2 contains a MARVEL transmembrane domain with predicted cholesterol-binding properties, these findings suggest that MG29 contributes to the maintenance of TT cholesterol and fatty acid content, thereby supporting normal excitation–contraction coupling (EC coupling).

### 3.4. Altered Lipidomic Profiles in Mg29^−/−^ Gastrocnemius Muscle

We next performed targeted lipidomics to quantify bioactive lipid mediators derived from arachidonic acid (AA), linoleic acid (LA), eicosapentaenoic acid (EPA), docosahexaenoic acid (DHA), α-linolenic acid (ALA), and lysophosphatidylcholine (LPC) in gastrocnemius (GAS) muscle from young (13–16 weeks) and mid-aged (50–55 weeks) WT and *Mg29*^−/−^ mice. The slight 2–5 week differences in chronological age between WT and *Mg29*^−*/*−^ within each category fall within the same young adult or mid-aged range and are not expected to account for the genotype-dependent differences in lipid mediator profiles. These lipid mediators from the polyunsaturated fatty acid (PUFA) pathways regulate inflammation, oxidative stress, and muscle metabolism and have been implicated in age-related muscle remodeling [50,51].

In WT mice, 19 lipid mediators changed markedly between young and mid-aged mice, consistent with an age-related remodeling of muscle lipid signaling (Table 2). Strikingly, a similar pattern was observed in *Mg29*^−/−^ mice, and several key lipid mediators that increased in mid-aged WT muscles were already elevated in young *Mg29*^−/−^ animals. These included AA-derived 13,14-dihydro-15-keto-PGE_2_ (2.7-fold), 5-HETE (2.2-fold), and 5-KETE (1.9-fold), EPA (1.5-fold), DHA-derived 20-HDoHE (2.5-fold), and the ALA metabolite 9-HOTrE (2.4-fold) compared with young WT mice, as shown in Table 2 and Supplementary Figure S1. Thus, *Mg29*^−/−^ muscle exhibits a lipid mediator profile that resembles aged WT muscle, suggesting that MG29 loss accelerates age-like remodeling of lipid signaling pathways.

### 3.5. Altered MG29/SYPL2 Levels and Membrane Cholesterol Similarly Impair SOCE

**Figure 4:**
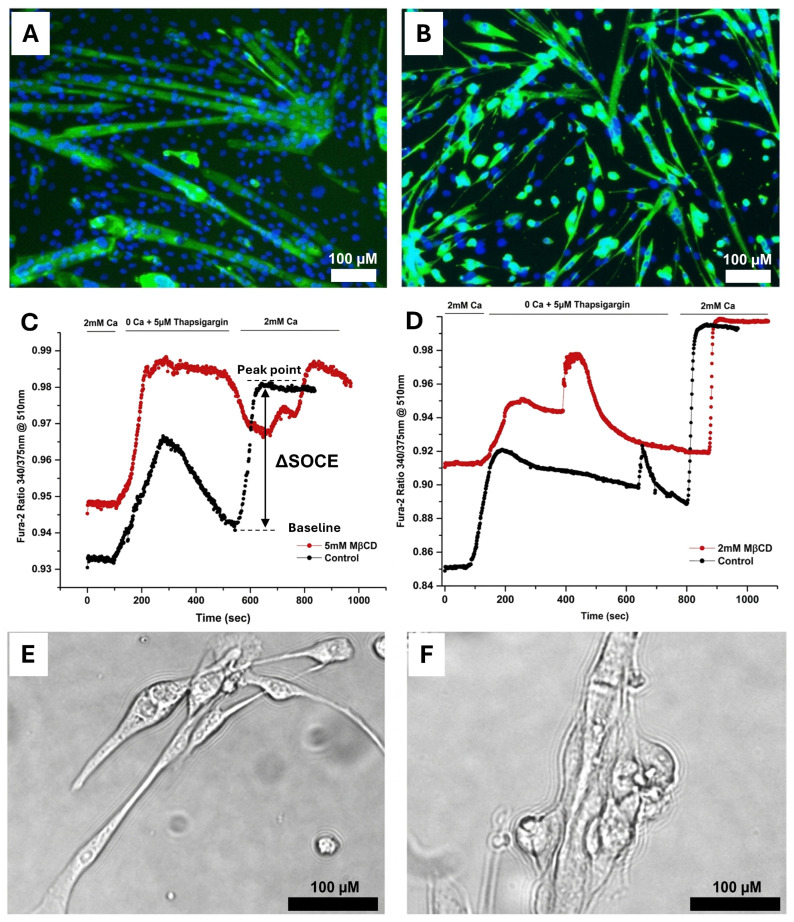
Effect of cholesterol on SOCE in myotubes. (**A**) Myosin IHC staining of C2C12 myotubes after 6 days of differentiation. (**B**) Myosin IHC staining of mouse primary myotubes after 3 days of differentiation. (**C**) Typical representative SOCE measurement curve of C2C12 myotubes treated with 5 mM MβCD. (**D**) Typical representative SOCE measurement by Fura-2/AM imaging on primary mouse myotubes (5-month-old WT mice) treated with 2 mM Methyl-β-Cyclodextrin (MβCD). (**E**) Bright-field microscopy of untreated primary myotubes. (**F**) Bright-field image of primary myotubes following 2 mM MβCD. A sample size of *n* = 14–17 myotubes, cultured in a glass-bottom Petri dish, was used for all SOCE experiments with C2C12 and mouse primary cells. Baseline and peak point lines are illustrated on panel C to indicate a sampling of how ΔSOCE was calculated.

Because lipidomics revealed reduced cholesterol in *Mg29*^−/−^ muscle, we asked whether direct cholesterol depletion would mimic the SOCE defects observed in aged and Mg29-deficient muscle. Partial extraction of membrane cholesterol by methyl-β-cyclodextrin (MβCD) significantly reduced SOCE in both C2C12 myotubes and primary myotubes from young WT mice (Figure 4). Immunohistochemical staining of C2C12 and mouse primary myotubes revealed successful myogenic differentiation with well-defined myosin-positive multinucleated cells after 6 days and 3 days of culture, respectively (Figure 4A,B). Treatment of C2C12 myotubes with 5 mM MβCD, for 30 min prior to the experiment, decreased SOCE by approximately 50% (61 ± 3 vs. 30 ± 5 delta [Ca^2+^]_i_ nM, respectively) as shown in Figure 4C, comparable to the reduction observed in aged WT and young *Mg29*^−/−^ muscle. To minimize potential membrane damage and better approximate physiological conditions, primary myotubes from 5-month-old WT mice were treated with 2 mM MβCD for 30 min prior to the experiment, which still reduced SOCE by 30–40% (64 ± 4 vs. 41 ± 5 delta [Ca^2+^]_i_ nM, respectively) as shown in Figure 4D. Bright-field microscopy of untreated primary myotubes demonstrated characteristic elongated, multinucleated morphology with well-organized cellular alignment (Figure 4E). Following treatment with 2 mM MβCD, primary myotubes displayed structural disruption with loss of typical elongated morphology and the appearance of irregular, fragmented cellular structures (Figure 4F). These morphological changes suggest that cholesterol depletion via MβCD treatment compromises myotube structural integrity in both primary and C2C12 myotube models. Further, these results strongly support a requirement for intact membrane cholesterol to maintain normal SOCE in skeletal muscle and indicate that *MG29*-dependent cholesterol regulation is functionally linked to Ca^2+^ entry.

### 3.6. Acute Mg29/SYPL2 Knockdown Recapitulates Chronic Mg29 Deficiency and Alters Ca^2+^ Homeostasis

To distinguish between developmental adaptations in *Mg29*^−/−^ mice and direct effects of *Mg29* loss, we acutely silenced *Mg29* in adult muscle using RNA interference. In FDB muscle electroporated with *Mg29* siRNA, MG29 protein levels were reduced, and SOCE activity significantly decreased compared with control muscles electroporated with scrambled siRNA, closely resembling the phenotype of young *Mg29*^−/−^ and aged WT muscles (Supplementary Figure S2).

**Figure 5:**
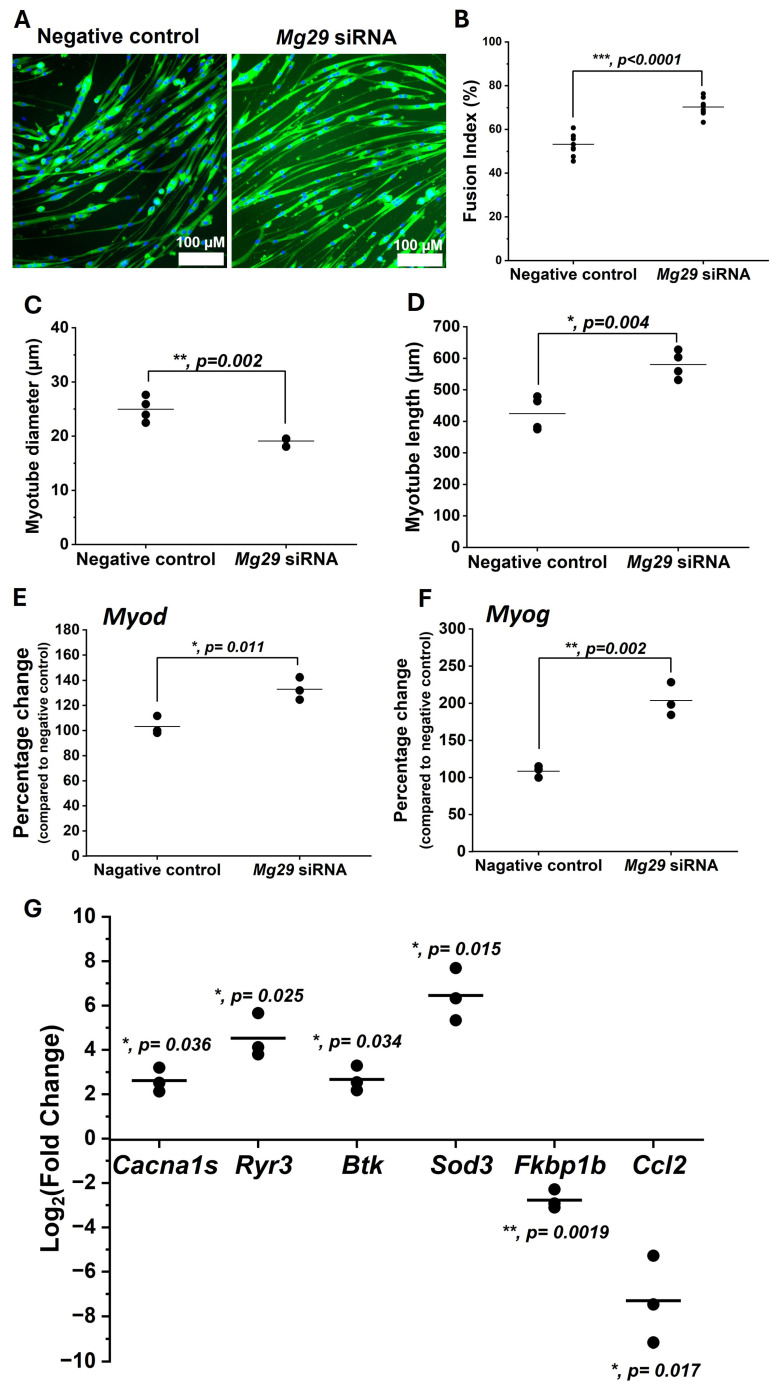
Acute knockdown of *Mg29* alters skeletal muscle cells differentiation and leads to cellular atrophy. (**A**) Representative Fluorescence images of DAPI and myosin heavy chain antibody-stained myocytes/myotubes of primary skeletal muscle cells at differentiation day 3 after *Mg29* siRNA treatment. (**B**) Summary data for fusion index (FI) for treatments of negative control and *Mg29* siRNA. (**C**,**D**) Reduced diameter and increased length in *Mg29* siRNA-treated myotubes. (**E**,**F**) Increased *Myod* and *Myog* expression in *Mg29* siRNA-treated myotubes. (**G**) Expression of genes involved in Ca^2+^ homeostasis (*Cacna1s*, *Ryr3*, *Btk*, *Fkbp1b*), oxidative stress (*Sod3*), and immunoregulatory and inflammatory processes (*Ccl2*) was altered (Presented as log_2_(FC) compared to the control). Data presented as scatter (dot) plots with a mean line, and statistical significance levels were calculated using Student’s *t*-test: *, *p* < 0.05; **, *p* < 0.01; ***, *p* < 0.001. Sample size was *n* = 3 per group in triplicate for the cell differentiation, function index, myotube length, and diameter calculations. For the RT-qPCR and gene arrays, a sample size of *n* = 3 was used.

Similarly, *Mg29* expression was reduced by approximately 80% in primary skeletal muscle cells 48 h after *Mg29* siRNA transfection. By day 3 of differentiation, *Mg29*-depleted cells formed longer, thinner myotubes compared with controls (Figure 5). Quantification results indicated a significantly decreased diameter (Figure 5C) and increased length (Figure 5D) (*p* < 0.05), while fusion index (Figure 5B) was paradoxically increased, in agreement with elevated expression of the myogenic markers *Myog* and *Myod (*Figure 5E,F). These findings indicate that *Mg29* is required for normal myotube morphology and suggest a role in coordinating differentiation with appropriate structural maturation.

To determine how MG29 loss impacts Ca^2+^ homeostasis and stress responses, we used a custom skeletal muscle-specific RT-qPCR array after *Mg29* siRNA treatment. Expression of *Cacna1s*, *Ryr3*, *Btk*, and *Sod3* increased 2.6 ± 0.48-, 4.5 ± 0.89-, 2.7 ± 0.51-, and 6.5 ± 1.06-fold, respectively, whereas *Fkbp1b* and *Ccl2* decreased 2.8 ± 0.38- and 7.3 ± 1.74-fold as shown in Figure 5G. These changes implicate MG29 in the regulation of Ca^2+^ channel complexes, oxidative stress defenses, and inflammatory signaling.

**Figure 6:**
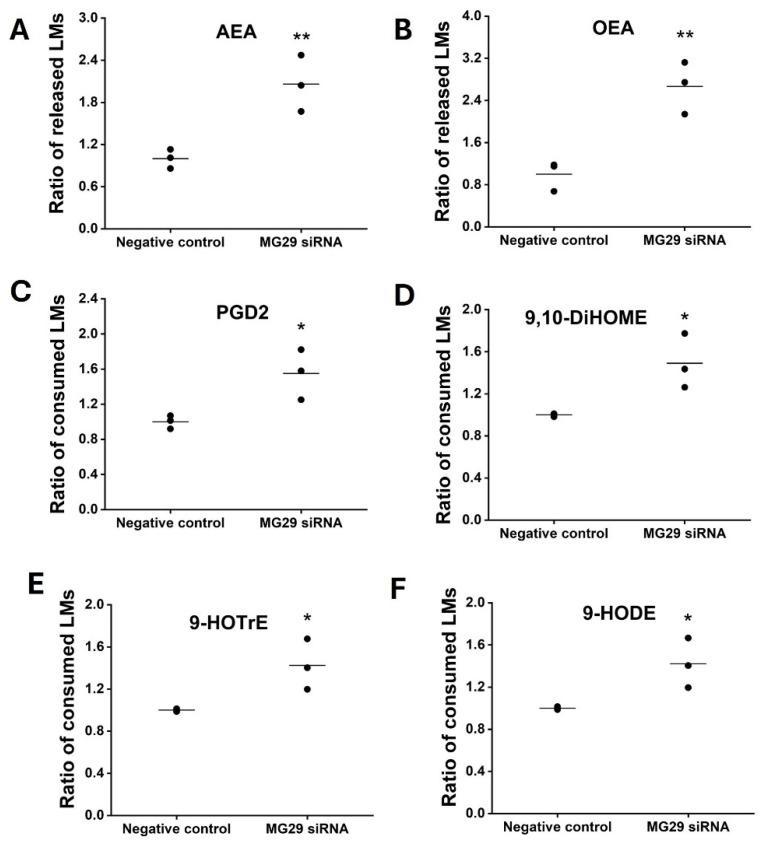
Quantification of lipid signaling mediators in media of negative control and *Mg29* siRNA-treated primary skeletal muscle myotubes at 72 h of differentiation. (**A**,**B**) Compared with the negative control, *Mg29* siRNA-treated cells showed an increased ratio of released lipid mediators in the cell-culture media (AEA and OEA). (**C**–**F**) Ratio of consumed lipid mediators by cells in the cell-culture media was also increased for PGD_2_, 9,10-DiHOME, 9-HOTrE, and 9-HODE. Data presented as scatter (dot) plots with a mean line, and statistical significance levels were calculated using Student’s *t*-test: *, *p* < 0.05; **, *p* < 0.01. A sample size of *n* = 3 per group was used for each group.

Further, lipidomic analysis of conditioned medium from *Mg29* siRNA-treated cells showed an increased release of the endocannabinoid-like mediators arachidonoylethanolamine (AEA) and oleoylethanolamide (OEA), and an increased consumption of PGD_2_, 9,10-DiHOME, 9-HOTrE, and 9-HODE (*p* < 0.05 vs. control) as shown in Figure 6. These mediators are derived from PUFAs and are known to influence inflammation, metabolism, and cell differentiation, suggesting that reduced MG29 expression modulates myogenic differentiation through complex changes in lipid signaling.

### 3.7. MG29/SYPL2 MARVEL Domain Mutations Uncouple Lipid Binding from SOCE Regulation

MG29 shares a conserved MARVEL transmembrane domain with other synaptophysin family proteins, which are known to bind cholesterol and oligomerize in membranes. Alignment of MG29 with synaptophysin (SYP) revealed several highly conserved residues in the four transmembrane spans. To dissect the contribution of these residues, we generated MG29 mutants targeting either putative cholesterol-binding residues or cysteines within the MARVEL domain.

In the MG29-4FA mutant, four conserved phenylalanine residues (one in each transmembrane span) were mutated to alanine, which altered MG29 localization relative to membrane cholesterol, as assessed by filipin staining in prior studies [52] (filipin is a fluorescent polyene probe that binds unesterified cholesterol and is commonly used to visualize membrane cholesterol distribution), and reduced co-localization with cholesterol-rich domains. This mutant also exhibited compromised SOCE, further linking MG29’s cholesterol association to its function in supporting Ca^2+^ entry. In contrast, the MG29-5C mutant, in which five cysteine residues were mutated to glycine, displayed normal localization with respect to filipin staining in prior studies but showed a marked reduction in SOCE. These data indicate that MARVEL-domain cysteines are essential for MG29-dependent regulation of SOCE but are not required for cholesterol binding, suggesting separable structural determinants for lipid association and Ca^2+^ signaling.

### 3.8. MG29 MARVEL Domain Mutations Differentially Impair SOCE in Skeletal Muscle Cells

**Figure 7:**
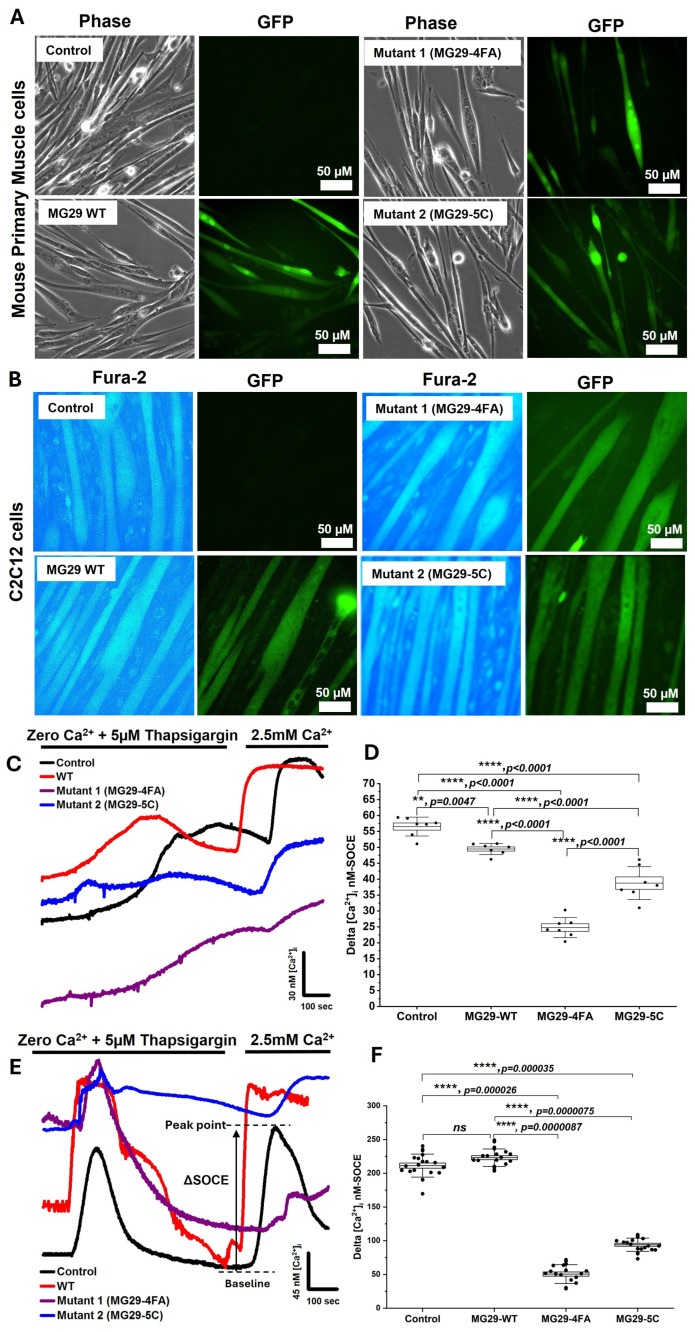
Effect of MG29 mutants on SOCE activity. (**A**) Phase contrast images of differentiated mouse primary muscle cells and GFP images showing transfection efficiency of the MG29 WT and mutant constructs. (**B**) Fura-2/AM loading and transfection efficiency of the MG29 WT and mutant constructs in C2C12 myotubes show that we can precisely match transfection with Ca imaging. (**C**) Representative Ca tracing illustrates the significant changes in SOCE response for both mutants tested vs. WT and control in C2C12 cells. (**D**) SOCE delta [Ca^2+^]_i_ nM in C2C12 myotubes. (**E**) Representative Ca tracing of SOCE response in primary myotubes from 5-month-old mice. (**F**) SOCE delta [Ca^2+^]_i_ nM in primary myotubes. In both C2C12 and primary myotubes, the mutants reduced both the peak and the integral response. Baseline and peak point lines are illustrated on panel E to indicate a sampling of how ΔSOCE was calculated. Data is from 8 to 15 cells for all groups. Data presented in boxplots and statistical significance levels were calculated by one-way ANOVA with Tukey post hoc analysis: ns, not significant (*p* > 0.05), **, *p* < 0.01 and ****, *p* < 0.0001.

To functionally validate the SOCE defects associated with MG29 MARVEL domain mutations, we examined Ca^2+^ entry dynamics in both C2C12 myotubes and primary mouse skeletal muscle cells transfected with GFP-tagged MG29 constructs. Phase-contrast and fluorescence microscopy confirmed successful transfection efficiency for MG29-WT and both mutant constructs (MG29-4FA and MG29-5C) in differentiated primary muscle cells and C2C12 myotubes (Figure 7A,B). Fura-2/AM Ca^2+^ imaging was used to assess SOCE following thapsigargin-induced SR Ca^2+^ store depletion.

Representative Ca^2+^ traces in C2C12 cells demonstrated that both MG29 mutants exhibited severely compromised SOCE compared to control and MG29-WT conditions (Figure 7C). Quantification of peak SOCE responses revealed that MG29-WT slightly decreased SOCE relative to control (49.2 ± 2.7 vs. 55.1 ± 3.4, *p* < 0.0047, delta [Ca^2+^]_i_ nM), while MG29-5C produced a dramatic reduction to 39.8 ± 3.6 and MG29-4FA showed the most severe impairment at 25.0 ± 2.5 (*p* < 0.0001 for both vs. control; Figure 7D). These data confirm that disruption of either cholesterol-binding residues (4FA) or MARVEL domain cysteines (5C) abolishes MG29’s ability to support robust SOCE.

To validate these findings in a more physiologically relevant system, we performed Ca^2+^ imaging in intact primary myotubes from 5-month-old mice (Figure 7E,F). Representative traces showed response patterns consistent with C2C12 data. Quantification of peak SOCE responses revealed that MG29-WT enhanced SOCE relative to control (221.3 ± 7.8 vs. 209.8 ± 12.4 delta [Ca^2+^]_i_ nM), while MG29-4FA produced the most dramatic reduction to 56.7 ± 9.2 and MG29-5C showed a severe impairment at 99.7 ± 8.6 (*p* < 0.000026 and *p* < 0.000035 vs control, respectively; Figure 7F). Notably, the integral response, which captures both the magnitude and duration of SOCE, was most severely compromised in the cholesterol-binding-deficient MG29-4FA mutant, supporting the hypothesis that MG29’s association with membrane lipids is essential for sustaining SOCE activity in skeletal muscle [31].

### 3.9. MG29 MARVEL Domain Mutations Differentially Regulate Cellular Lipid Composition

**Figure 8:**
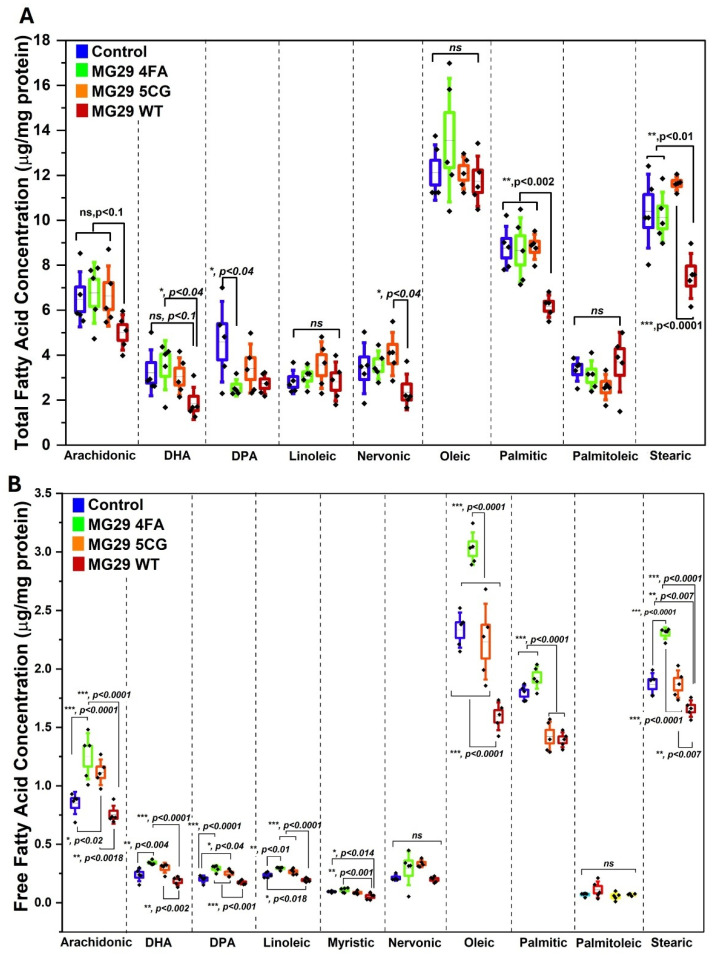
Total and free fatty acid profiles in C2C12 cells expressing MG29 variants. C2C12 myotubes were transfected with control vector (control), wild-type MG29 (MG29 WT), or MARVEL domain mutants (MG29 4FA, MG29 5CG). Total (**A**) and free (**B**) fatty acid concentrations were quantified by LC–MS/MS and normalized to protein content. Box-and-whisker plots show individual data points (black symbols), median (line), and interquartile range for each condition. In the total fatty acid pool (**A**), most species, including arachidonic acid, linoleic acid, oleic acid, and palmitoleic acid, were not significantly different among groups (ns), whereas MG29 WT showed selective reductions in total DHA and saturated fatty acids palmitate and stearate compared with control and/or MG29 mutants, with smaller genotype-specific changes in DPA and nervonic acid (*p*-values as indicated). In the free fatty acid pool (**B**), MG29 4FA and 5CG significantly increased free arachidonic acid relative to control and MG29 WT, both mutants elevated free DHA and DPA compared to MG29 WT, and MG29 4FA uniquely produced a robust increase in free oleic acid, with MG29 WT exhibiting the lowest oleic levels. Free palmitic and stearic acids were also differentially regulated (MG29 WT generally lower than control and mutants for palmitate, and MG29 4FA highest for stearate), whereas nervonic and palmitoleic acids did not differ significantly (ns). Statistical significance was assessed by one-way ANOVA with Tukey’s post hoc test: * *p* < 0.05; ** *p* < 0.01; *** *p* < 0.001. A sample size of *n* = 5 per group was used. Box colors indicate experimental groups: blue → Control; green → MG29 4FA; orange → MG29 5CG; and red → MG29 WT. For each lipid, results are presented in this order.

Having established that MG29 MARVEL domain mutations produce distinct SOCE phenotypes, we next investigated whether these functional defects correlate with alterations in cellular lipid metabolism. Lipidomic profiling of C2C12 skeletal muscle cells transfected with wild-type MG29 or MARVEL domain mutants (4FA and 5CG) revealed distinct fatty acid profiles across the MG29 variants, with both total and free fatty acid pools showing significant alterations (Figure 8).

In total fatty acid measurements (Figure 8A), most species showed limited changes among groups, with arachidonic acid and several unsaturated fatty acids (linoleic, oleic, and palmitoleic) remaining statistically comparable between all conditions (AA showing only trends with *p* ≈ 0.1 for MG29-WT versus control, 4FA, and 5CG). By contrast, MG29-WT displayed a modest but significant reduction in total DHA compared to the 4FA mutant (*p* = 0.04), and DPA was significantly decreased in 4FA relative to control (*p* = 0.04), indicating selective, rather than global, remodeling of long-chain omega-3 PUFAs. Nervonic acid was significantly lower in MG29-WT than in the SOCE-deficient 5CG mutant (*p* = 0.04), whereas saturated fatty acids showed a more pronounced effect: total palmitic acid was significantly reduced in MG29-WT compared to control, 4FA, and 5CG (*p* = 0.002), and total stearic acid was significantly lower in MG29-WT than in 5CG (*p* = 0.0001) and also decreased relative to control and 4FA (*p* = 0.01). Together, these data indicate that expression of wild-type MG29 selectively lowers total saturated (palmitic, stearic) and specific omega-3 fatty acids (DHA, DPA), whereas MARVEL-domain mutants and MG29 loss tend to preserve or modestly elevate these species rather than producing a uniform global change.

Analysis of free fatty acids (Figure 8B) demonstrated that free arachidonic acid was significantly elevated in both MG29-4FA and MG29-5CG compared to control (*p* = 0.0001 and *p* = 0.02, respectively), and both mutants also showed higher AA than MG29-WT (*p* < 0.001), indicating that disruption of MG29’s functional domains drives accumulation of this omega-6 PUFA in the free fatty acid pool. Free DHA was significantly increased in MG29-4FA relative to control (*p* = 0.004) and MG29-WT (*p* < 0.0001), and MG29-5CG also differed from MG29-WT (*p* < 0.002), showing that both mutants perturb omega-3 free fatty acid homeostasis, with the largest effect in 4FA. Similarly, free DPA was markedly elevated in MG29-4FA and MG29-5CG: control differed significantly from 4FA (*p* < 0.0001) and 5CG (*p* < 0.04), and MG29-WT was significantly lower than both mutants (*p* < 0.001), reinforcing the idea that MARVEL-domain disruption promotes accumulation of long-chain PUFAs. Free linoleic acid was significantly higher in MG29-4FA and MG29-WT relative to control (*p* < 0.01), and MG29-WT differed significantly from both 4FA and 5CG (*p* < 0.0001), indicating genotype-specific remodeling of omega-6 precursors rather than a uniform increase in all mutants. Free myristic acid was significantly reduced in MG29-WT compared to control and 4FA (*p* < 0.01), whereas nervonic and palmitoleic acids did not differ significantly among conditions (ns), highlighting a selective rather than global reshaping of free saturated/monounsaturated pools. Free oleic acid showed the most striking phenotype: MG29-4FA exhibited significantly higher levels than control, MG29-WT, and 5CG (*p* < 0.0001), while MG29-WT was significantly lower than all other groups (*p* < 0.0001), indicating that loss of cholesterol-binding residues strongly enhances monounsaturated free fatty acid accumulation. Free palmitic acid was also significantly altered, with MG29-WT reduced relative to control and 4FA, and 5CG elevated compared to control and 4FA (all *p* = 0.0001), whereas free stearic acid displayed a complex pattern in which 4FA and WT were significantly higher than control (*p* < 0.0001 and *p* < 0.007, respectively), 4FA exceeded both WT and 5CG (*p* < 0.0001), and WT was higher than 5CG (*p* < 0.007), consistent with differential regulation of saturated free fatty acids among MG29 variants.

These findings demonstrate that MG29 MARVEL domain mutations produce distinct lipid signatures that parallel their SOCE phenotypes: both 4FA and 5CG significantly increase free arachidonic acid and several long-chain PUFAs (DHA, DPA), while wild-type MG29 favors lower levels of saturated and monounsaturated free fatty acids (myristic, palmitic, stearic, oleic) under these conditions, and the cholesterol-binding-deficient 4FA mutant uniquely drives a robust elevation of free oleic acid. The selective enrichment of omega-6 PUFAs and reorganization of saturated and omega-3 species in dysfunctional MG29 variants suggest that proper MG29 function is required to maintain a balanced combination of omega-6, omega-3, and saturated fatty acids in skeletal muscle rather than a simple uniform reduction in any single class.

To determine whether MG29 protein-mediated alterations in cellular lipid metabolism translate to systemic effects, we examined serum lipid profiles in mice receiving intravenous injection of MyoAAV-MHCK7-MG29 under high-fat diet (HFD) conditions. Five weeks post-injection, serum total cholesterol levels showed a trend toward reduction in MG29-treated mice (305 ± 60 mg/dL) compared to controls (478 ± 70 mg/dL), though this difference did not reach statistical significance (Supplementary Figure S3A). Similarly, serum triglyceride levels remained comparable between control (67.5 ± 11.5 mg/dL) and MG29-treated mice (70.0 ± 11.5 mg/dL, Supplementary Figure S3B). These data indicate that while MG29 profoundly influences intracellular lipid composition and fatty acid profiles in skeletal muscle cells (Figure 8), its overexpression does not significantly alter circulating lipid levels under HFD challenge, suggesting that MG29’s lipid regulatory functions are primarily local to muscle tissue rather than systemic metabolic regulators.

## 4. Discussion

Our results demonstrate that reduced MG29/SYPL2 expression reproduces key structural, functional, and lipid signaling features of aging skeletal muscle, identifying MG29 as a central regulator of Ca^2+^ homeostasis and membrane organization in sarcopenia. Young *Mg29*^−/−^ mice exhibit reduced muscle fiber CSA, impaired specific force, shortened lifespan, and blunted SOCE, closely mirroring phenotypes observed in aged WT muscle. Consistent with this interpretation, compromised SOCE is directly demonstrated by our Fura-2/AM measurements in cholesterol-depleted myotubes (Section 3.5, Figure 4), *Mg29*-deficient muscle (Section 3.6), and MG29 MARVEL domain mutants in both C2C12 and primary myotubes (Section 3.8, Figure 7). These findings extend prior work [31] linking MG29 to defective SOCE in aged muscle and strengthen the concept that age-related MG29 loss contributes causally to dynapenia rather than simply reflecting a downstream consequence of aging.

To identify a direct link between MG29 and age-related muscle weakness, we quantified MG29 protein levels in WT muscles at 6, 12, and 24 months. These measurements revealed a biphasic profile with a compensatory increase at 12 months followed by a ~40–50% reduction at 24 months. This pattern parallels the non-linear trajectory of contractile performance, as Hill et al. [49] reported enhanced force and power in isolated mouse muscles at ~52 weeks before a subsequent decline at older ages, consistent with a mid-life functional peak. We propose that elevated MG29 at 12 months supports this transient optimization of muscle performance, whereas the later drop in MG29, together with additional aging mechanisms, contributes to the pronounced loss of muscle quality and specific force in old WT muscle. Although the difference between 6- and 24-month MG29 levels did not reach statistical significance in this small cohort, the mean value at 24 months was lower and more variable, which is typical of late-life muscle and likely reflects true biological heterogeneity rather than preserved MG29 expression.

A major new insight from this study is that MG29/SYPL2 modulates muscle lipid composition and signaling, particularly cholesterol and PUFA-derived mediators, and that these changes are tightly coupled to SOCE function. Lipidomic analyses revealed decreased cholesterol and global free fatty acid content in *Mg29*^−/−^ muscle, together with an “aged” pattern of lipid mediator levels in young *Mg29*^−/−^ gastrocnemius that closely resembled those of mid-aged WT mice. Several AA-, EPA-, DHA-, and ALA-derived mediators that increased with aging in WT muscle were already elevated in young *Mg29*^−/−^ mice, indicating that MG29 loss accelerates age-like remodeling of lipid signaling pathways. Because cholesterol is critical for TT and triad structure, and lipid mediators regulate inflammation and metabolism, MG29 appears to integrate structural and signaling aspects of membrane biology that are central to muscle aging.

Our functional experiments support a mechanistic link between MG29-dependent cholesterol homeostasis and SOCE. Partial extraction of membrane cholesterol with MβCD in C2C12 and primary myotubes reduced SOCE by 30–50%, reproducing the magnitude of SOCE impairment seen in aged WT and *Mg29*^−/−^ muscles. These observations align with studies in non-excitable cells where cholesterol-rich microdomains and caveolae concentrate STIM and Orai proteins to facilitate SOCE activation [53,54]. In skeletal muscle, MG29 localizes to TT and SR membranes and forms oligomers, suggesting that it could stabilize cholesterol-enriched triad microdomains that support efficient coupling between STIM, Orai, and RyR1. Consistent with this model, our analysis of the C-terminal SCT domain (Figure 2B) revealed a positively charged, helical segment positioned at the luminal side of the membrane, which is compatible with interactions with acidic phospholipids and supports a role for MG29 in organizing cholesterol- and lipid-rich triad microdomains that sustain SOCE.

The MG29 mutant analyses further refined this model by showing that distinct MARVEL-domain residues differentially control lipid association and SOCE regulation. The MG29-4FA mutant disrupted co-localization with filipin-stained cholesterol and reduced SOCE, indicating that conserved transmembrane phenylalanine is most likely important for lipid interactions and proper positioning of MG29 within TT membranes, although additional studies will be needed to define specific cholesterol-binding sites. In contrast, the MG29-5C mutant retained normal cholesterol co-localization but still showed markedly impaired SOCE, implying that cysteine residues are essential for MG29’s signaling function—possibly by supporting conformational changes, oligomerization, or interactions with SOCE-associated proteins. Together, these findings suggest that MG29 may fulfill at least two separable roles at the triad, contributing to cholesterol- and lipid-rich membrane architecture and modulating SOCE through protein–protein interactions, although the precise molecular mechanisms remain to be determined. The dissociation between cholesterol binding (retained in MG29-5C) and SOCE function (lost in MG29-5C) indicates that MG29 may act as a molecular scaffold that coordinates both lipid organization and Ca^2+^ channel assembly at triad junctions. This dual functionality positions MG29 as a critical integrator of membrane structure and excitation–contraction coupling, whose loss during aging disrupts both aspects simultaneously. Future structural and in silico modeling studies of MG29’s MARVEL domain will be important to test these predictions.

Acute knockdown experiments in adult FDB muscle and primary myotubes demonstrate that MG29 is required for normal SOCE and muscle cell morphology independently of developmental compensation. In vivo electroporation of MG29 siRNA decreased SOCE in adult fibers, confirming that MG29 modulates Ca^2+^ entry in mature muscle. In primary myotubes, acute MG29 depletion produced longer, thinner fibers with increased fusion index and elevated myogenic markers, indicating that differentiation proceeds but structural maturation is abnormal. This phenotype suggests that MG29 coordinates membrane growth, triad formation, and Ca^2+^ signaling during late stages of myogenesis, and that its loss drives a maladaptive remodeling reminiscent of aged muscle.

MG29 knockdown also altered the expression of genes involved in Ca^2+^ handling (e.g., *Cacna1s*, *RyR3*, *Fkbp1b*), oxidative stress (*Sod3*), and inflammation (*Ccl2*), pointing to broader regulatory roles in muscle homeostasis. Increased *Cacna1s* and *RyR3* levels could represent compensatory upregulation of Ca^2+^ channels in response to reduced SOCE, while decreased *Fkbp1b* may impair RyR stabilization and contribute to Ca^2+^ leak and fatigue. Upregulation of *Sod3* and downregulation of *Ccl2* suggest that MG29 influences redox and inflammatory status, which are key determinants of sarcopenia progression. The associated changes in AEA, OEA, PGD_2_, DiHOME, HOTrE, and HODE flux in the culture medium further demonstrate that MG29 modulates PUFA-derived lipid mediator networks that regulate metabolism, inflammation, and differentiation. The elevation of arachidonic acid-derived mediators in MG29-deficient cells, coupled with altered endocannabinoid-like lipid release, suggests that MG29 loss shifts muscle toward a pro-inflammatory, catabolic state characteristic of sarcopenia. This lipid-mediated inflammation may contribute to the reduced specific force observed in *Mg29*^−/−^ muscle beyond what can be explained by fiber atrophy alone, in parallel with oxidative stress and lipid peroxidation processes that are well established in aging muscle.

Collectively, our data support a working model in which age-related loss of MG29/SYPL2 disrupts triad cholesterol content and membrane organization, leading to compromised SOCE, altered Ca^2+^ homeostasis, and maladaptive lipid signaling (including selective increases in free AA, DHA/DPA, and oleic acid in MG29 mutants) that together drive atrophy-independent declines in muscle-specific force. Moreover, MG29 is only one component of a broader excitation–contraction coupling network: aging-related weakness arises from combined atrophy-dependent and atrophy-independent mechanisms (including impaired SOCE, altered Ryr1 function, mitochondrial dysfunction, and lipid signaling changes), so the magnitude of force loss is not expected to scale linearly with MG29 abundance. Our prior work [31,33] shows that partial or complete MG29 loss in otherwise young muscle is sufficient to blunt SOCE and reduce specific force, indicating that once MG29 falls below a functional threshold—as in 24-month muscle—even modest additional changes in its level can have disproportionately large effects on Ca^2+^ handling and muscle quality. This model is consistent with prior findings that STIM1 and Orai1 expression are not markedly reduced in aged muscle, suggesting that upstream structural regulators such as MG29 are critical determinants of SOCE impairment [39,55]. This study suggests that MG29 may be a contributing factor in preserving muscle quality during aging and could represent a potential therapeutic target, although this possibility requires further validation. It also raises the possibility that MG29-related mechanisms might be relevant in other MG29-associated conditions, such as obesity, depression, kidney dysfunction, and neurodegeneration, but these extensions remain speculative and should be interpreted with caution.

## 5. Clinical and Translational Implications

Our findings have several important translational implications for sarcopenia intervention strategies. First, the tissue-specific nature of MG29’s lipid regulatory function (as suggested by the lack of detectable systemic lipid changes in Supplementary Figure S2) raises the possibility that MG29-targeted interventions might modulate muscle metabolism with limited systemic metabolic effects, although this remains to be confirmed.

Second, the separable cholesterol-binding and SOCE-regulatory domains of MG29 identified through our mutant analyses may enable the development of small molecules or peptides that selectively enhance one function over the other, potentially allowing fine-tuning of therapeutic interventions. Third, the accelerated sarcopenia phenotype in young *Mg29*^−/−^ mice suggests that MG29 protein levels or activity could serve as biomarkers for sarcopenia risk assessment in middle-aged adults, enabling earlier intervention before substantial muscle loss occurs.

The observation that multiple PUFA-derived lipid mediators exhibit an “aged” profile in young *Mg29*^−/−^ muscle raises the possibility that dietary omega-3 supplementation or specialized pro-resolving mediator (SPM) therapy might partially compensate for MG29 deficiency. However, our data showing that DHA levels remain low across all conditions (Figure 8B) suggest that substrate availability alone may not be sufficient if MG29-dependent membrane organization is required for efficient lipid mediator biosynthesis. This highlights the need for combination approaches that address both lipid supply and membrane structural integrity in aging muscle.

## 6. Study Limitations and Future Directions

Future work should define how MG29 interacts with specific SOCE components (STIM1, Orai1/2/3, TRPC channels) and triad proteins such as RyR1 and junctophilins, and whether restoring MG29 expression or modulating its lipid-binding domains can rescue SOCE and muscle function in aged muscle. In addition, dissecting the causal contribution of individual lipid mediators and cholesterol-dependent microdomains to MG29’s effects may reveal new pharmacological strategies aimed at stabilizing triad architecture and Ca^2+^ signaling in sarcopenic muscle.

A few limitations of the present study warrant consideration. First, while our in vitro cholesterol depletion experiments with MβCD establish a functional link between cholesterol and SOCE, MβCD may have off-target effects on membrane proteins beyond cholesterol extraction. Future studies using more selective cholesterol manipulation approaches (e.g., cholesterol oxidase, statin treatment, or genetic modulation of cholesterol biosynthesis enzymes) would strengthen these conclusions. Second, our lipidomic analyses provide correlative evidence for altered lipid signaling in *Mg29*^−/−^ muscle, but causality remains to be established through gain-of-function and loss-of-function experiments targeting specific lipid mediator pathways. Third, the *Mg29*^−/−^ model represents a complete genetic knockout, whereas aging involves gradual MG29 decline; future studies should examine whether partial MG29 reduction (e.g., through heterozygous mice or graded siRNA approaches) more faithfully recapitulates the aging trajectory.

Additionally, sex-specific differences in MG29 expression and function were not examined in the present study. Given that sarcopenia prevalence and progression differ between males and females, and that sex hormones influence both lipid metabolism and Ca^2+^ homeostasis, future investigations should address whether MG29’s protective effects on muscle quality are sexually dimorphic. Finally, while our mutant analyses dissociate cholesterol binding from SOCE regulation, the precise molecular mechanisms by which MARVEL-domain cysteines support SOCE remain unclear and may involve disulfide bond formation, palmitoylation, or direct protein–protein interactions with STIM1 or Orai channels.

Long-term studies are needed to determine whether pharmacological or gene therapy-based restoration of MG29 in aged muscle can reverse established sarcopenia or merely prevent further decline. The recent development of muscle-specific AAV vectors (as demonstrated in our Supplementary Figure S3 experiments) provides a promising platform for such translational studies. Furthermore, identifying the upstream regulators of MG29 expression during aging—whether transcriptional repressors, microRNAs, or protein degradation pathways—could reveal additional therapeutic entry points upstream of MG29 itself. Finally, given that MG29 expression correlates with metabolic and neuropsychiatric conditions beyond sarcopenia, multi-system investigations may uncover broader roles for this protein in organismal aging and healthspan. Future lipidomic work will also need to quantify oxidized phospholipids and specific oxysterols to define how oxidative lipid damage intersects with the MG29–cholesterol–SOCE axis in aging muscle.

## 7. Conclusions

This study identifies MG29/SYPL2 MG29; as a key organizer of triad membrane cholesterol and lipid signaling required for normal SOCE during aging. Young *Mg29*^−/−^ mice recapitulate key features of aged muscle, including fiber atrophy, reduced specific force, blunted SOCE, and accelerated remodeling of PUFA-derived lipid mediator profiles. Our findings demonstrate that MG29 maintains triad membrane cholesterol content required for normal SOCE, and that its MARVEL domain contains separable structural determinants for cholesterol binding and Ca^2+^ channel regulation. Acute MG29 knockdown in adult muscle and primary myotubes impairs SOCE, disrupts myotube morphology, and alters the expression of genes governing Ca^2+^ handling, oxidative stress, and inflammation, while simultaneously shifting lipid mediator networks toward pro-inflammatory and catabolic states. Together, these results establish MG29/SYPL2 as a molecular link between membrane organization and excitation–contraction coupling whose preservation may represent a therapeutic strategy for maintaining muscle quality during aging.

## Figures and Tables

**Table 1:** RT-qPCR primers used in this study.

Primer	Primer Sequence (5′ to 3′)
*Gapdh F*	TGCGATGGGTGTGAACCACGAGAA
*Gapdh R*	GAGCCCTTCCACAATGCCAAAGTT
*MyoD F*	CCCCGGCGGCAGAATGGCTACG
*MyoD R*	GGTCTGGGTTCCCTGTTCTGTGT
*MyoG F*	TGAGCATTGTCCAGGCCAG
*MyoG R*	GCTTCTCCCTCAGTGTGGCT

**Table 2:** Relative concentration of LMs in WT and *Mg29*^−/−^ mice at different ages (% to Young-WT).

Metabolic Pathways	LMs	WT				*Mg29* ^−/−^			
		Young		Mid-Aged		Young		Mid-Aged	
		Mean	SD	Mean	SD	Mean	SD	Mean	SD
Arachidonic acid (AA), n-6 PUFA	20-hydroxy-PGF2a	100.0	38.7	310.9 ***	23.0	71.1 ^bbb^	16.2	754.8 ***^, aaa, bbb, ccc^	45.3
	6-keto-PGF1a	100.0	9.0	33.7 **	17.8	101.1 ^bb^	37.5	46.1 *^, a, c^	17.7
	8-iso-PGE2	100.0	42.9	180.0	94.7	115.1	34.1	341.2 ***^, aaa, bb^	39.5
	13,14-dihydro-15-keto-PGE2	100.0	23.2	129.5	46.1	267.9 ^aaa, bb^	55.9	170.5 *	30.6
	8-HETE	100.0	52.6	162.6	53.1	119.5	60.0	289.2 *^, aa^	85.3
	5-HETE	100.0	43.8	363.5	233.4	216.4	148.1	700.1 *^, aa^	286.5
	5-KETE	100.0	34.7	282.2	92.6	192.6	168.2	617.1 **^, aa, b^	192.6
	AA	100.0	34.7	35.5	24.6	143.5	32.1	45.8 **	30.9
Linoleic acid (LA), n-6 PUFA	13-KODE	100.0	14.7	183.7	47.1	116.6	39.3	482.1 *^, aa, b^	256.5
Eicosapentaenoic acid (EPA), n-3 PUFA	17,18-DiHETE	100.0	81.8	490.8 **	176.4	66.4 ^bb^	22.2	335.7	174.1
	EPA	100.0	43.5	214.0 **	48.9	147.7	2.5	100.7 ^bb^	21.8
Docosahexaenoic acid (DHA), n-3 PUFA	20-HDoHE	100.0	22.8	413.2 *	322.2	252.3	157.4	880.2 **^, aaa^	215.1
	16-HDoHE	100.0	38.6	871.3 ***	165.8	252.4 ^bb^	152.5	1325.2 ***^, aaa, b^	270.2
	13-HDoHE	100.0	39.7	674.5 ***	263.8	222.8 ^bb^	127.3	733.0 **^, aaa^	49.5
	10-HDoHE	100.0	60.6	393.5 *	154.2	142.0 ^b^	82.8	437.9 *^, aa^	121.4
	4-HDoHE	100.0	30.5	945.0 *	614.4	225.3	180.4	1192.4 *^, aa^	338.8
	DHA	100.0	29.1	53.5	14.1	188.7 ^aa, bbb^	41.4	64.9 ***	1.8
α-linolenic acid (ALA), n-3 PUFA	9-HOTrE	100.0	112.6	806.8 *	266.9	236.4	84.0	722.7 ^a^	487.1
Lysophosphatidylcholine (lysoPC)	Lyso-PAF	100.0	10.1	31.3 ***	6.0	98.2 ^bbb^	13.7	37.7 ***^, aaa^	6.7

Notes: One-way ANOVA with post hoc Tukey test (α =0.05), *n* = 4. * *p* ≤ 0.05, ** *p* ≤ 0.01, and *** *p* ≤ 0.001 as compared with the Young in each group. ^a^ *p* ≤ 0.05, ^aa^ *p* ≤ 0.01, and ^aaa^ *p* ≤ 0.001 as compared with WT-Young. ^b^ *p* ≤ 0.05, ^bb^ *p* ≤ 0.01, and ^bbb^ *p* ≤ 0.001 as compared with WT-Mid-aged. ^c^ *p* ≤ 0.05, and ^ccc^ *p* ≤ 0.001 as compared with Young *Mg29*^−/−^.

## Data Availability

The original contributions presented in this study are included in the article/Supplementary Materials. Further inquiries can be directed to the corresponding authors.

## Supplementary Materials

The following supporting information can be downloaded at https://www.mdpi.com/article/10.3390/biom16070988/s1: Figure S1. Percentage concentrations of lipid mediators in gastrocnemius muscles from WT and *Mg29*^−/−^ mice at different ages; Figure S2. MG29 knockdown impairs store-operated calcium entry in intact muscle fibers; Figure S3. Effects of MG29 overexpression on serum lipid levels in HFD mice; and all western blotting raw data with their quantification analysis.
