# Supplementary material for: Reduced Synaptophysin-like 2 (MG29/SYPL2) Levels Mimic Age-Related Alterations in Skeletal Muscle Calcium Homeostasis and Lipid Signaling
Source: Biomolecules. 2026 Jul 4;16(7):988. doi: 10.3390/biom16070988 (PMC13406257; doi:10.3390/biom16070988)
Supplement: Supplementary file 1 [file biomolecules-16-00988-s001.zip › biomolecules-4353779-supplementary.pdf]

Reduced Synaptophysin-like 2 (MG29/SYPL2) Levels Mimic Age-related Alterations in Skeletal Muscle Calcium Homeostasis and Lipid Signaling.

Kamal Awad[1,#], Jian Huang[1,#], Marian N. Aziz[2], Zhiying Wang[3], Leticia Brotto[1], Kyung Eun Lee[4], Jongsoo Kim[4], Karthikraj Rajendiran[1], Liubov V. Gushchina[5], Noah Weisleder[6, *], Marco Brotto[1, *]

Supplementary Materials

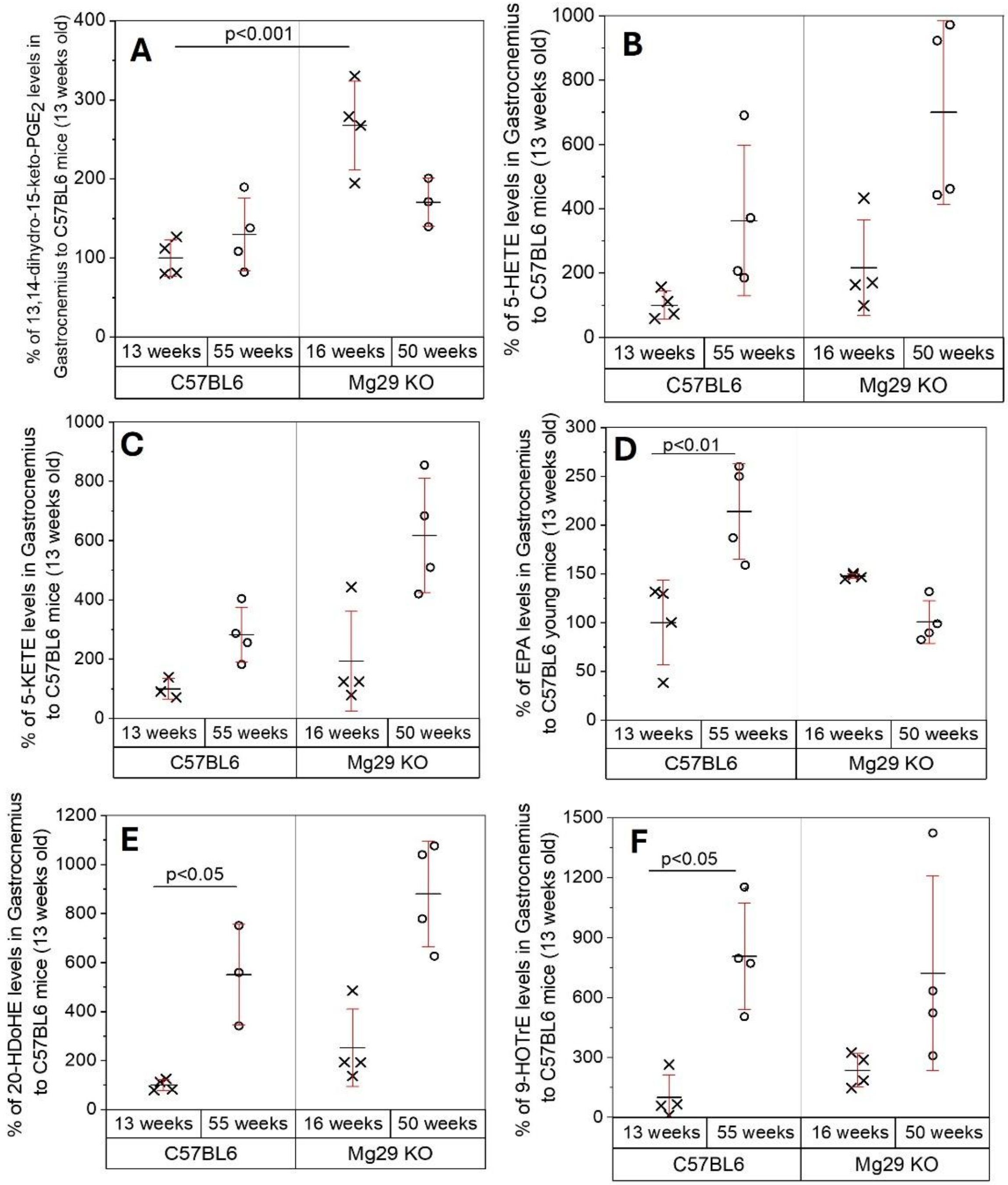

Figure S1. Percentage concentrations of lipid mediators in gastrocnemius muscles from WT and *Mg29$^{-/-}$* mice at different ages. (A) 13,14-dihydro-15-keto-PGE2, (B) 5-HETE, (C) 5-KETE, (D) EPA, (E) 20-HDoHE, (F) 9-HOTrE. Mean ±SD, n=4. The levels of specific lipid signaling mediators increase with aging in WT mice (young vs. mid-aged). A remarkably similar pattern was observed in young *Mg29$^{-/-}$* mice and middle-aged WT mice.

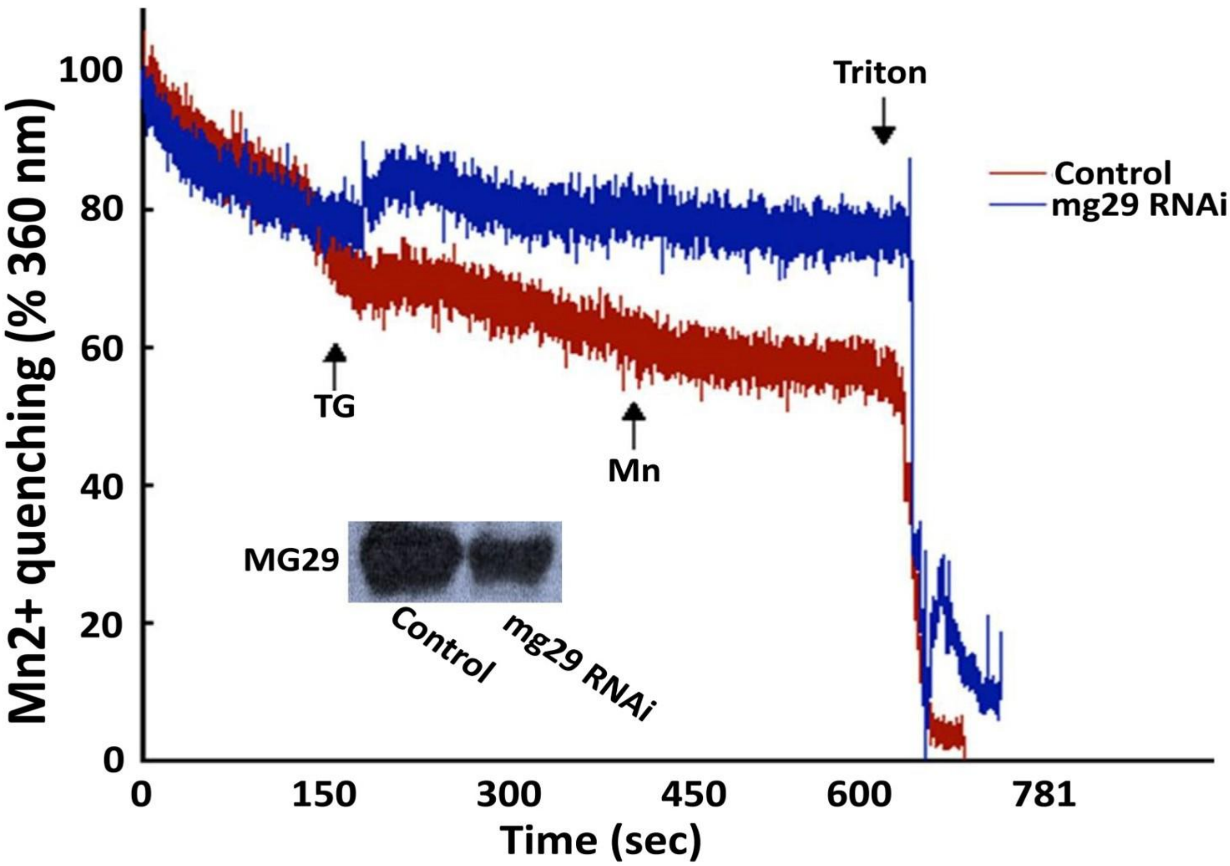

Figure S2: MG29 knockdown impairs store-operated calcium entry in intact muscle fibers. Intact FDB muscle fibers were transfected with scrambled siRNA (Red trace-Control) or *Mg29*-siRNA (Blue trace) for 28 days. SOCE function was assessed using Mn^{2+} quenching of Fura-2 fluorescence at 360 nm. Thapsigargin (TG) was added to deplete intracellular calcium stores, followed by Mn^{2+} addition to monitor divalent cation entry through store-operated calcium channels. Triton X-100 was added at the end to fully quench fluorescence. MG29 silencing (confirmed by western blot, inset) significantly reduced the rate of Mn^{2+}-induced fluorescence quenching compared to scrambled siRNA controls, indicating decreased SOCE activity (n=5). The slower decay in MG29-deficient fibers demonstrates that MG29 is required for normal SOCE function in skeletal muscle.

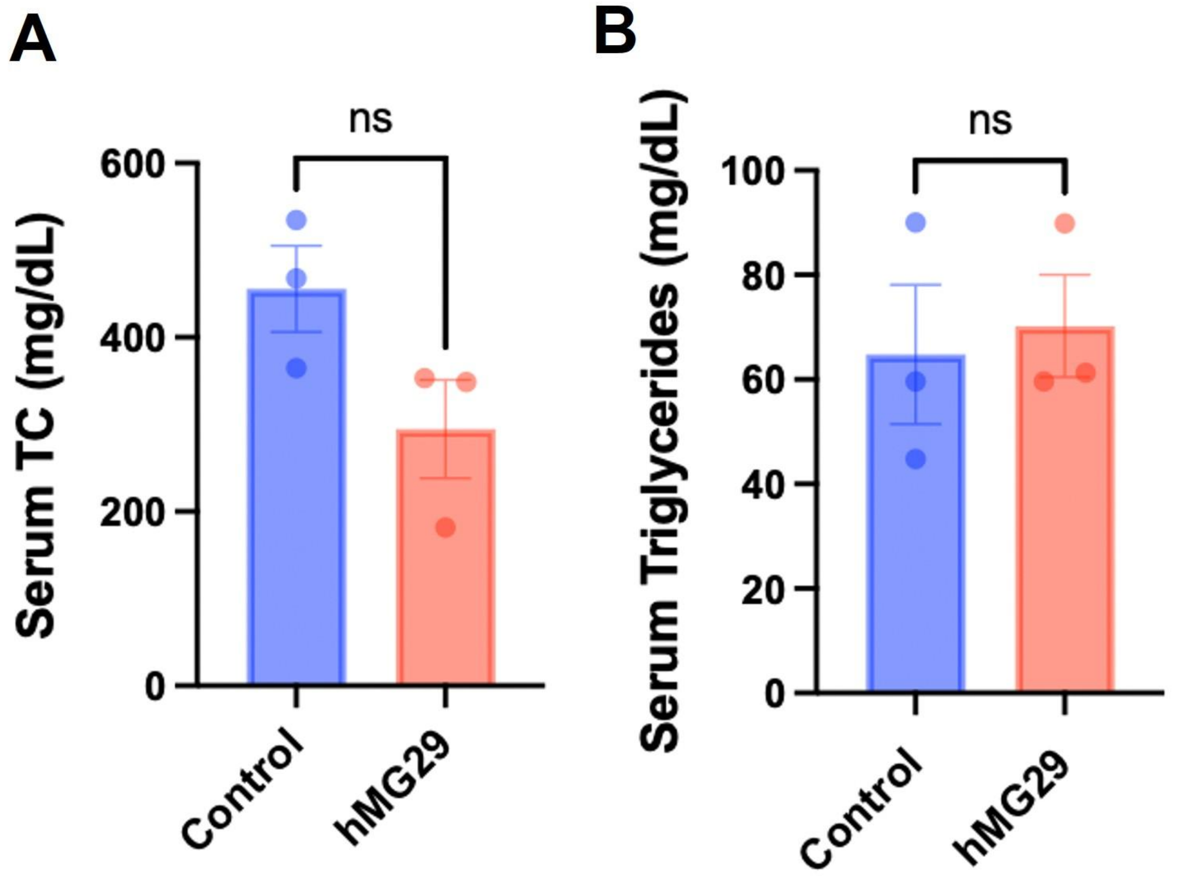

Figure S3: Effects of MG29 overexpression on serum lipid levels in HFD mice. (A) Serum total cholesterol (TC) and (B) serum triglycerides levels in control and MG29-treated mice. Mice were fed a high-fat diet (HFD, 50 kcal%) and received intravenous (i.v) injection of MyoAAV-MHCK7-MG29. Serum samples were collected 5weeks post-injection. Data as mean ± SD with individual data points. Statistical analysis was performed using an unpaired Student's t-test. ns, not significant.

Western Blotting Raw data

MG29 antibody-EDL

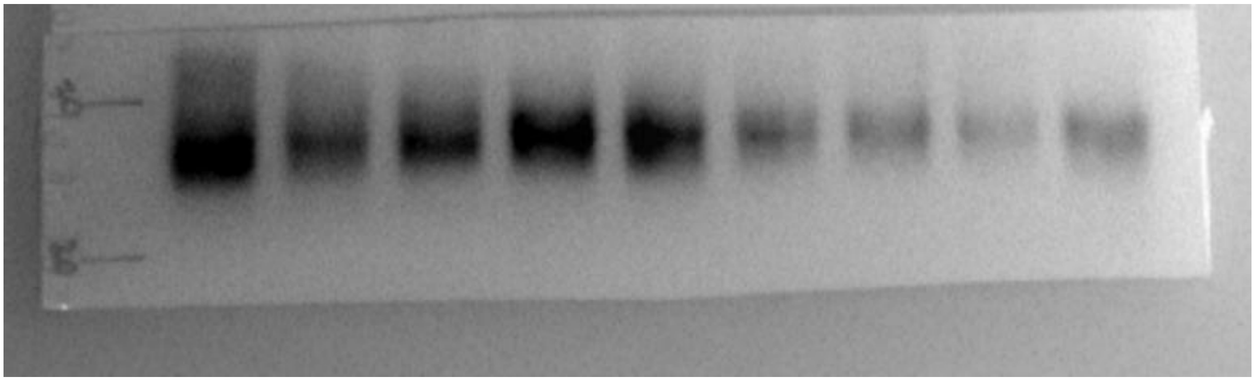

SDHA antibody-EDL

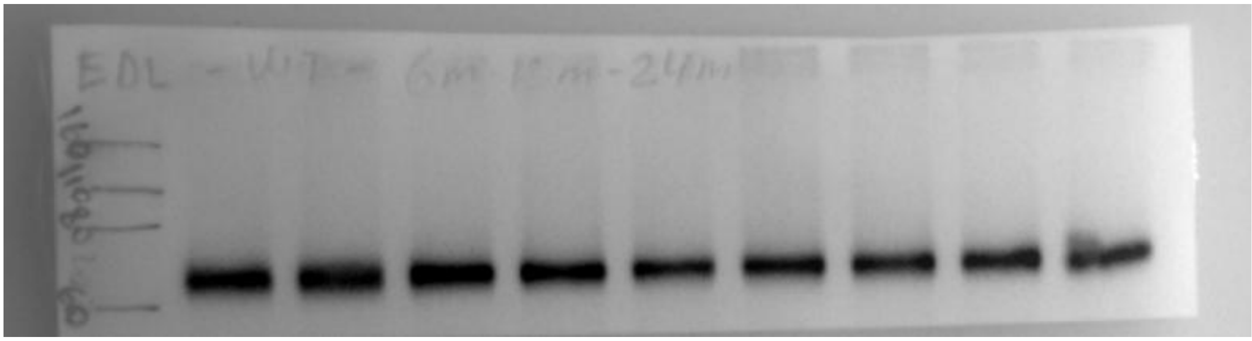

GAPDH antibody-EDL

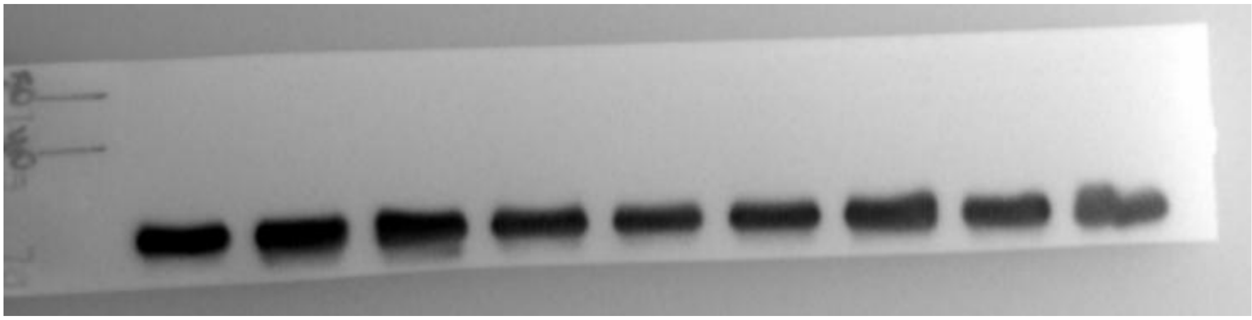

MG29 antibody-SOL

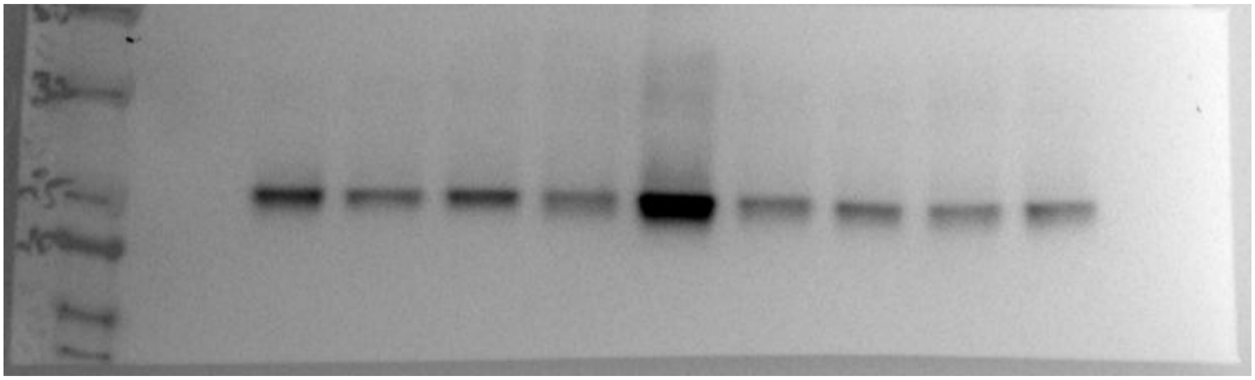

SDHA antibody-SOL

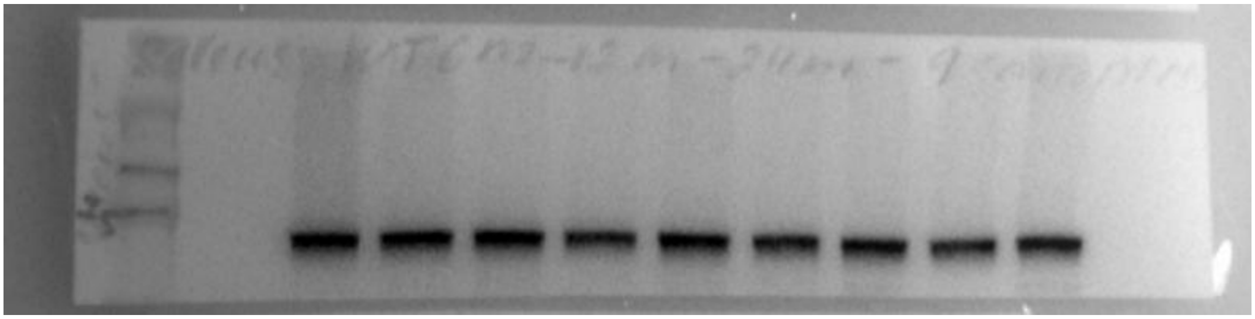

GAPDH antibody-SOL

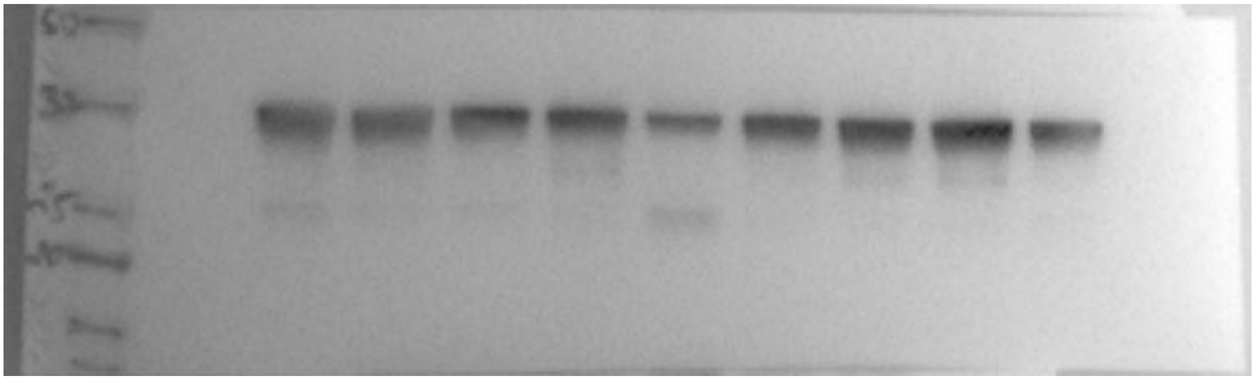

Image Report: EDL-MG29-Gel2_2016-09-26 16hr 09min_25.1sec-quant

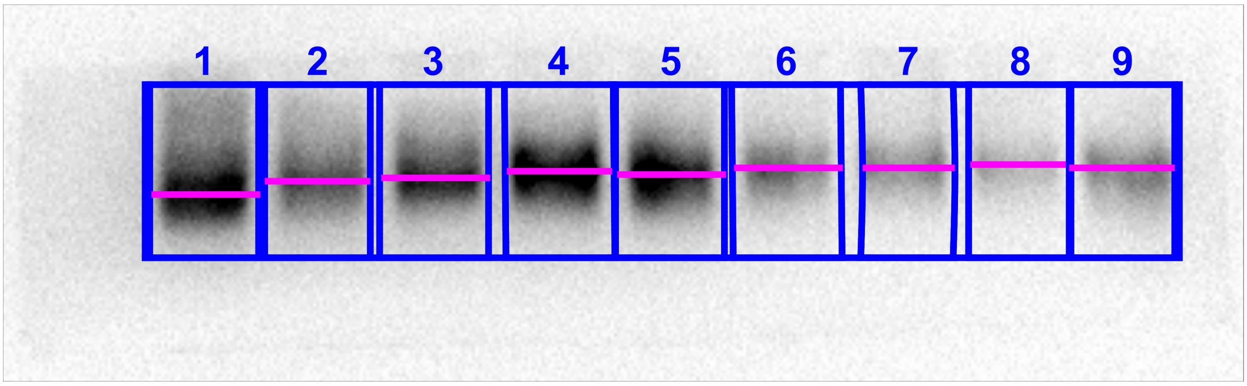

S:\RESGushchina_LiubovGushchina\LG Manuscripts\2026_MG29\ChemiDocs\EDL-MG29-Gel2_2016-09-26 16hr 09min_25.1sec-quant.scn

---

Acquisition Information

Imager	ChemiDoc™ XRS+
Exposure Time (sec)	25.120 (Signal Accumulation)
Flat Field	Applied (Lens)
Serial Number	721BR04926
Software Version	5.2.1
Application	Chemi Hi Resolution
Excitation Source	No Illumination
Emission Filter	No Filter
Binning	2x2

Image Information

Acquisition Date	9/26/2016 4:10:26 PM
User Name	Gushchina
Image Area (mm)	X: 86.4 Y: 26.2
Pixel Size (µm)	X: 229.9 Y: 229.9
Data Range (Int)	0 - 31524

Analysis Settings

Detection	Lane detection:<br>Manually created lanes<br><br>Band detection:<br>Automatically detected bands with sensitivity: High<br>Manually adjusted bands<br><br>Lane Background Subtraction:<br>Lane background subtracted with disk size: 10<br><br>Lane width: Variable

Lane Statistics

Lane No.	Adj. Total Band Vol. (Int)	Total Band Vol. (Int)	Adj. Total Lane Vol. (Int)	Total Lane Vol. (Int)	Bkgd. Vol. (Int)	Norm. Factor
1	8,801,595	14,913,756	9,609,567	19,367,997	9,758,430	N/A
2	6,450,368	10,720,224	7,159,136	13,337,248	6,178,112	N/A
3	8,098,233	11,688,204	8,885,349	14,355,858	5,470,509	N/A
4	11,059,158	14,986,158	11,483,604	16,989,984	5,506,380	N/A
5	9,871,620	13,720,080	10,109,286	15,269,232	5,159,946	N/A
6	4,991,613	7,381,011	5,177,667	8,509,908	3,332,241	N/A
7	3,576,496	5,430,516	3,704,176	6,346,760	2,642,584	N/A
8	2,514,100	4,217,891	2,608,123	4,896,481	2,288,358	N/A
9	4,655,456	6,805,280	4,759,808	7,640,032	2,880,224	N/A

Lane And Band Analysis

Lane 1

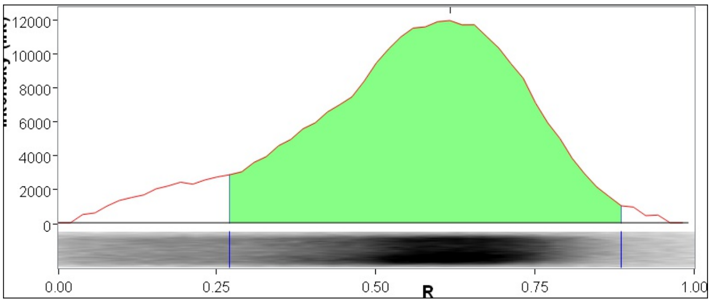

Band No.	Band Label	Mol. Wt. (KDa)	Relative Front	Adj. Volume (Int)	Volume (Int)	Abs. Quant.	Rel. Quant.	Band %	Lane %
1		N/A	0.635	8,801,595	14,913,756	N/A	N/A	100.0	91.6

Band Detection	Automatically detected bands with sensitivity: High
Lane Background	Lane background subtracted with disk size: 10
Lane Width	7.59 mm

Lane 2

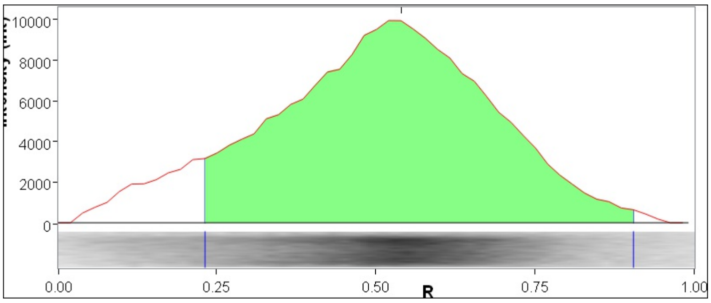

Band No.	Band Label	Mol. Wt. (KDa)	Relative Front	Adj. Volume (Int)	Volume (Int)	Abs. Quant.	Rel. Quant.	Band %	Lane %
1		N/A	0.558	6,450,368	10,720,224	N/A	N/A	100.0	90.1

Band Detection	Automatically detected bands with sensitivity: High
Lane Background	Lane background subtracted with disk size: 10
Lane Width	7.36 mm

Lane 3

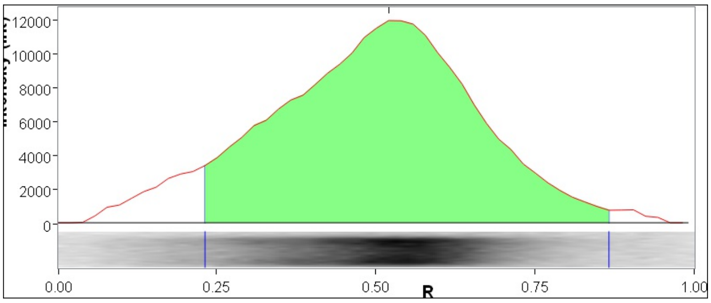

Band No.	Band Label	Mol. Wt. (KDa)	Relative Front	Adj. Volume (Int)	Volume (Int)	Abs. Quant.	Rel. Quant.	Band %	Lane %
1		N/A	0.538	8,098,233	11,688,204	N/A	N/A	100.0	91.1

Band Detection	Automatically detected bands with sensitivity: High
Lane Background	Lane background subtracted with disk size: 10
Lane Width	7.59 mm

Lane 4

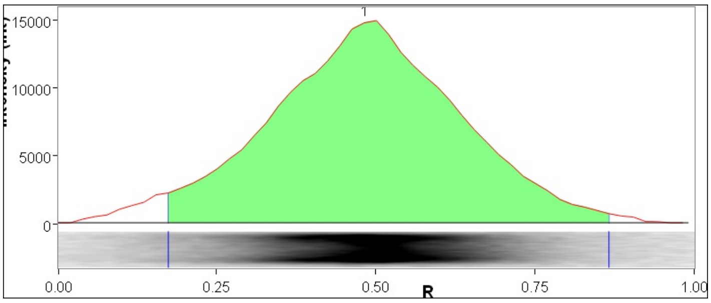

Band No.	Band Label	Mol. Wt. (KDa)	Relative Front	Adj. Volume (Int)	Volume (Int)	Abs. Quant.	Rel. Quant.	Band %	Lane %
1		N/A	0.500	11,059,158	14,986,158	N/A	N/A	100.0	96.3

Band Detection	Automatically detected bands with sensitivity: High
Lane Background	Lane background subtracted with disk size: 10
Lane Width	7.59 mm

Lane 5

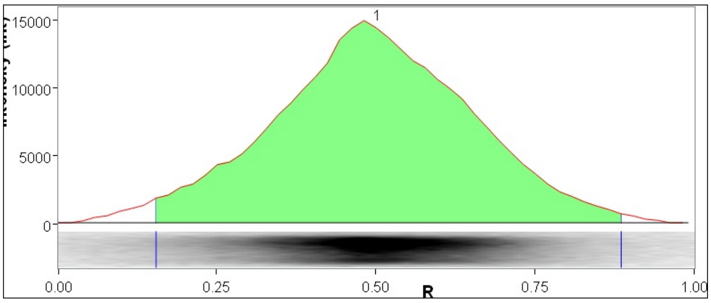

Band No.	Band Label	Mol. Wt. (KDa)	Relative Front	Adj. Volume (Int)	Volume (Int)	Abs. Quant.	Rel. Quant.	Band %	Lane %
1		N/A	0.519	9,871,620	13,720,080	N/A	N/A	100.0	97.6

Band Detection	Automatically detected bands with sensitivity: High
Lane Background	Lane background subtracted with disk size: 10
Lane Width	7.59 mm

Lane 6

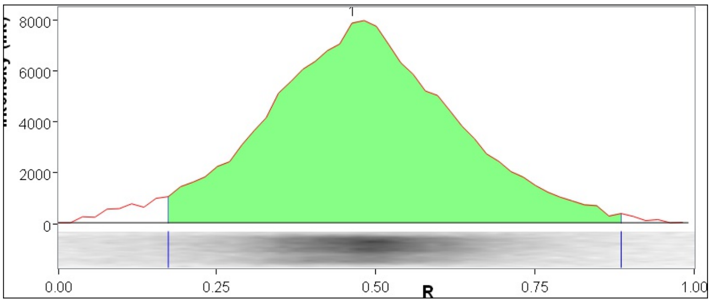

Band No.	Band Label	Mol. Wt. (KDa)	Relative Front	Adj. Volume (Int)	Volume (Int)	Abs. Quant.	Rel. Quant.	Band %	Lane %
1		N/A	0.481	4,991,613	7,381,011	N/A	N/A	100.0	96.4

Band Detection	Automatically detected bands with sensitivity: High
Lane Background	Lane background subtracted with disk size: 10
Lane Width	7.59 mm

Lane 7

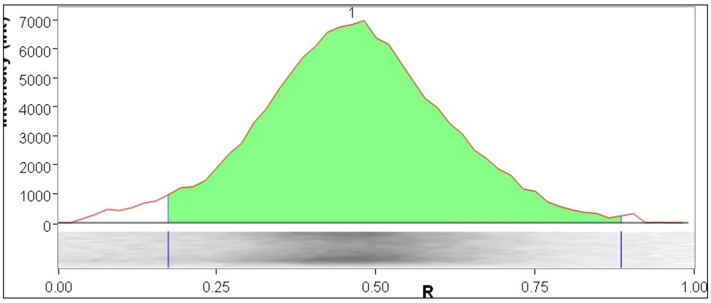

Band No.	Band Label	Mol. Wt. (KDa)	Relative Front	Adj. Volume (Int)	Volume (Int)	Abs. Quant.	Rel. Quant.	Band %	Lane %
1		N/A	0.481	3,576,496	5,430,516	N/A	N/A	100.0	96.6

Band Detection	Automatically detected bands with sensitivity: High
Lane Background	Lane background subtracted with disk size: 10
Lane Width	6.44 mm

Lane 8

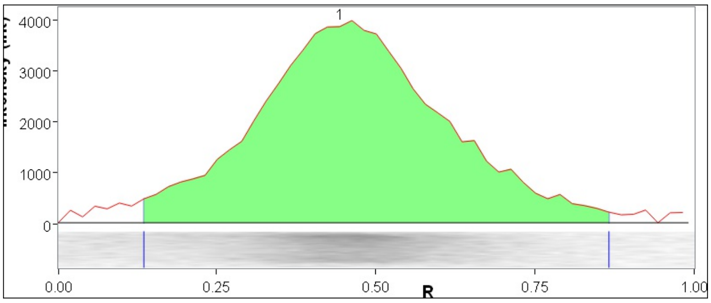

Band No.	Band Label	Mol. Wt. (KDa)	Relative Front	Adj. Volume (Int)	Volume (Int)	Abs. Quant.	Rel. Quant.	Band %	Lane %
1		N/A	0.462	2,514,100	4,217,891	N/A	N/A	100.0	96.4

Band Detection	Automatically detected bands with sensitivity: High
Lane Background	Lane background subtracted with disk size: 10
Lane Width	7.13 mm

Lane 9

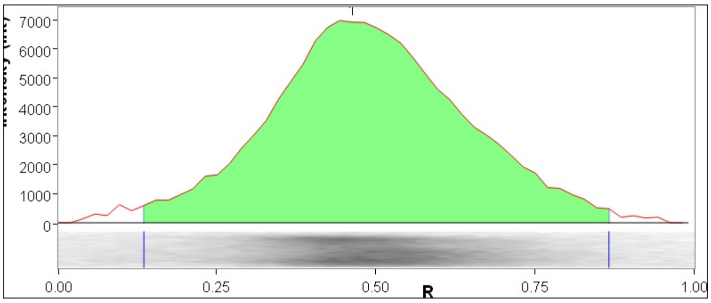

Band No.	Band Label	Mol. Wt. (KDa)	Relative Front	Adj. Volume (Int)	Volume (Int)	Abs. Quant.	Rel. Quant.	Band %	Lane %
1		N/A	0.481	4,655,456	6,805,280	N/A	N/A	100.0	97.8

Band Detection	Automatically detected bands with sensitivity: High
Lane Background	Lane background subtracted with disk size: 10
Lane Width	7.36 mm

Image Report: EDL-SHDA_2016-09-26 15hr 49min_1sec-quant

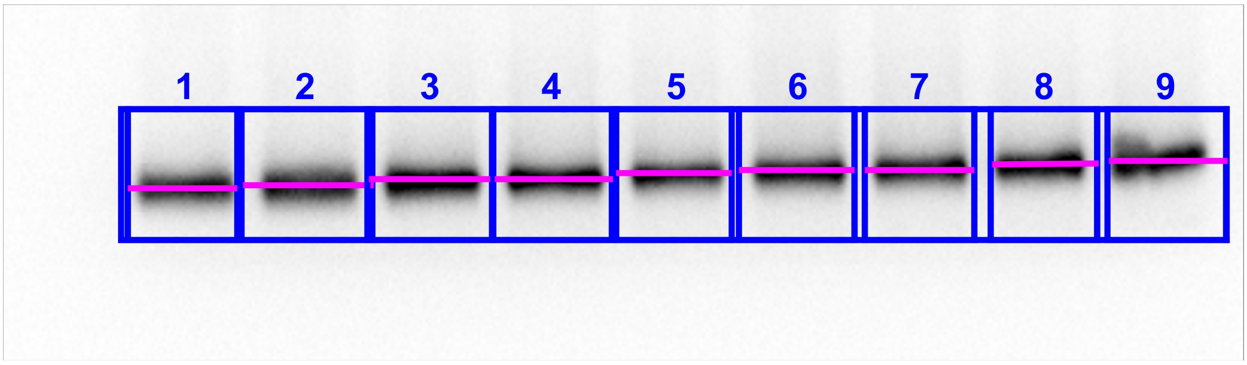

S:\RESGushchina_LiubovGushchina\LG Manuscripts\2026_MG29\ChemiDocs\EDL\EDL-SHDA_2016-09-26 15hr 49min_1sec-quant.scn

Acquisition Information

Imager	ChemiDoc™ XRS+
Exposure Time (sec)	1.000 (Signal Accumulation)
Flat Field	Applied (Lens)
Serial Number	721BR04926
Software Version	5.2.1
Application	Chemi Hi Resolution
Excitation Source	No Illumination
Emission Filter	No Filter
Binning	2x2

Image Information

Acquisition Date	9/26/2016 3:49:10 PM
User Name	Gushchina
Image Area (mm)	X: 82.1 Y: 23.5
Pixel Size (µm)	X: 201.1 Y: 201.1
Data Range (Int)	0 - 32392

Analysis Settings

Detection	Lane detection:<br>Manually created lanes<br><br>Band detection:<br>Automatically detected bands with sensitivity: High<br>Manually adjusted bands<br><br>Lane Background Subtraction:<br>Lane background subtracted with disk size: 10<br><br>Lane width: Variable

Lane Statistics

Lane No.	Adj. Total Band Vol. (Int)	Total Band Vol. (Int)	Adj. Total Lane Vol. (Int)	Total Lane Vol. (Int)	Bkgd. Vol. (Int)	Norm. Factor

1	6,724,044	8,413,056	6,733,872	8,753,940	2,020,068	N/A
2	7,429,970	10,042,607	7,437,882	10,467,619	3,029,737	N/A
3	8,170,726	10,865,943	8,181,509	11,401,936	3,220,427	N/A
4	7,224,282	9,875,307	7,245,420	10,603,554	3,358,134	N/A
5	5,751,110	7,949,866	5,807,350	8,855,216	3,047,866	N/A
6	6,259,208	8,412,326	6,405,470	9,725,682	3,320,212	N/A
7	6,281,928	8,758,836	6,314,364	9,415,224	3,100,860	N/A
8	6,454,315	8,596,455	6,500,970	9,379,510	2,878,540	N/A
9	6,849,453	9,207,939	6,873,165	9,754,407	2,881,242	N/A

Lane And Band Analysis

Lane 1

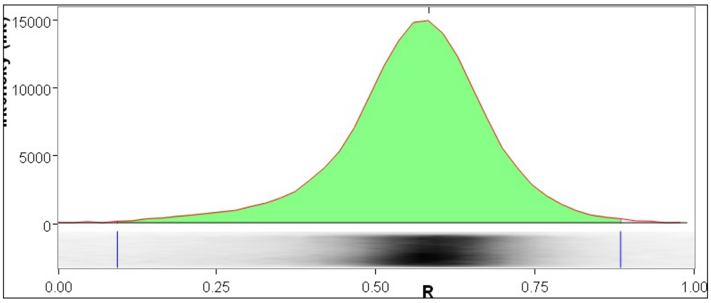

Band No.	Band Label	Mol. Wt. (KDa)	Relative Front	Adj. Volume (Int)	Volume (Int)	Abs. Quant.	Rel. Quant.	Band %	Lane %
1		N/A	0.605	6,724,044	8,413,056	N/A	N/A	100.0	99.9

Band Detection	Automatically detected bands with sensitivity: High
Lane Background	Lane background subtracted with disk size: 10
Lane Width	7.24 mm

Lane 2

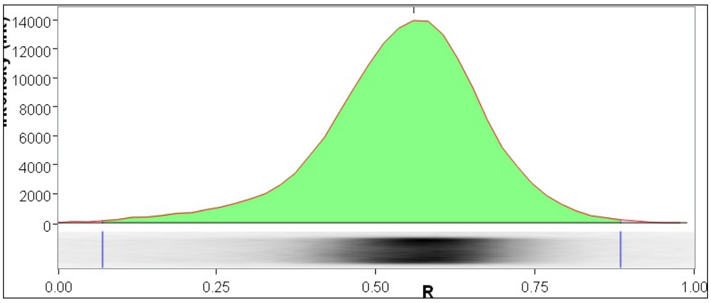

Band No.	Band Label	Mol. Wt. (KDa)	Relative Front	Adj. Volume (Int)	Volume (Int)	Abs. Quant.	Rel. Quant.	Band %	Lane %
1		N/A	0.581	7,429,970	10,042,607	N/A	N/A	100.0	99.9

Band Detection	Automatically detected bands with sensitivity: High
Lane Background	Lane background subtracted with disk size: 10
Lane Width	8.65 mm

Lane 3

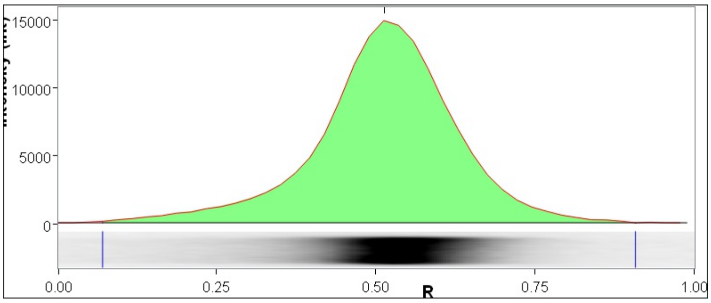

Band No.	Band Label	Mol. Wt. (KDa)	Relative Front	Adj. Volume (Int)	Volume (Int)	Abs. Quant.	Rel. Quant.	Band %	Lane %
1		N/A	0.535	8,170,726	10,865,943	N/A	N/A	100.0	99.9

Band Detection	Automatically detected bands with sensitivity: High
Lane Background	Lane background subtracted with disk size: 10
Lane Width	8.25 mm

Lane 4

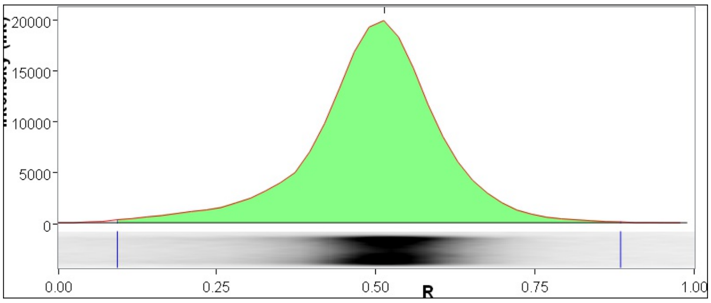

Band No.	Band Label	Mol. Wt. (KDa)	Relative Front	Adj. Volume (Int)	Volume (Int)	Abs. Quant.	Rel. Quant.	Band %	Lane %
1		N/A	0.535	7,224,282	9,875,307	N/A	N/A	100.0	99.7

Band Detection	Automatically detected bands with sensitivity: High
Lane Background	Lane background subtracted with disk size: 10
Lane Width	7.84 mm

Lane 5

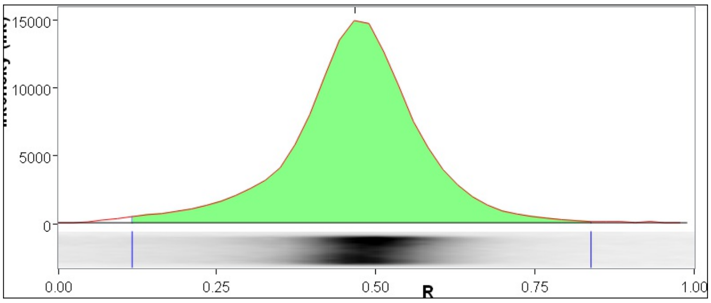

Band No.	Band Label	Mol. Wt. (KDa)	Relative Front	Adj. Volume (Int)	Volume (Int)	Abs. Quant.	Rel. Quant.	Band %	Lane %
1		N/A	0.488	5,751,110	7,949,866	N/A	N/A	100.0	99.0

Band Detection	Automatically detected bands with sensitivity: High

Lane Background	Lane background subtracted with disk size: 10
Lane Width	7.64 mm

Lane 6

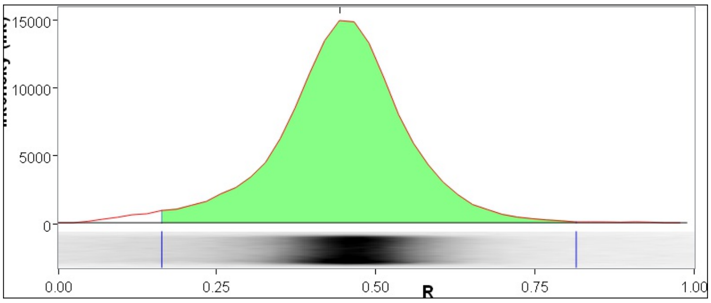

Band No.	Band Label	Mol. Wt. (KDa)	Relative Front	Adj. Volume (Int)	Volume (Int)	Abs. Quant.	Rel. Quant.	Band %	Lane %
1		N/A	0.465	6,259,208	8,412,326	N/A	N/A	100.0	97.7

Band Detection	Automatically detected bands with sensitivity: High
Lane Background	Lane background subtracted with disk size: 10
Lane Width	7.64 mm

Lane 7

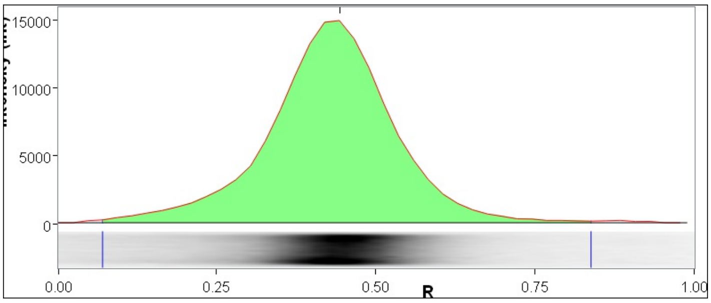

Band No.	Band Label	Mol. Wt. (KDa)	Relative Front	Adj. Volume (Int)	Volume (Int)	Abs. Quant.	Rel. Quant.	Band %	Lane %
1		N/A	0.465	6,281,928	8,758,836	N/A	N/A	100.0	99.5

Band Detection	Automatically detected bands with sensitivity: High
Lane Background	Lane background subtracted with disk size: 10
Lane Width	7.24 mm

Lane 8

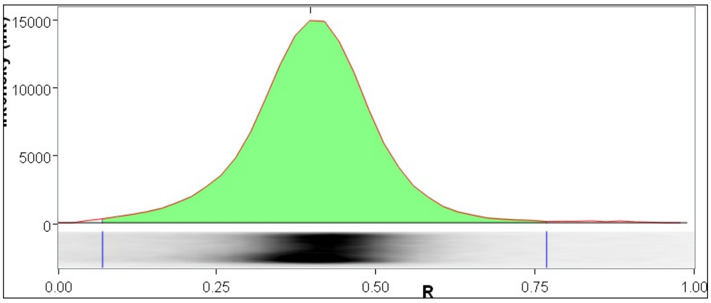

Band No.	Band Label	Mol. Wt. (KDa)	Relative Front	Adj. Volume (Int)	Volume (Int)	Abs. Quant.	Rel. Quant.	Band %	Lane %
1		N/A	0.419	6,454,315	8,596,455	N/A	N/A	100.0	99.3

Band Detection	Automatically detected bands with sensitivity: High
Lane Background	Lane background subtracted with disk size: 10
Lane Width	7.04 mm

Lane 9

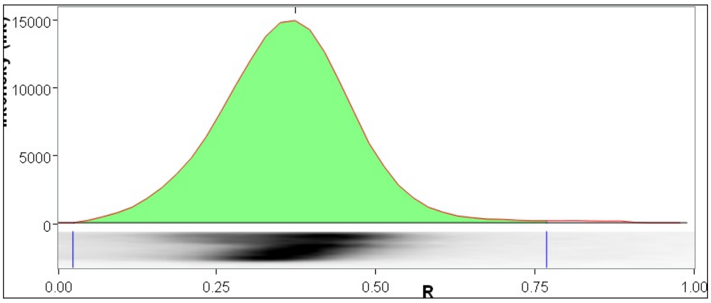

Band No.	Band Label	Mol. Wt. (KDa)	Relative Front	Adj. Volume (Int)	Volume (Int)	Abs. Quant.	Rel. Quant.	Band %	Lane %
1		N/A	0.395	6,849,453	9,207,939	N/A	N/A	100.0	99.7

Band Detection	Automatically detected bands with sensitivity: High
Lane Background	Lane background subtracted with disk size: 10
Lane Width	7.84 mm

Image Report: EDL-GAPDH_2016-09-29 12hr 06min_1.9sec-quant

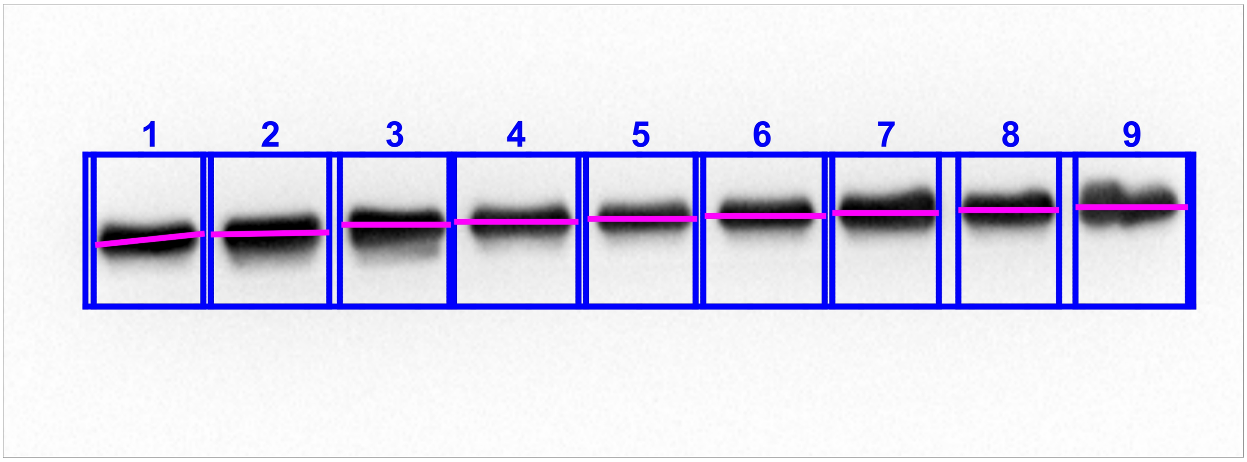

S:\RESGushchina_LiubovGushchina\LG Manuscripts\2026_MG29\ChemiDocs\EDL\EDL-GAPDH_2016-09-29 12hr 06min_1.9sec-quant.scn

Acquisition Information

Imager	ChemiDoc™ XRS+
Exposure Time (sec)	1.930 (Signal Accumulation)
Flat Field	Applied (Lens)
Serial Number	721BR04926
Software Version	5.2.1
Application	Chemi Hi Resolution
Excitation Source	No Illumination
Emission Filter	No Filter
Binning	2x2

Image Information

Acquisition Date	9/29/2016 12:06:51 PM
User Name	Gushchina
Image Area (mm)	X: 81.3 Y: 29.7
Pixel Size (µm)	X: 194.0 Y: 194.0
Data Range (Int)	0 - 42196

Analysis Settings

Detection	Lane detection:<br>Manually created lanes<br><br>Band detection:<br>Automatically detected bands with sensitivity: High<br>Manually adjusted bands<br><br>Lane Background Subtraction:<br>Lane background subtracted with disk size: 10<br><br>Lane width: Variable

Lane Statistics

Lane No.	Adj. Total Band Vol. (Int)	Total Band Vol. (Int)	Adj. Total Lane Vol. (Int)	Total Lane Vol. (Int)	Bkgd. Vol. (Int)	Norm. Factor
1	12,926,394	14,734,140	13,082,127	15,761,038	2,678,911	N/A
2	14,858,160	17,801,240	14,942,040	18,841,560	3,899,520	N/A
3	14,406,246	17,357,366	14,451,534	18,189,866	3,738,332	N/A
4	11,732,364	14,582,778	11,784,318	15,476,286	3,691,968	N/A
5	9,789,571	11,606,234	9,989,260	12,887,951	2,898,691	N/A
6	11,300,297	13,919,623	11,375,450	14,881,360	3,505,910	N/A
7	13,421,556	16,018,092	13,490,244	16,761,240	3,270,996	N/A
8	11,717,794	13,785,164	11,838,494	14,638,870	2,800,376	N/A
9	12,022,136	13,657,200	12,230,680	14,652,382	2,421,702	N/A

Lane And Band Analysis

Lane 1

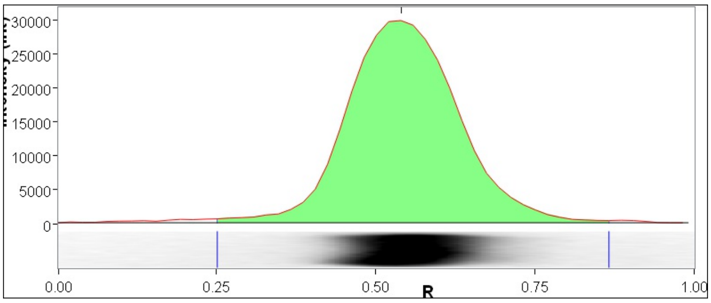

Band No.	Band Label	Mol. Wt. (KDa)	Relative Front	Adj. Volume (Int)	Volume (Int)	Abs. Quant.	Rel. Quant.	Band %	Lane %
1		N/A	0.558	12,926,394	14,734,140	N/A	N/A	100.0	98.8

Band Detection	Automatically detected bands with sensitivity: High
Lane Background	Lane background subtracted with disk size: 10
Lane Width	7.18 mm

Lane 2

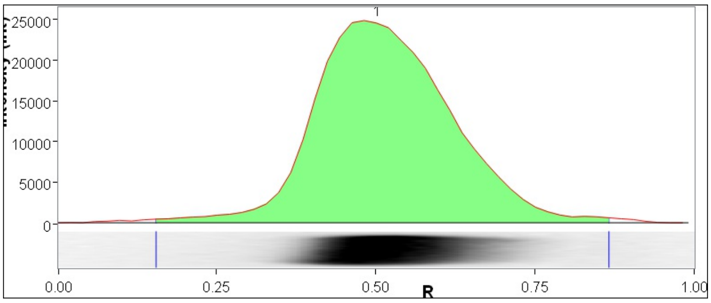

Band No.	Band Label	Mol. Wt. (KDa)	Relative Front	Adj. Volume (Int)	Volume (Int)	Abs. Quant.	Rel. Quant.	Band %	Lane %
1		N/A	0.519	14,858,160	17,801,240	N/A	N/A	100.0	99.4

Band Detection	Automatically detected bands with sensitivity: High
Lane Background	Lane background subtracted with disk size: 10
Lane Width	7.76 mm

Lane 3

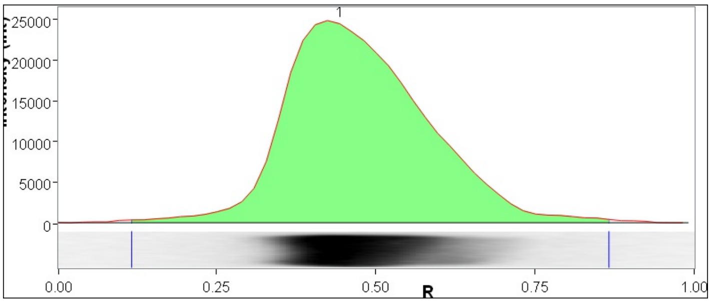

Band No.	Band Label	Mol. Wt. (KDa)	Relative Front	Adj. Volume (Int)	Volume (Int)	Abs. Quant.	Rel. Quant.	Band %	Lane %
1		N/A	0.462	14,406,246	17,357,366	N/A	N/A	100.0	99.7

Band Detection	Automatically detected bands with sensitivity: High
Lane Background	Lane background subtracted with disk size: 10
Lane Width	7.18 mm

Lane 4

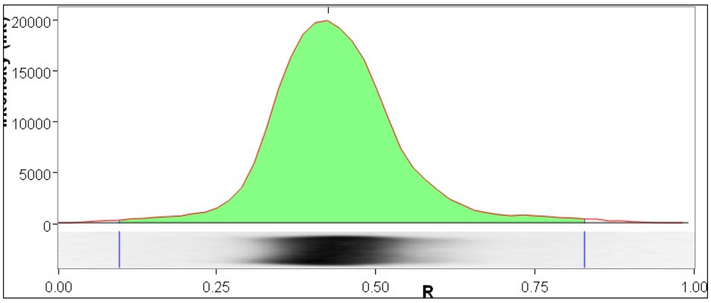

Band No.	Band Label	Mol. Wt. (KDa)	Relative Front	Adj. Volume (Int)	Volume (Int)	Abs. Quant.	Rel. Quant.	Band %	Lane %
1		N/A	0.442	11,732,364	14,582,778	N/A	N/A	100.0	99.6

Band Detection	Automatically detected bands with sensitivity: High
Lane Background	Lane background subtracted with disk size: 10
Lane Width	8.15 mm

Lane 5

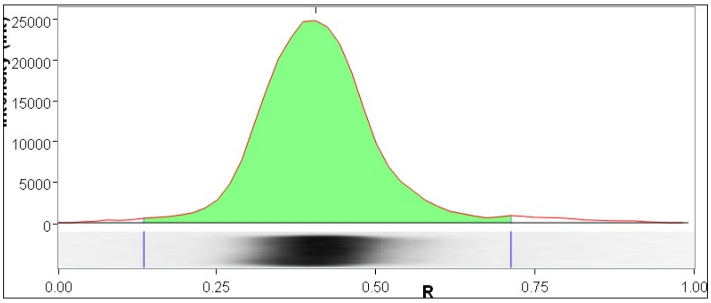

Band No.	Band Label	Mol. Wt. (KDa)	Relative Front	Adj. Volume (Int)	Volume (Int)	Abs. Quant.	Rel. Quant.	Band %	Lane %

1		N/A	0.423	9,789,571	11,606,234	N/A	N/A	100.0	98.0

Band Detection	Automatically detected bands with sensitivity: High
Lane Background	Lane background subtracted with disk size: 10
Lane Width	7.18 mm

Lane 6

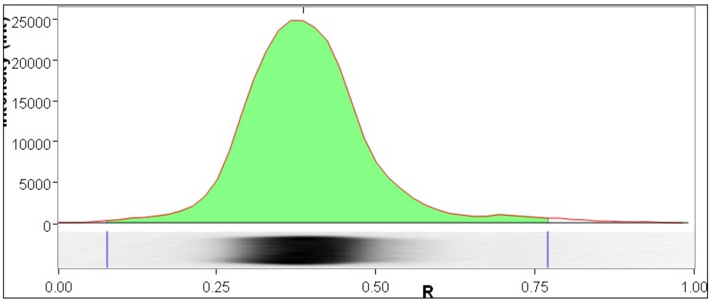

Band No.	Band Label	Mol. Wt. (KDa)	Relative Front	Adj. Volume (Int)	Volume (Int)	Abs. Quant.	Rel. Quant.	Band %	Lane %
1		N/A	0.404	11,300,297	13,919,623	N/A	N/A	100.0	99.3

Band Detection	Automatically detected bands with sensitivity: High
Lane Background	Lane background subtracted with disk size: 10
Lane Width	7.95 mm

Lane 7

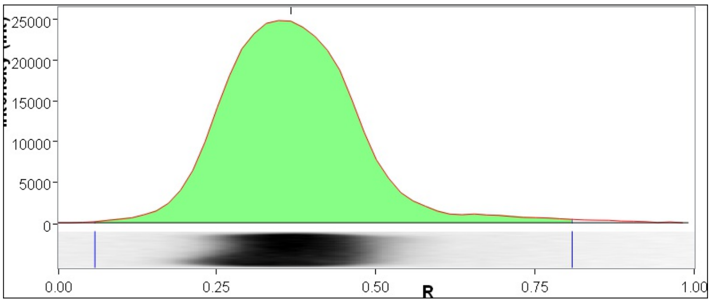

Band No.	Band Label	Mol. Wt. (KDa)	Relative Front	Adj. Volume (Int)	Volume (Int)	Abs. Quant.	Rel. Quant.	Band %	Lane %
1		N/A	0.385	13,421,556	16,018,092	N/A	N/A	100.0	99.5

Band Detection	Automatically detected bands with sensitivity: High
Lane Background	Lane background subtracted with disk size: 10
Lane Width	6.98 mm

Lane 8

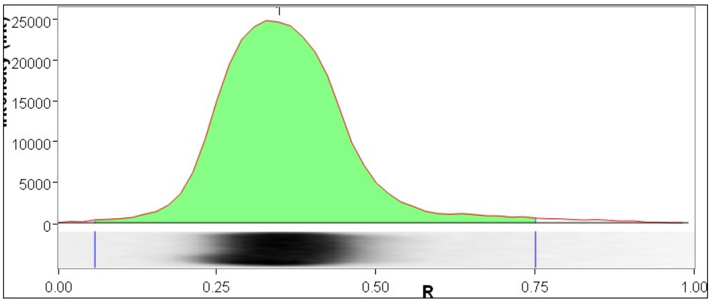

Band No.	Band Label	Mol. Wt. (KDa)	Relative Front	Adj. Volume (Int)	Volume (Int)	Abs. Quant.	Rel. Quant.	Band %	Lane %
1		N/A	0.365	11,717,794	13,785,164	N/A	N/A	100.0	99.0

Band Detection	Automatically detected bands with sensitivity: High
Lane Background	Lane background subtracted with disk size: 10
Lane Width	6.59 mm

Lane 9

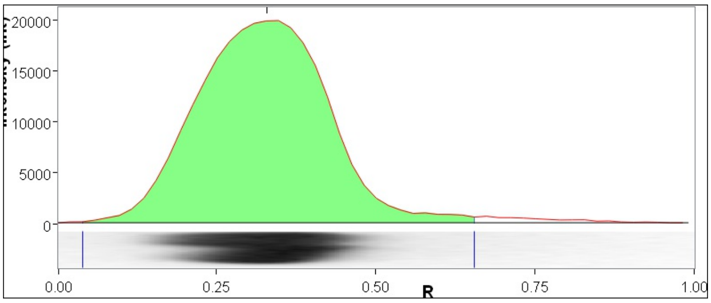

Band No.	Band Label	Mol. Wt. (KDa)	Relative Front	Adj. Volume (Int)	Volume (Int)	Abs. Quant.	Rel. Quant.	Band %	Lane %
1		N/A	0.346	12,022,136	13,657,200	N/A	N/A	100.0	98.3

Band Detection	Automatically detected bands with sensitivity: High
Lane Background	Lane background subtracted with disk size: 10
Lane Width	7.37 mm

EDL-MG29-Gel2_2016-09-26 16hr 09min_25.1sec-quant

Lane No.	Adj. Total Band Vol. (Int)	Total Band Vol. (Int)	Adj. Total Lane Vol. (Int)	Total Lane Vol. (Int)	Bkgd. Vol. (Int)	Norm. Factor
1	8801595	14913756	9609567	19367997	9758430	N/A
2	6450368	10720224	7159136	13337248	6178112	N/A
3	8098233	11688204	8885349	14355858	5470509	N/A
4	11059158	14986158	11483604	16989984	5506380	N/A
5	9871620	13720080	10109286	15269232	5159946	N/A
6	4991613	7381011	5177667	8509908	3332241	N/A
7	3576496	5430516	3704176	6346760	2642584	N/A
8	2514100	4217891	2608123	4896481	2288358	N/A
9	4655456	6805280	4759808	7640032	2880224	N/A

EDL-SHDA_2016-09-26 15hr 49min_1sec-quant

Lane No.	Adj. Total Band Vol. (Int)	Total Band Vol. (Int)	Adj. Total Lane Vol. (Int)	Total Lane Vol. (Int)	Bkgd. Vol. (Int)	Norm. Factor
1	6724044	8413056	6733872	8753940	2020068	N/A
2	7429970	10042607	7437882	10467619	3029737	N/A
3	8170726	10865943	8181509	11401936	3220427	N/A
4	7224282	9875307	7245420	10603554	3358134	N/A
5	5751110	7949866	5807350	8855216	3047866	N/A
6	6259208	8412326	6405470	9725682	3320212	N/A
7	6281928	8758836	6314364	9415224	3100860	N/A
8	6454315	8596455	6500970	9379510	2878540	N/A
9	6849453	9207939	6873165	9754407	2881242	N/A

EDL-GAPDH_2016-09-29 12hr 06min_1.9sec-quant

Lane No.	Adj. Total Band Vol. (Int)	Total Band Vol. (Int)	Adj. Total Lane Vol. (Int)	Total Lane Vol. (Int)	Bkgd. Vol. (Int)	Norm. Factor
1	12926394	14734140	13082127	15761038	2678911	N/A
2	14858160	17801240	14942040	18841560	3899520	N/A
3	14406246	17357366	14451534	18189866	3738332	N/A
4	11732364	14582778	11784318	15476286	3691968	N/A
5	9789571	11606234	9989260	12887951	2898691	N/A

6	11300297	13919623	11375450	14881360	3505910	N/A
7	13421556	16018092	13490244	16761240	3270996	N/A
8	11717794	13785164	11838494	14638870	2800376	N/A
9	12022136	13657200	12230680	14652382	2421702	N/A

	**EDL MG29/SHDA ratio**	**Norm. factor**	**Relative quant**	**Average**	**SD**
EDL-6mo-01	1.3090	1.0561	1.2395	1.0000	0.2154
EDL-6mo-02	0.8682		0.8221		
EDL-6mo-03	0.9911		0.9385		
EDL-12mo-01	1.5308		1.4495	1.2767	0.4601
EDL-12mo-02	1.7165		1.6253		
EDL-12mo-03	0.7975		0.7551		
EDL-24mo-01	0.5693		0.5391	0.5172	0.1387
EDL-24mo-02	0.3895		0.3688		
EDL-24mo-03	0.6797		0.6436		

	**EDL MG29/GAPDH ratio**	**Norm. factor**	**Relative quant**	**Average**	**SD**
EDL-6mo-01	0.6809	0.5591	1.2180	1.0000	0.2208
EDL-6mo-02	0.4341		0.7765		
EDL-6mo-03	0.5621		1.0055		
EDL-12mo-01	0.9426		1.6861	1.4266	0.5544
EDL-12mo-02	1.0084		1.8037		
EDL-12mo-03	0.4417		0.7901		
EDL-24mo-01	0.2665		0.4767	0.5177	0.1585
EDL-24mo-02	0.2146		0.3838		
EDL-24mo-03	0.3872		0.6927		

Image Report: Gel2-Sol-MG29_2016-10-04 12hr 58min_30sec-quant

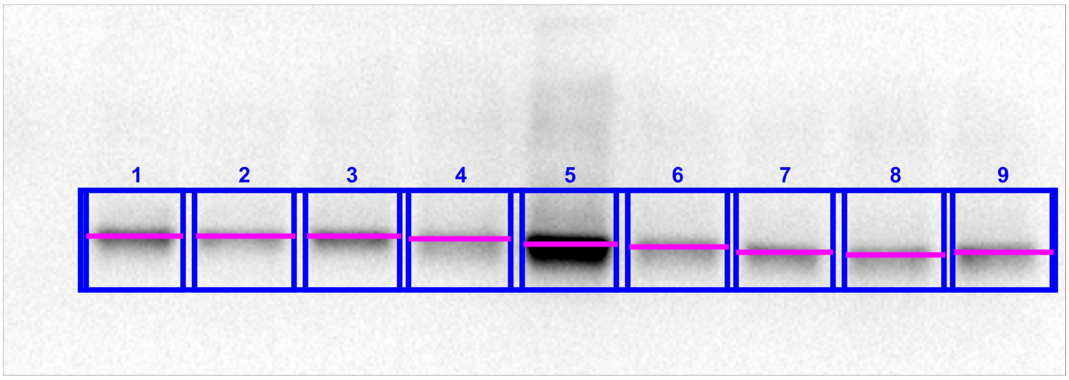

S:\RESGushchina_LiubovGushchina\LG Manuscripts\2026_MG29\ChemiDocs\Gel2-Sol-MG29_2016-10-04 12hr 58min_30sec-quant.scn

Acquisition Information

Imager	ChemiDoc XRS+
Exposure Time (sec)	30.000 (Signal Accumulation)
Flat Field	Applied (Lens)
Serial Number	721BR04926
Software Version	5.2.1
Application	Chemi Hi Resolution
Excitation Source	No Illumination
Emission Filter	No Filter
Binning	2x2

Image Information

Acquisition Date	10/4/2016 12:59:58 PM
User Name	Gushchina
Image Area (mm)	X: 66.4 Y: 23.2
Pixel Size (µm)	X: 168.1 Y: 168.1
Data Range (Int)	0 - 65535

Analysis Settings

Detection	Lane detection:<br>Manually created lanes<br><br>Band detection:<br>Automatically detected bands with sensitivity: High<br>Manually adjusted bands<br><br>Lane Background Subtraction:<br>Lane background subtracted with disk size: 10<br><br>Lane width: Variable

Lane Statistics

Lane No.	Adj. Total Band Vol. (Int)	Total Band Vol. (Int)	Adj. Total Lane Vol. (Int)	Total Lane Vol. (Int)	Bkgd. Vol. (Int)	Norm. Factor
1	6,144,876	8,882,424	6,191,712	9,770,544	3,578,832	N/A
2	3,935,727	6,430,082	3,997,591	7,516,920	3,519,329	N/A
3	5,541,550	8,395,660	5,591,950	9,325,050	3,733,100	N/A
4	4,626,994	8,084,120	4,715,154	9,578,660	4,863,506	N/A
5	18,816,455	26,592,300	18,846,310	27,875,050	9,028,740	N/A
6	4,694,671	8,151,507	4,825,577	9,772,255	4,946,678	N/A
7	4,602,564	7,857,864	4,646,232	9,034,020	4,387,788	N/A
8	4,200,018	7,591,734	4,277,533	8,926,805	4,649,272	N/A
9	4,953,153	7,725,193	5,100,857	9,163,420	4,062,563	N/A

Lane And Band Analysis

Lane 1

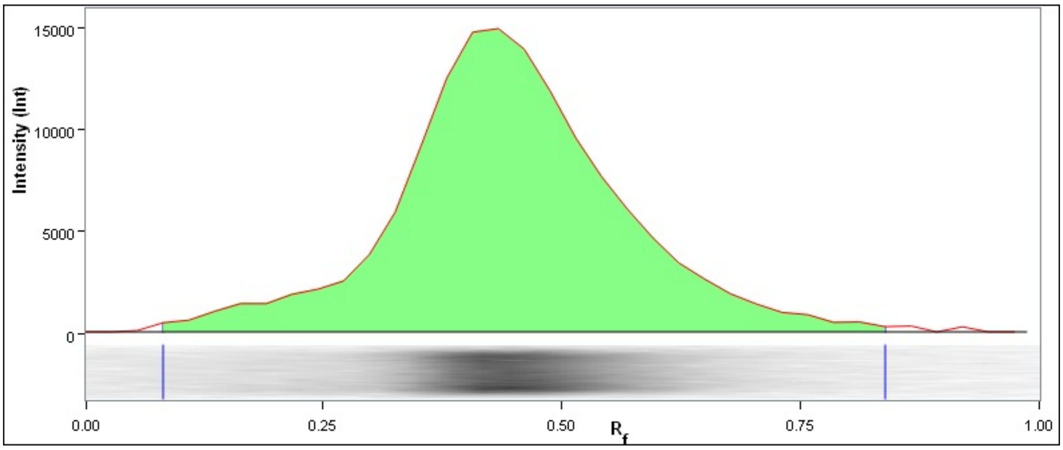

Band No.	Band Label	Mol. Wt. (KDa)	Relative Front	Adj. Volume (Int)	Volume (Int)	Abs. Quant.	Rel. Quant.	Band %	Lane %
1		N/A	0.459	6,144,876	8,882,424	N/A	N/A	100.0	99.2

Band Detection	Automatically detected bands with sensitivity: High
Lane Background	Lane background subtracted with disk size: 10
Lane Width	6.05 mm

Lane 2

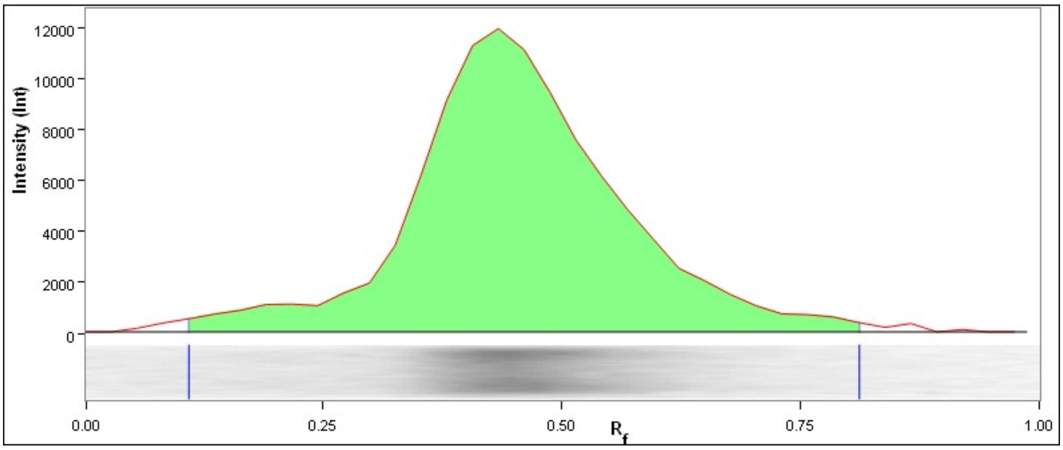

Band No.	Band Label	Mol. Wt. (KDa)	Relative Front	Adj. Volume (Int)	Volume (Int)	Abs. Quant.	Rel. Quant.	Band %	Lane %
1		N/A	0.459	3,935,727	6,430,082	N/A	N/A	100.0	98.5

Band Detection	Automatically detected bands with sensitivity: High
Lane Background	Lane background subtracted with disk size: 10
Lane Width	6.22 mm

Lane 3

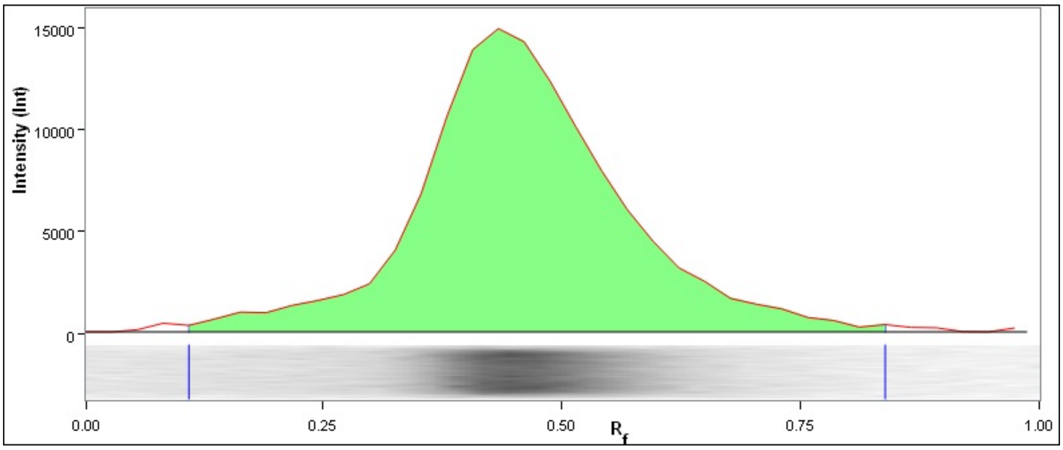

Band No.	Band Label	Mol. Wt. (KDa)	Relative Front	Adj. Volume (Int)	Volume (Int)	Abs. Quant.	Rel. Quant.	Band %	Lane %
1		N/A	0.459	5,541,550	8,395,660	N/A	N/A	100.0	99.1

Band Detection	Automatically detected bands with sensitivity: High
Lane Background	Lane background subtracted with disk size: 10
Lane Width	5.88 mm

Lane 4

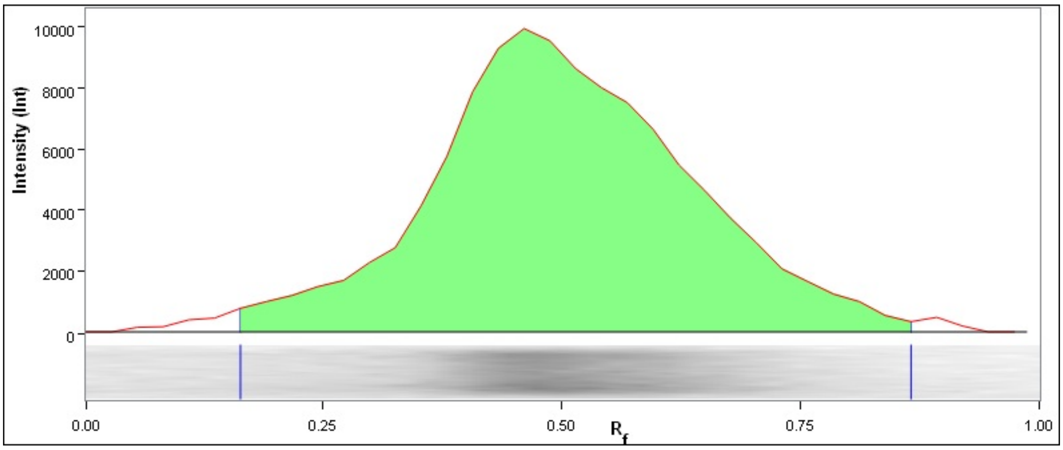

Band No.	Band Label	Mol. Wt. (KDa)	Relative Front	Adj. Volume (Int)	Volume (Int)	Abs. Quant.	Rel. Quant.	Band %	Lane %
1		N/A	0.486	4,626,994	8,084,120	N/A	N/A	100.0	98.1

Band Detection	Automatically detected bands with sensitivity: High

Lane Background	Lane background subtracted with disk size: 10
Lane Width	6.39 mm

Lane 5

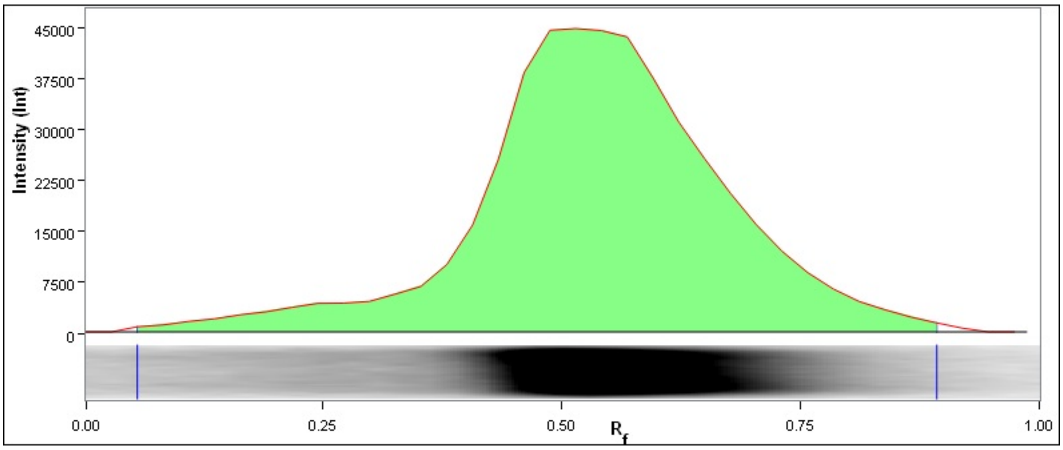

Band No.	Band Label	Mol. Wt. (KDa)	Relative Front	Adj. Volume (Int)	Volume (Int)	Abs. Quant.	Rel. Quant.	Band %	Lane %
1		N/A	0.541	18,816,455	26,592,300	N/A	N/A	100.0	99.8

Band Detection	Automatically detected bands with sensitivity: High
Lane Background	Lane background subtracted with disk size: 10
Lane Width	5.88 mm

Lane 6

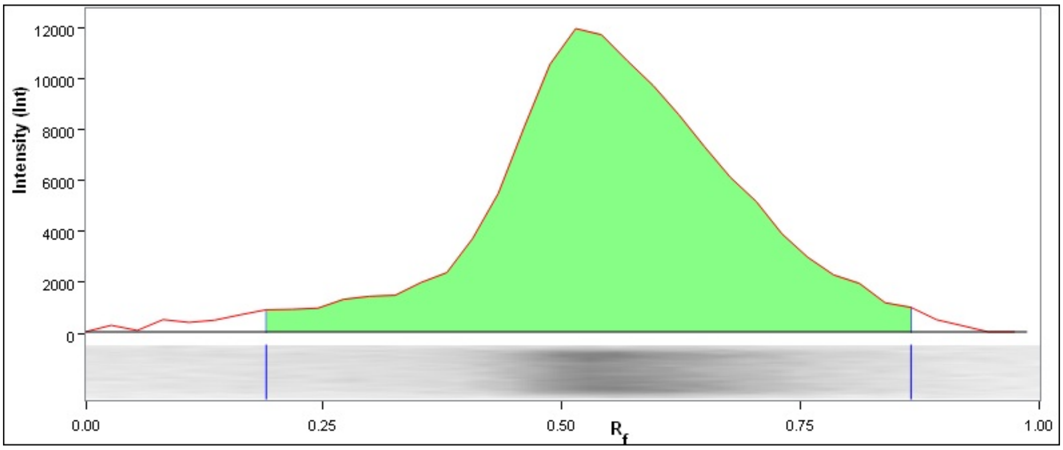

Band No.	Band Label	Mol. Wt. (KDa)	Relative Front	Adj. Volume (Int)	Volume (Int)	Abs. Quant.	Rel. Quant.	Band %	Lane %
1		N/A	0.568	4,694,671	8,151,507	N/A	N/A	100.0	97.3

Band Detection	Automatically detected bands with sensitivity: High
Lane Background	Lane background subtracted with disk size: 10
Lane Width	6.22 mm

Lane 7

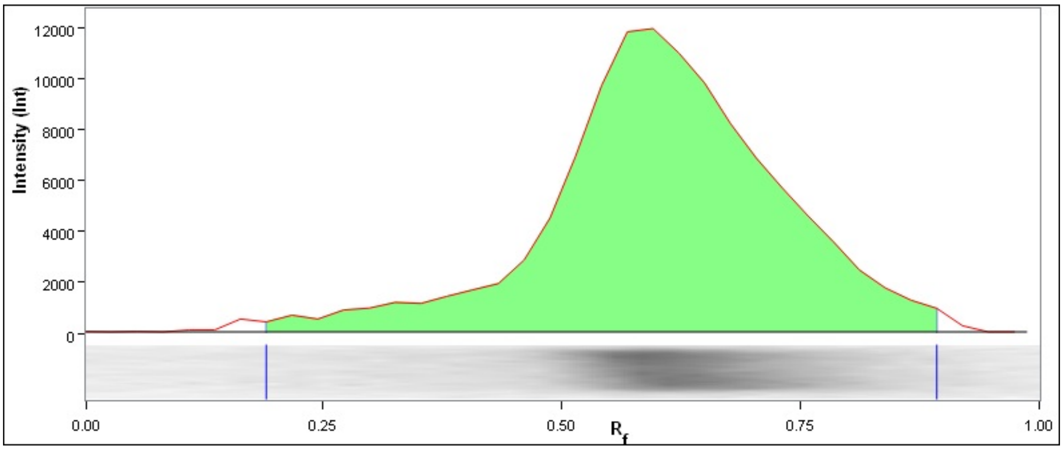

Band No.	Band Label	Mol. Wt. (KDa)	Relative Front	Adj. Volume (Int)	Volume (Int)	Abs. Quant.	Rel. Quant.	Band %	Lane %
1		N/A	0.622	4,602,564	7,857,864	N/A	N/A	100.0	99.1

Band Detection	Automatically detected bands with sensitivity: High
Lane Background	Lane background subtracted with disk size: 10
Lane Width	6.05 mm

Lane 8

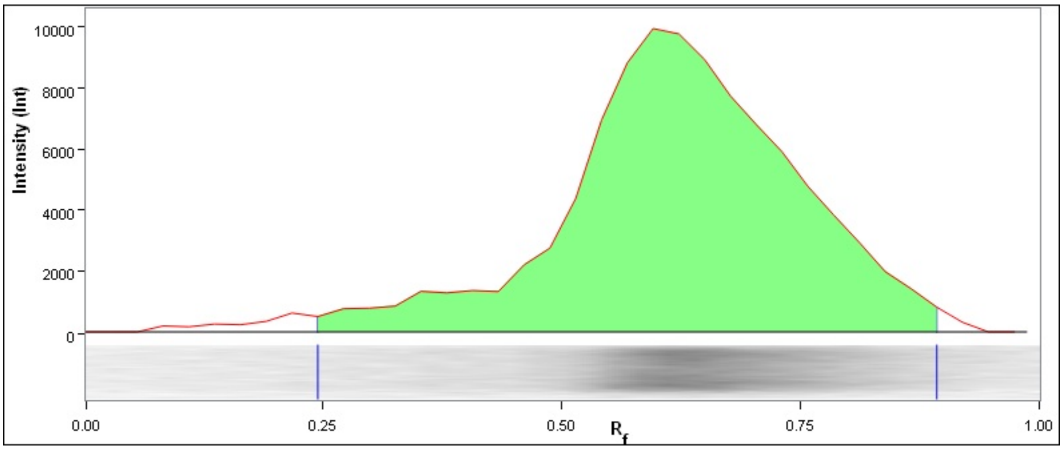

Band No.	Band Label	Mol. Wt. (KDa)	Relative Front	Adj. Volume (Int)	Volume (Int)	Abs. Quant.	Rel. Quant.	Band %	Lane %
1		N/A	0.649	4,200,018	7,591,734	N/A	N/A	100.0	98.2

Band Detection	Automatically detected bands with sensitivity: High
Lane Background	Lane background subtracted with disk size: 10
Lane Width	6.22 mm

Lane 9

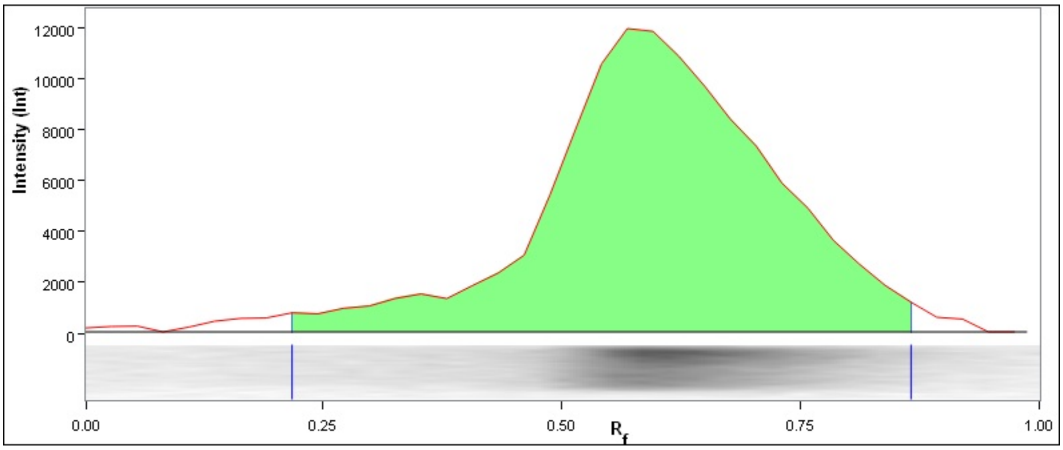

Band No.	Band Label	Mol. Wt. (KDa)	Relative Front	Adj. Volume (Int)	Volume (Int)	Abs. Quant.	Rel. Quant.	Band %	Lane %
1		N/A	0.622	4,953,153	7,725,193	N/A	N/A	100.0	97.1

Band Detection	Automatically detected bands with sensitivity: High
Lane Background	Lane background subtracted with disk size: 10
Lane Width	6.22 mm

Image Report: Gel3-Sol-SDHA_2016-10-04 12hr 41min_1sec-quant

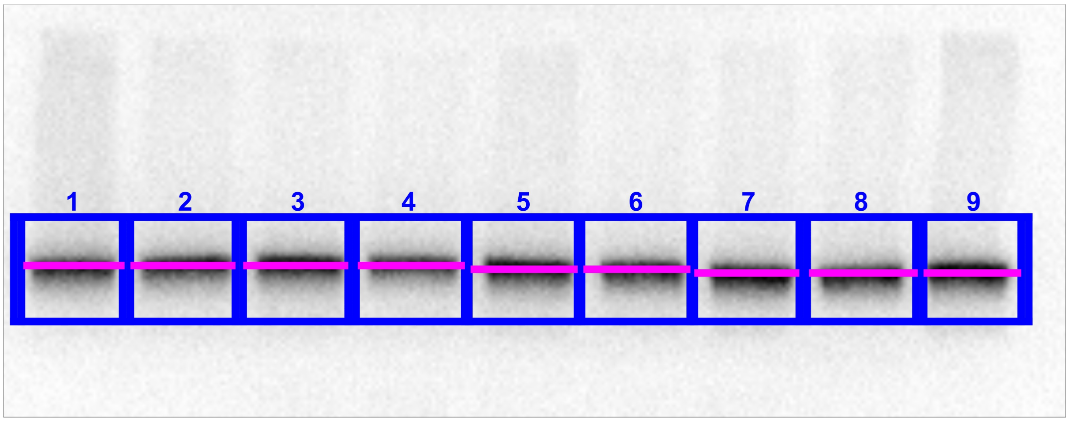

S:\RESGushchina_LiubovGushchina\LG Manuscripts\2026_MG29\ChemiDocs\Sol\Gel3-Sol-SDHA_2016-10-04 12hr 41min_1sec-quant.scn

Acquisition Information

Imager	ChemiDoc XRS+
Exposure Time (sec)	1.000 (Signal Accumulation)
Flat Field	Applied (Lens)
Serial Number	721BR04926
Software Version	5.2.1
Application	Chemi Hi Resolution
Excitation Source	No Illumination
Emission Filter	No Filter
Binning	2x2

Image Information

Acquisition Date	10/4/2016 12:41:45 PM
User Name	Gushchina
Image Area (mm)	X: 63.0 Y: 24.5
Pixel Size (µm)	X: 222.7 Y: 222.7
Data Range (Int)	0 - 12284

Analysis Settings

Detection	Lane detection:<br>Manually created lanes<br><br>Band detection:<br>Automatically detected bands with sensitivity: High<br>Manually adjusted bands<br><br>Lane Background Subtraction:<br>Lane background subtracted with disk size: 10<br><br>Lane width: Variable

Lane Statistics

Lane No.	Adj. Total Band Vol. (Int)	Total Band Vol. (Int)	Adj. Total Lane Vol. (Int)	Total Lane Vol. (Int)	Bkgd. Vol. (Int)	Norm. Factor
1	1,380,078	2,062,962	1,385,937	2,221,641	835,704	N/A
2	1,449,168	2,150,792	1,455,272	2,346,540	891,268	N/A
3	1,492,512	2,210,768	1,505,952	2,459,016	953,064	N/A
4	1,268,895	1,976,814	1,278,726	2,178,915	900,189	N/A
5	1,558,112	2,373,795	1,565,072	2,600,285	1,035,213	N/A
6	1,341,975	2,050,851	1,351,429	2,282,793	931,364	N/A
7	1,502,704	2,169,832	1,513,176	2,399,180	886,004	N/A
8	1,415,983	2,058,768	1,420,855	2,236,944	816,089	N/A
9	1,500,824	2,157,740	1,513,148	2,298,660	785,512	N/A

Lane And Band Analysis

Lane 1

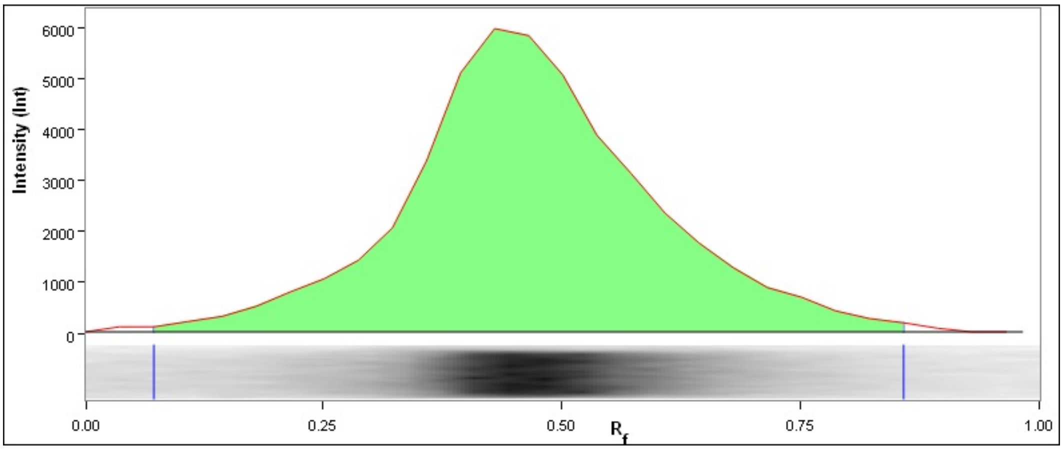

Band No.	Band Label	Mol. Wt. (KDa)	Relative Front	Adj. Volume (Int)	Volume (Int)	Abs. Quant.	Rel. Quant.	Band %	Lane %
1		N/A	0.464	1,380,078	2,062,962	N/A	N/A	100.0	99.6

Band Detection	Automatically detected bands with sensitivity: High
Lane Background	Lane background subtracted with disk size: 10
Lane Width	6.01 mm

Lane 2

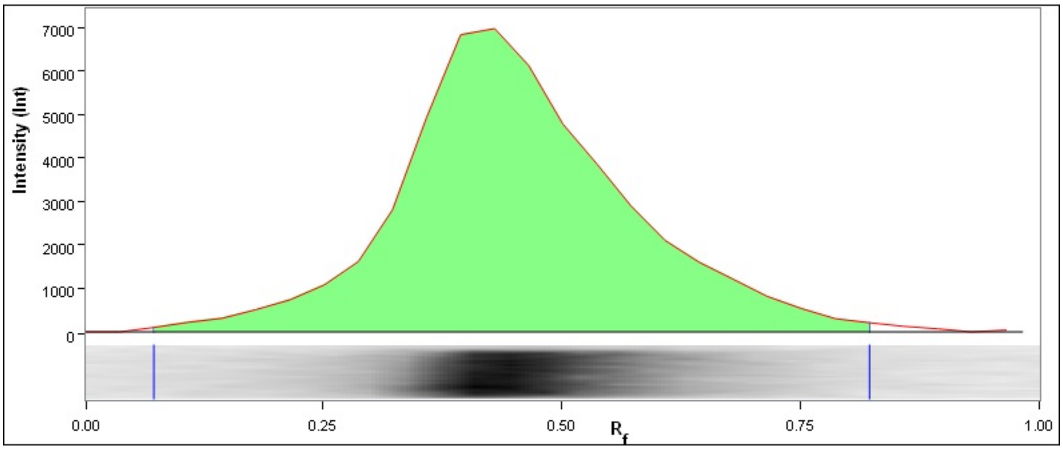

Band No.	Band Label	Mol. Wt. (KDa)	Relative Front	Adj. Volume (Int)	Volume (Int)	Abs. Quant.	Rel. Quant.	Band %	Lane %
1		N/A	0.464	1,449,168	2,150,792	N/A	N/A	100.0	99.6

Band Detection	Automatically detected bands with sensitivity: High
Lane Background	Lane background subtracted with disk size: 10
Lane Width	6.24 mm

Lane 3

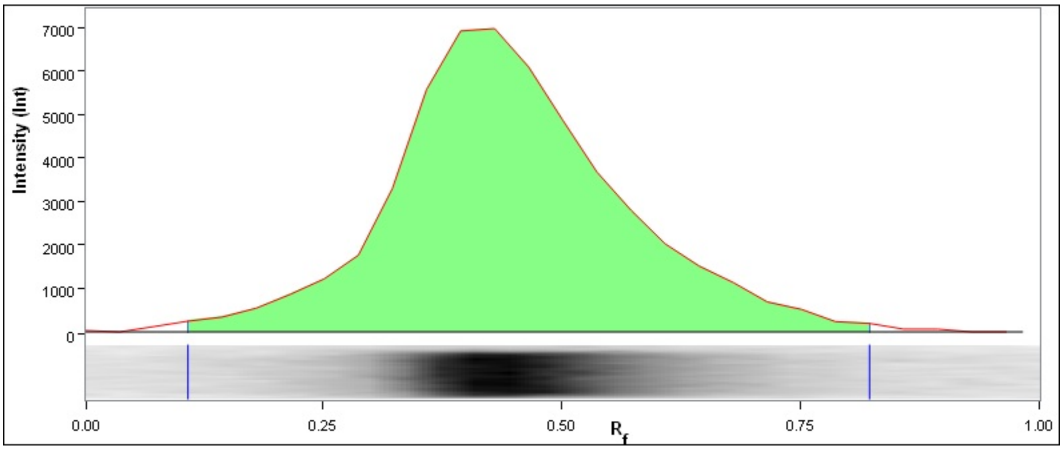

Band No.	Band Label	Mol. Wt. (KDa)	Relative Front	Adj. Volume (Int)	Volume (Int)	Abs. Quant.	Rel. Quant.	Band %	Lane %
1		N/A	0.464	1,492,512	2,210,768	N/A	N/A	100.0	99.1

Band Detection	Automatically detected bands with sensitivity: High
Lane Background	Lane background subtracted with disk size: 10
Lane Width	6.24 mm

Lane 4

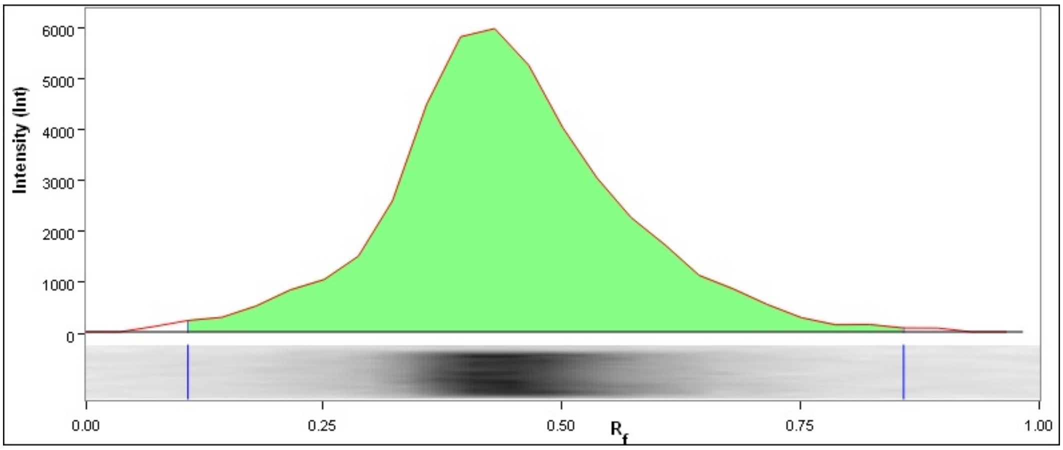

Band No.	Band Label	Mol. Wt. (KDa)	Relative Front	Adj. Volume (Int)	Volume (Int)	Abs. Quant.	Rel. Quant.	Band %	Lane %
1		N/A	0.464	1,268,895	1,976,814	N/A	N/A	100.0	99.2

Band Detection	Automatically detected bands with sensitivity: High
Lane Background	Lane background subtracted with disk size: 10
Lane Width	6.46 mm

Lane 5

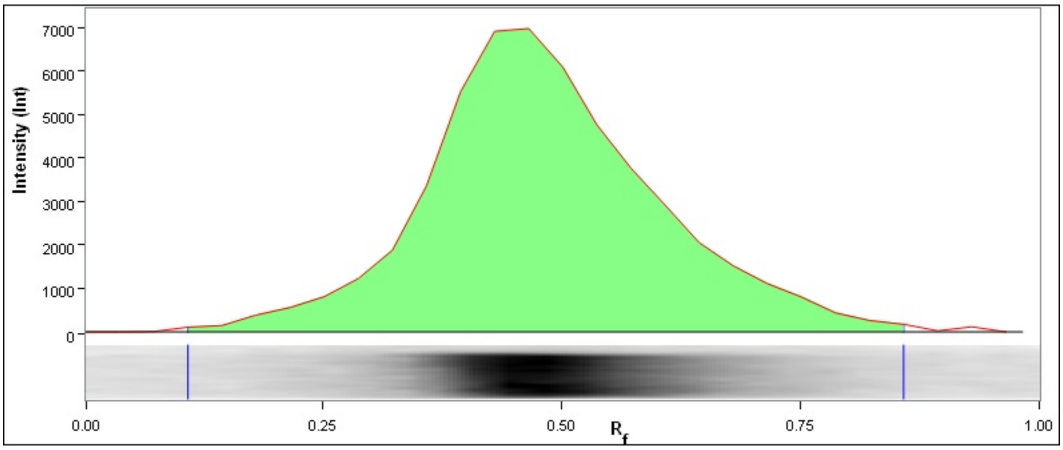

Band No.	Band Label	Mol. Wt. (KDa)	Relative Front	Adj. Volume (Int)	Volume (Int)	Abs. Quant.	Rel. Quant.	Band %	Lane %
1		N/A	0.500	1,558,112	2,373,795	N/A	N/A	100.0	99.6

Band Detection	Automatically detected bands with sensitivity: High
Lane Background	Lane background subtracted with disk size: 10
Lane Width	6.46 mm

Lane 6

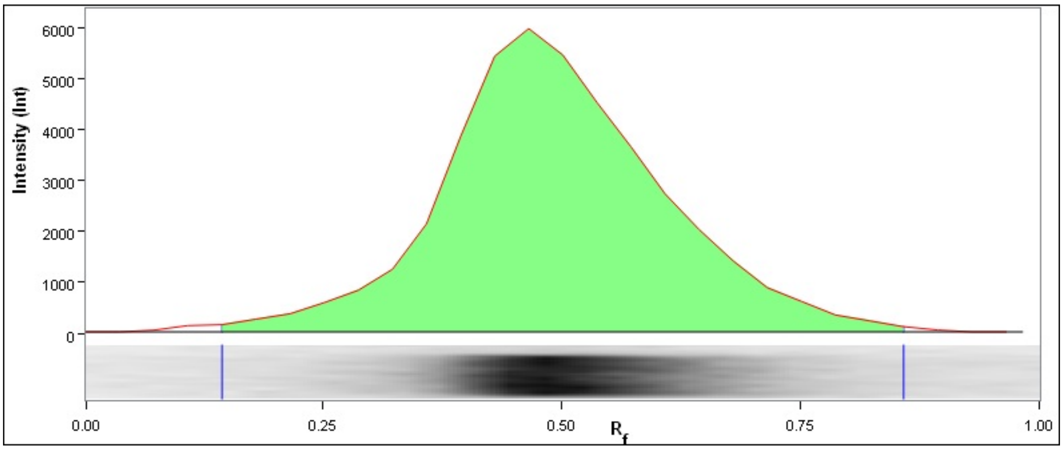

Band No.	Band Label	Mol. Wt. (KDa)	Relative Front	Adj. Volume (Int)	Volume (Int)	Abs. Quant.	Rel. Quant.	Band %	Lane %
1		N/A	0.500	1,341,975	2,050,851	N/A	N/A	100.0	99.3

Band Detection	Automatically detected bands with sensitivity: High
Lane Background	Lane background subtracted with disk size: 10
Lane Width	6.46 mm

Lane 7

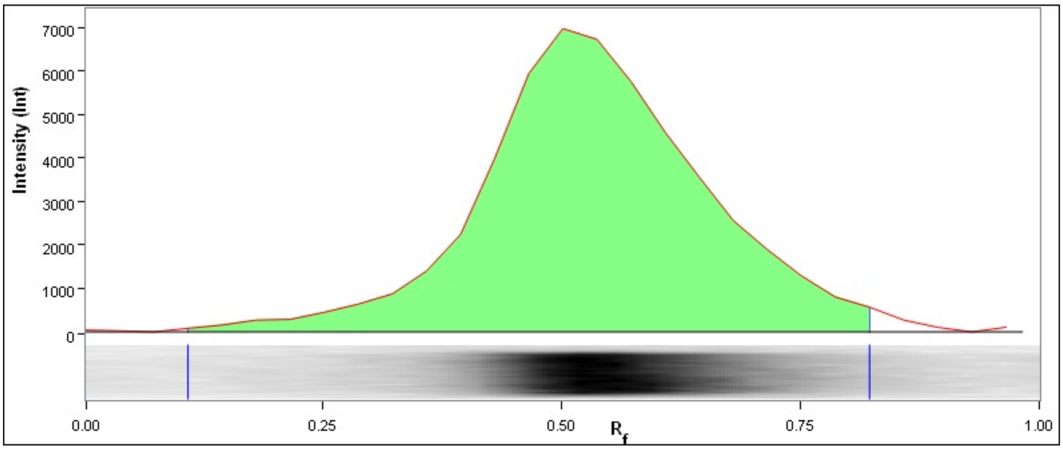

Band No.	Band Label	Mol. Wt. (KDa)	Relative Front	Adj. Volume (Int)	Volume (Int)	Abs. Quant.	Rel. Quant.	Band %	Lane %
1		N/A	0.536	1,502,704	2,169,832	N/A	N/A	100.0	99.3

Band Detection	Automatically detected bands with sensitivity: High
Lane Background	Lane background subtracted with disk size: 10
Lane Width	6.24 mm

Lane 8

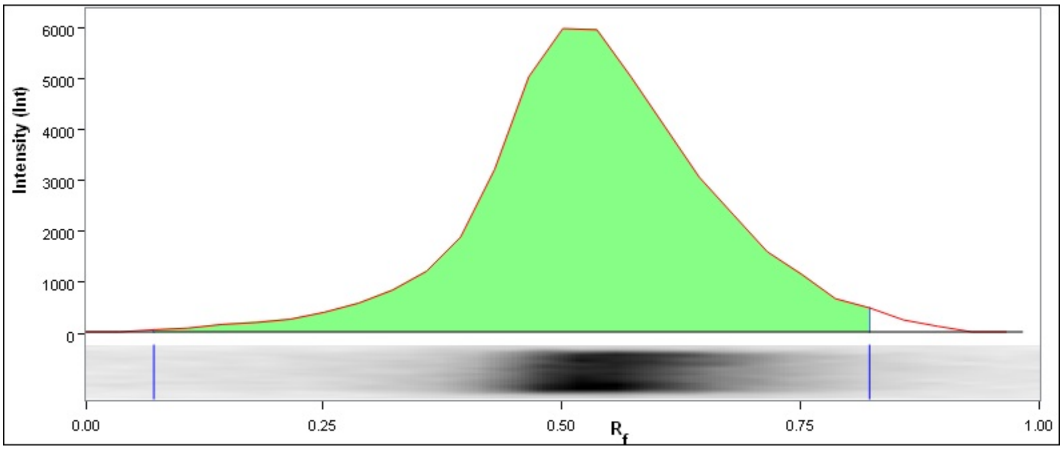

Band No.	Band Label	Mol. Wt. (KDa)	Relative Front	Adj. Volume (Int)	Volume (Int)	Abs. Quant.	Rel. Quant.	Band %	Lane %
1		N/A	0.536	1,415,983	2,058,768	N/A	N/A	100.0	99.7

Band Detection	Automatically detected bands with sensitivity: High
Lane Background	Lane background subtracted with disk size: 10
Lane Width	6.46 mm

Lane 9

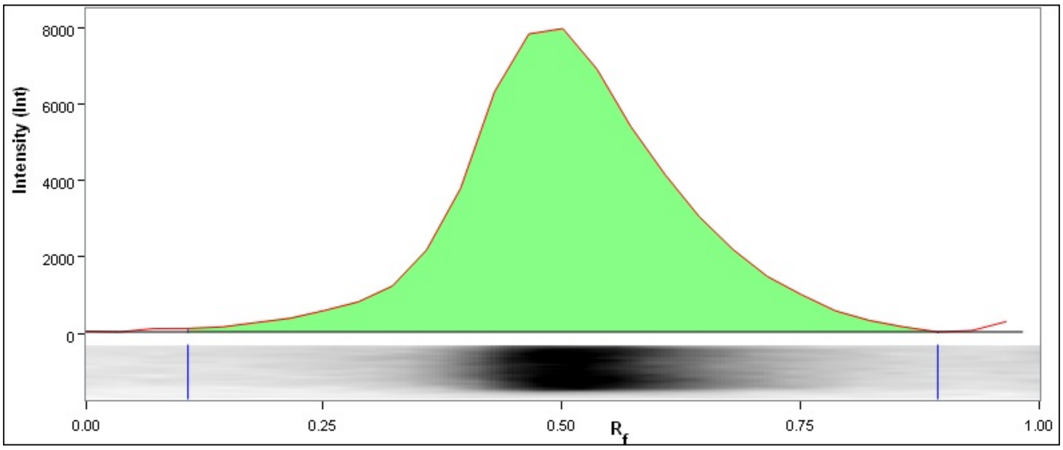

Band No.	Band Label	Mol. Wt. (KDa)	Relative Front	Adj. Volume (Int)	Volume (Int)	Abs. Quant.	Rel. Quant.	Band %	Lane %
1		N/A	0.536	1,500,824	2,157,740	N/A	N/A	100.0	99.2

Band Detection	Automatically detected bands with sensitivity: High
Lane Background	Lane background subtracted with disk size: 10
Lane Width	5.79 mm

Image Report: Gel3-Sol-GAPDH_2016-10-04 15hr 36min_50sec-quant

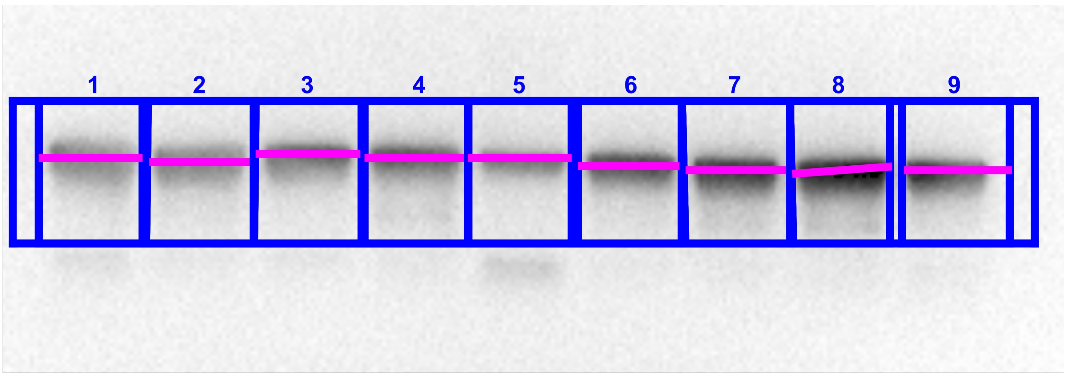

S:\RESGushchina_LiubovGushchina\LG Manuscripts\2026_MG29\ChemiDocs\Sol\Gel3-Sol-GAPDH_2016-10-04 15hr 36min_50sec-quant.scn

Acquisition Information

Imager	ChemiDoc XRS+
Exposure Time (sec)	50.000 (Signal Accumulation)
Flat Field	Applied (Lens)
Serial Number	721BR04926
Software Version	5.2.1
Application	Chemi Hi Resolution
Excitation Source	No Illumination
Emission Filter	No Filter
Binning	2x2

Image Information

Acquisition Date	10/4/2016 3:37:43 PM
User Name	Gushchina
Image Area (mm)	X: 66.9 Y: 23.1
Pixel Size (µm)	X: 251.4 Y: 251.4
Data Range (Int)	0 - 65535

Analysis Settings

Detection	Lane detection:<br>Manually created lanes<br><br>Band detection:<br>Automatically detected bands with sensitivity: High<br>Manually adjusted bands<br><br>Lane Background Subtraction:<br>Lane background subtracted with disk size: 10<br><br>Lane width: Variable

Lane Statistics

Lane No.	Adj. Total Band Vol. (Int)	Total Band Vol. (Int)	Adj. Total Lane Vol. (Int)	Total Lane Vol. (Int)	Bkgd. Vol. (Int)	Norm. Factor
1	6,222,736	9,614,774	6,255,158	10,491,286	4,236,128	N/A
2	6,582,708	10,129,833	6,674,805	11,264,049	4,589,244	N/A
3	6,966,243	10,732,203	7,036,146	11,895,336	4,859,190	N/A
4	7,418,658	11,069,344	7,804,914	13,314,886	5,509,972	N/A
5	4,713,332	8,764,262	4,775,966	10,218,026	5,442,060	N/A
6	8,130,746	12,177,802	8,195,512	13,431,080	5,235,568	N/A
7	9,459,531	14,071,266	9,633,870	15,834,555	6,200,685	N/A
8	11,540,025	15,776,850	11,829,975	17,755,275	5,925,300	N/A
9	7,926,255	11,118,924	8,002,962	12,131,937	4,128,975	N/A

Lane And Band Analysis

Lane 1

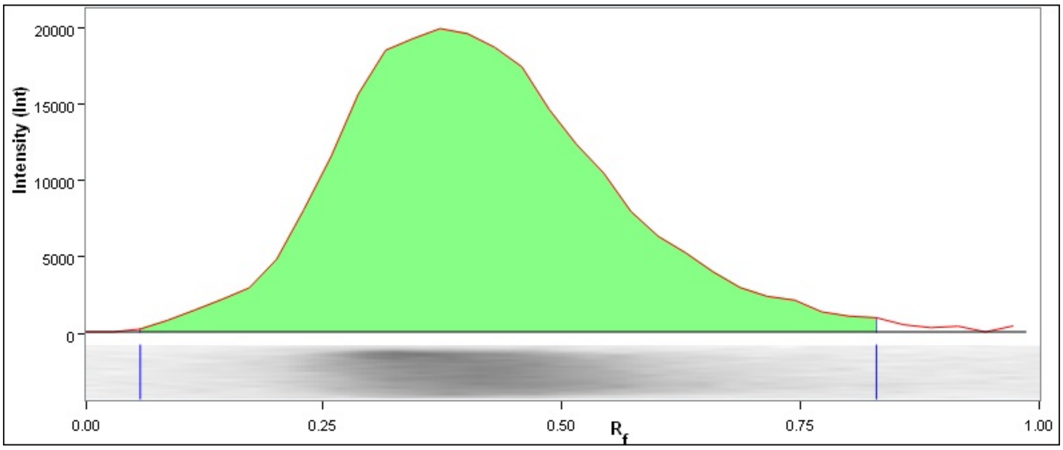

Band No.	Band Label	Mol. Wt. (KDa)	Relative Front	Adj. Volume (Int)	Volume (Int)	Abs. Quant.	Rel. Quant.	Band %	Lane %
1		N/A	0.400	6,222,736	9,614,774	N/A	N/A	100.0	99.5

Band Detection	Automatically detected bands with sensitivity: High
Lane Background	Lane background subtracted with disk size: 10
Lane Width	6.54 mm

Lane 2

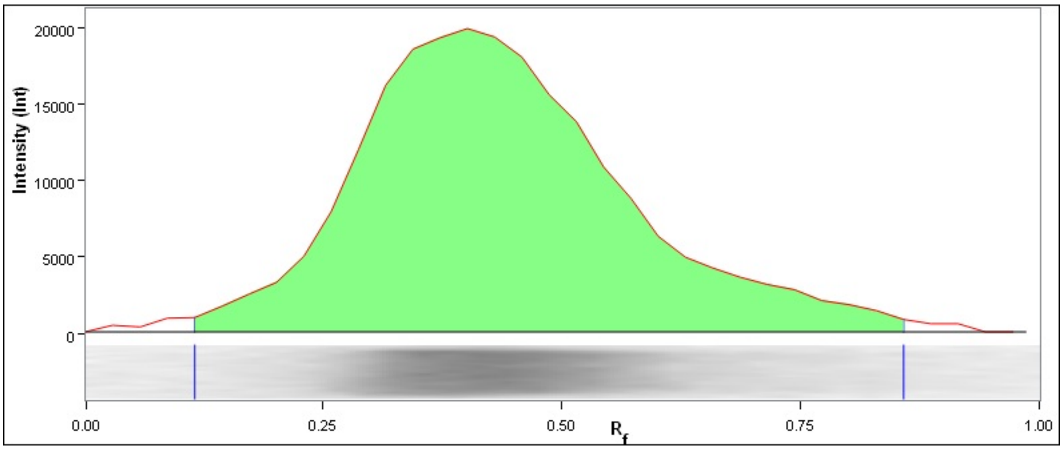

Band No.	Band Label	Mol. Wt. (KDa)	Relative Front	Adj. Volume (Int)	Volume (Int)	Abs. Quant.	Rel. Quant.	Band %	Lane %
1		N/A	0.429	6,582,708	10,129,833	N/A	N/A	100.0	98.6

Band Detection	Automatically detected bands with sensitivity: High
Lane Background	Lane background subtracted with disk size: 10
Lane Width	6.79 mm

Lane 3

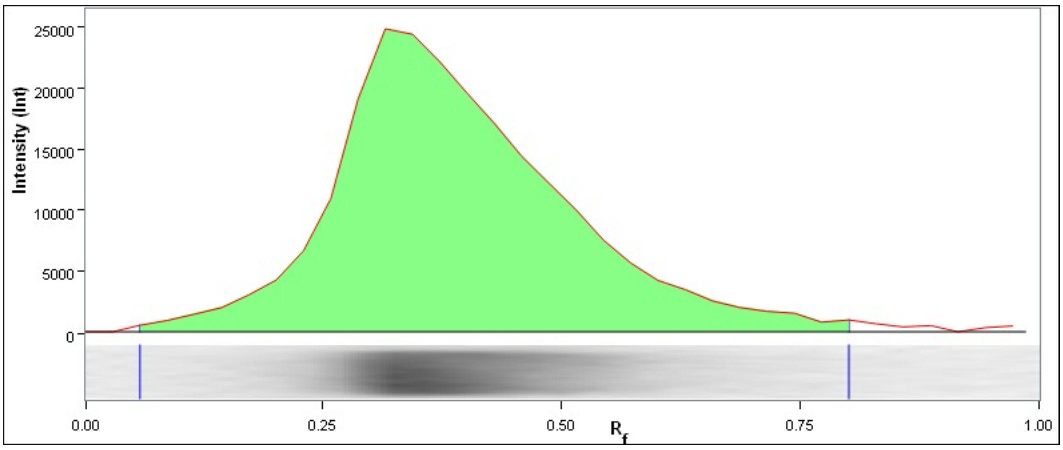

Band No.	Band Label	Mol. Wt. (KDa)	Relative Front	Adj. Volume (Int)	Volume (Int)	Abs. Quant.	Rel. Quant.	Band %	Lane %
1		N/A	0.371	6,966,243	10,732,203	N/A	N/A	100.0	99.0

Band Detection	Automatically detected bands with sensitivity: High
Lane Background	Lane background subtracted with disk size: 10
Lane Width	6.79 mm

Lane 4

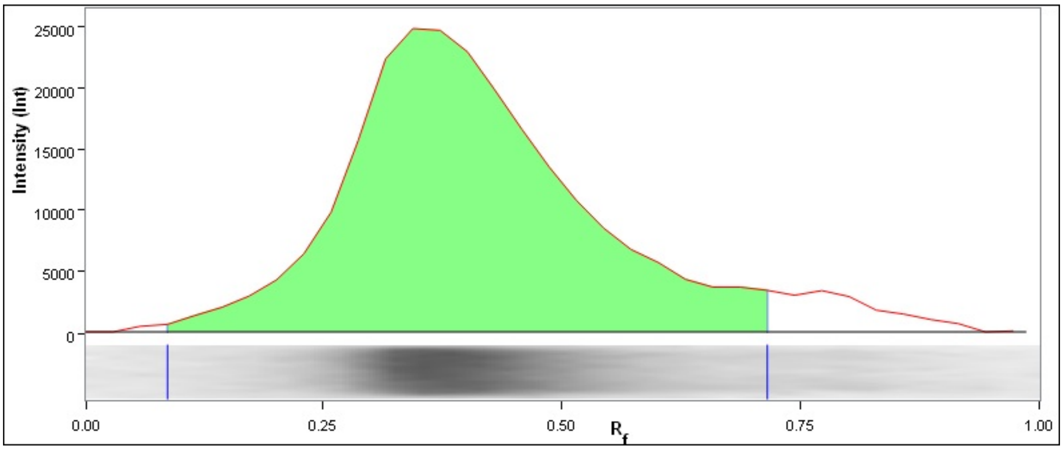

Band No.	Band Label	Mol. Wt. (KDa)	Relative Front	Adj. Volume (Int)	Volume (Int)	Abs. Quant.	Rel. Quant.	Band %	Lane %
1		N/A	0.400	7,418,658	11,069,344	N/A	N/A	100.0	95.1

Band Detection	Automatically detected bands with sensitivity: High

Lane Background	Lane background subtracted with disk size: 10
Lane Width	6.54 mm

Lane 5

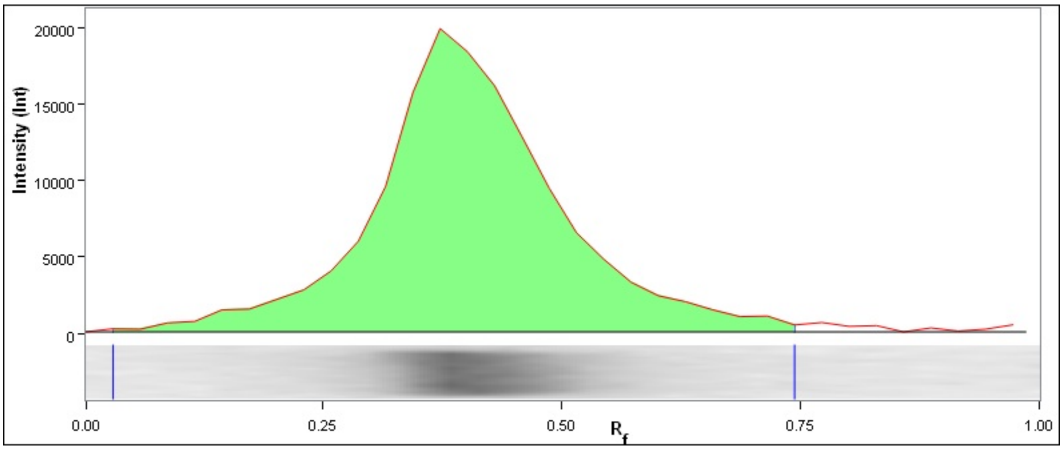

Band No.	Band Label	Mol. Wt. (KDa)	Relative Front	Adj. Volume (Int)	Volume (Int)	Abs. Quant.	Rel. Quant.	Band %	Lane %
1		N/A	0.400	4,713,332	8,764,262	N/A	N/A	100.0	98.7

Band Detection	Automatically detected bands with sensitivity: High
Lane Background	Lane background subtracted with disk size: 10
Lane Width	6.54 mm

Lane 6

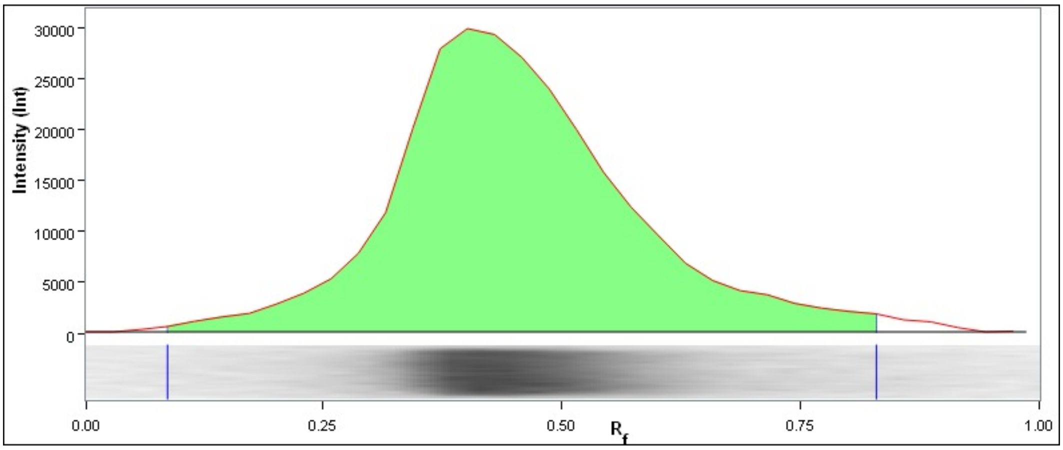

Band No.	Band Label	Mol. Wt. (KDa)	Relative Front	Adj. Volume (Int)	Volume (Int)	Abs. Quant.	Rel. Quant.	Band %	Lane %
1		N/A	0.457	8,130,746	12,177,802	N/A	N/A	100.0	99.2

Band Detection	Automatically detected bands with sensitivity: High
Lane Background	Lane background subtracted with disk size: 10
Lane Width	6.54 mm

Lane 7

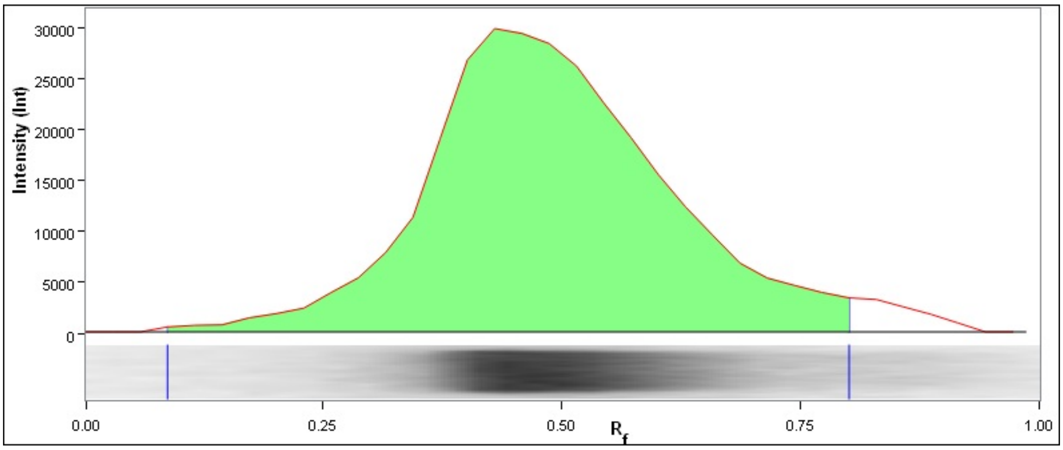

Band No.	Band Label	Mol. Wt. (KDa)	Relative Front	Adj. Volume (Int)	Volume (Int)	Abs. Quant.	Rel. Quant.	Band %	Lane %
1		N/A	0.486	9,459,531	14,071,266	N/A	N/A	100.0	98.2

Band Detection	Automatically detected bands with sensitivity: High
Lane Background	Lane background subtracted with disk size: 10
Lane Width	6.79 mm

Lane 8

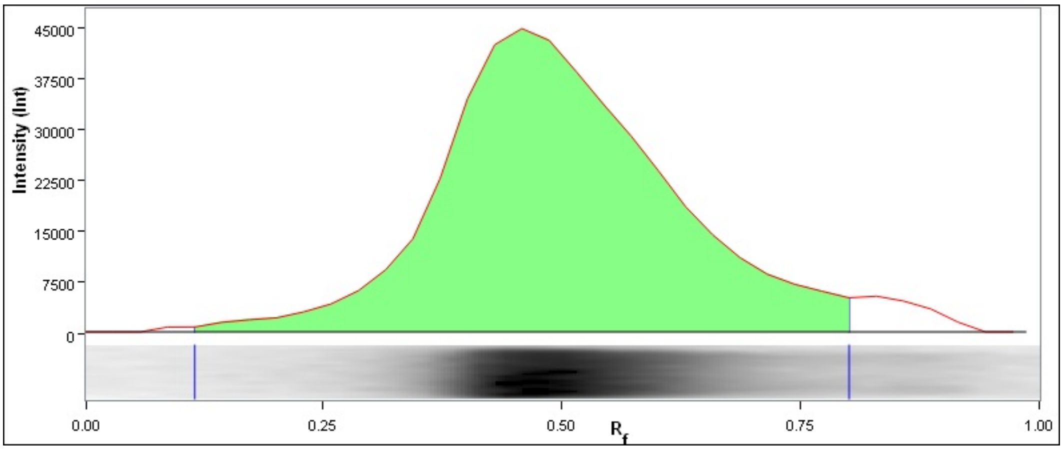

Band No.	Band Label	Mol. Wt. (KDa)	Relative Front	Adj. Volume (Int)	Volume (Int)	Abs. Quant.	Rel. Quant.	Band %	Lane %
1		N/A	0.486	11,540,025	15,776,850	N/A	N/A	100.0	97.5

Band Detection	Automatically detected bands with sensitivity: High
Lane Background	Lane background subtracted with disk size: 10
Lane Width	6.29 mm

Lane 9

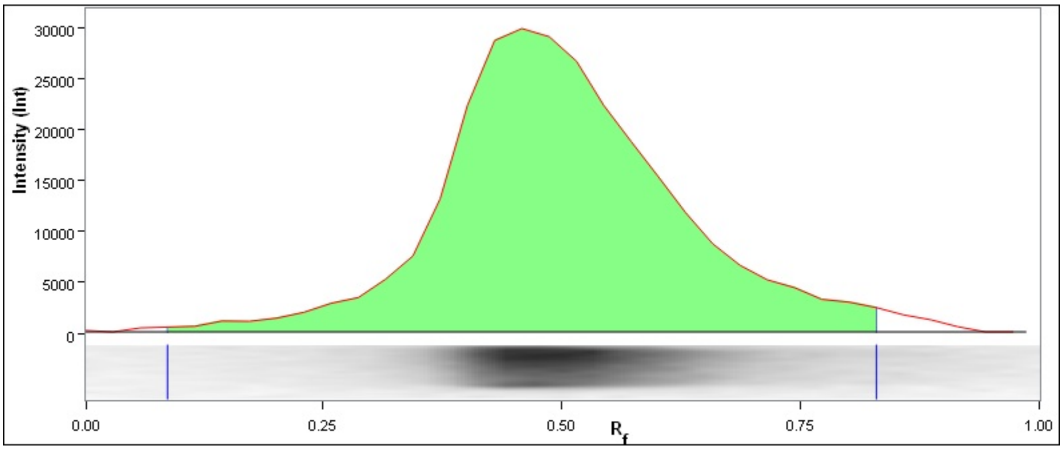

Band No.	Band Label	Mol. Wt. (KDa)	Relative Front	Adj. Volume (Int)	Volume (Int)	Abs. Quant.	Rel. Quant.	Band %	Lane %
1		N/A	0.486	7,926,255	11,118,924	N/A	N/A	100.0	99.0

Band Detection	Automatically detected bands with sensitivity: High
Lane Background	Lane background subtracted with disk size: 10
Lane Width	6.79 mm

Gel2-Sol-MG29_2016-10-04 12hr 58min_30sec-quant

Lane No.	Adj. Total Band Vol. (Int)	Total Band Vol. (Int)	Adj. Total Lane Vol. (Int)	Total Lane Vol. (Int)	Bkgd. Vol. (Int)	Norm. Factor
1	6144876	8882424	6191712	9770544	3578832	N/A
2	3935727	6430082	3997591	7516920	3519329	N/A
3	5541550	8395660	5591950	9325050	3733100	N/A
4	4626994	8084120	4715154	9578660	4863506	N/A
5	18816455	26592300	18846310	27875050	9028740	N/A
6	4694671	8151507	4825577	9772255	4946678	N/A
7	4602564	7857864	4646232	9034020	4387788	N/A
8	4200018	7591734	4277533	8926805	4649272	N/A
9	4953153	7725193	5100857	9163420	4062563	N/A

Gel3-Sol-SDHA_2016-10-04 12hr 41min_1sec-quant

Lane No.	Adj. Total Band Vol. (Int)	Total Band Vol. (Int)	Adj. Total Lane Vol. (Int)	Total Lane Vol. (Int)	Bkgd. Vol. (Int)	Norm. Factor
1	1380078	2062962	1385937	2221641	835704	N/A
2	1449168	2150792	1455272	2346540	891268	N/A
3	1492512	2210768	1505952	2459016	953064	N/A
4	1268895	1976814	1278726	2178915	900189	N/A
5	1558112	2373795	1565072	2600285	1035213	N/A
6	1341975	2050851	1351429	2282793	931364	N/A
7	1502704	2169832	1513176	2399180	886004	N/A
8	1415983	2058768	1420855	2236944	816089	N/A
9	1500824	2157740	1513148	2298660	785512	N/A

Gel3-Sol-GAPDH_2016-10-04 15hr 36min_50sec-quant

Lane No.	Adj. Total Band Vol. (Int)	Total Band Vol. (Int)	Adj. Total Lane Vol. (Int)	Total Lane Vol. (Int)	Bkgd. Vol. (Int)	Norm. Factor
1	6222736	9614774	6255158	10491286	4236128	N/A
2	6582708	10129833	6674805	11264049	4589244	N/A
3	6966243	10732203	7036146	11895336	4859190	N/A
4	7418658	11069344	7804914	13314886	5509972	N/A
5	4713332	8764262	4775966	10218026	5442060	N/A

6	8130746	12177802	8195512	13431080	5235568	N/A
7	9459531	14071266	9633870	15834555	6200685	N/A
8	11540025	15776850	11829975	17755275	5925300	N/A
9	7926255	11118924	8002962	12131937	4128975	N/A

	Soleus MG29/SHDA ratio	Norm. factor	Relative quant	Average	SD
Sol-6mo-01	4.4526	3.6271	1.2276	1.0000	0.2403
Sol-6mo-02	2.7159		0.7488		
Sol-6mo-03	3.7129		1.0237		
Sol-12mo-01	3.6465		1.0053	1.7664	1.3538
Sol-12mo-02	12.0764		3.3295		
Sol-12mo-03	3.4983		0.9645		
Sol-24mo-01	3.0629		0.8444	0.8574	0.0474
Sol-24mo-02	2.9662		0.8178		
Sol-24mo-03	3.3003		0.9099		

	Soleus MG29/GAPDH ratio	Norm. factor	Relative quant	Average	SD
Sol-6mo-01	0.9875	0.7936	1.2443	1.0000	0.2455
Sol-6mo-02	0.5979		0.7534		
Sol-6mo-03	0.7955		1.0024		
Sol-12mo-01	0.6237		0.7859	2.1813	2.4675
Sol-12mo-02	3.9922		5.0303		
Sol-12mo-03	0.5774		0.7275		
Sol-24mo-01	0.4866		0.6131	0.6197	0.1645
Sol-24mo-02	0.3640		0.4586		
Sol-24mo-03	0.6249		0.7874		
